# *meta*-C–H arylation of fluoroarenes *via* traceless directing group relay strategy†
†Electronic supplementary information (ESI) available. CCDC 1533208 and 1549121. For ESI and crystallographic data in CIF or other electronic format see DOI: 10.1039/c8sc02417k


**DOI:** 10.1039/c8sc02417k

**Published:** 2018-07-25

**Authors:** Marc Font, Andrew R. A. Spencer, Igor Larrosa

**Affiliations:** a School of Chemistry , University of Manchester , Oxford Road , Manchester , M13 9PL , UK . Email: igor.larrosa@manchester.ac.uk

## Abstract

While several methods for the *ortho* selective arylation of fluoroarenes, *meta*-functionalisation has never been achieved. We report a new methodology, based on the traceless directing group relay concept, leading to the first *meta*-selective (hetero)arylation of fluoroarenes. In this strategy, CO_2_ is introduced as a transient directing group, to control a Pd-catalysed arylation *meta* to the fluoro functionality, prior to its release in a sequential, one-pot fashion. This method has shown compatibility with a number of functional groups and substitution patterns in both the fluoroarene core and aryl iodide coupling partners, and proceeds with complete *meta*-selectivity and mono *vs.* bis-arylation selectivity.

## Abstract 2

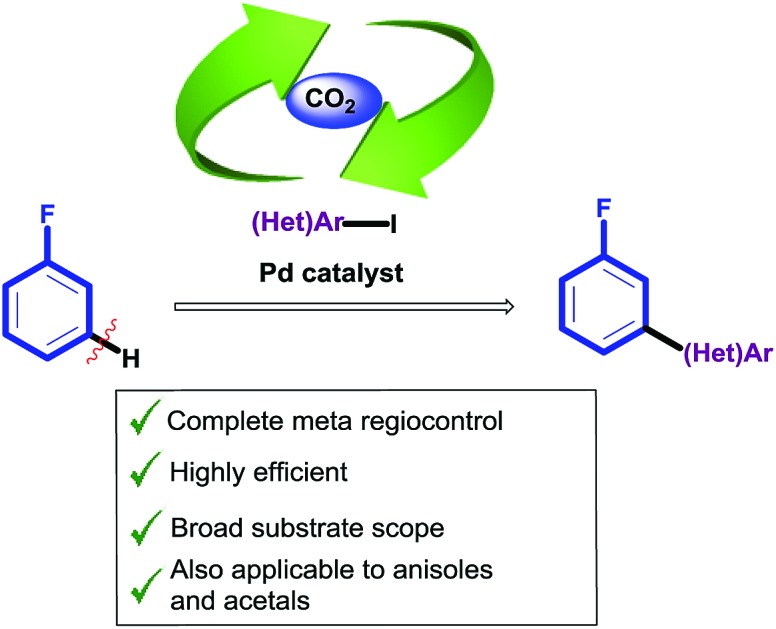
The first example of direct *meta*-C–H arylation of fluoroarenes to provide valuable *meta*-fluorobiaryls is achieved by exploiting CO_2_ as a transient directing group that enables complete regiochemical control of the arylation event.

## Introduction

**Fig. 1:**
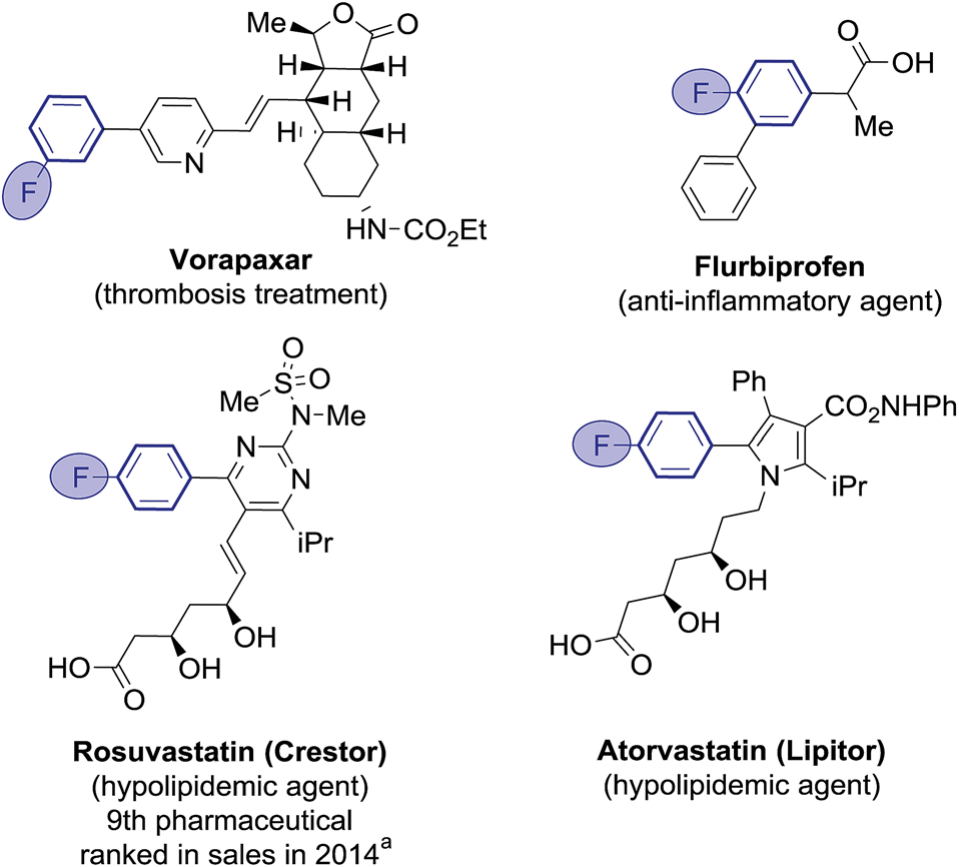
Examples of fluoro(heterobiaryl) motifs in pharmaceuticals. ^a^Source: EvaluatePharma/Vantage.

Fluoroarenes are recurring structural motifs in pharmaceuticals, agrochemicals, biological imaging agents and organic materials.^1^ So much so that up to 30% of pharmaceuticals and 40% of agrochemical agents currently contain at least one fluorine atom, usually located at arene rings (Fig. 1).1l,^2^ Fluorine is a bioisostere of hydrogen of unique biological relevance that can provide compounds with enhanced metabolic stability, bioavailability, lipophilicity and binding affinity among other properties.^2^,^3^ Fluoro(hetero)biaryl motifs are a particularly important class within fluorinated compounds, with widespread presence in pharmaceuticals, including top selling rosuvastatin and atorvastatin (Fig. 1).1i,^4^

Fluoro(hetero)biaryl motifs can be accessed by assembly of the arene fragments *via* traditional cross-coupling or by late installation of the fluorine atom from suitably prefunctionalised precursors.^5^,^6^ However, precursors such as aryl halides and arylboronic acids have an elevated cost compared to their parent fluoroarenes and/or need to be synthesised, adding several steps to the overall process. Very recently, Ritter and co-workers have developed a general method for aromatic C–H fluorination, however restricted to the *ortho* and *para* positions of arene rings.^7^

On the other hand, methods for the C–H arylation of (mono)fluoroarenes are rare and again limited to *ortho* or *para* selective arylation. Furthermore, current methods require large excesses of the fluoroarene (often used as cosolvent) to bolster the activation of their relatively inert C–H bonds.^8^

**Scheme 1:**
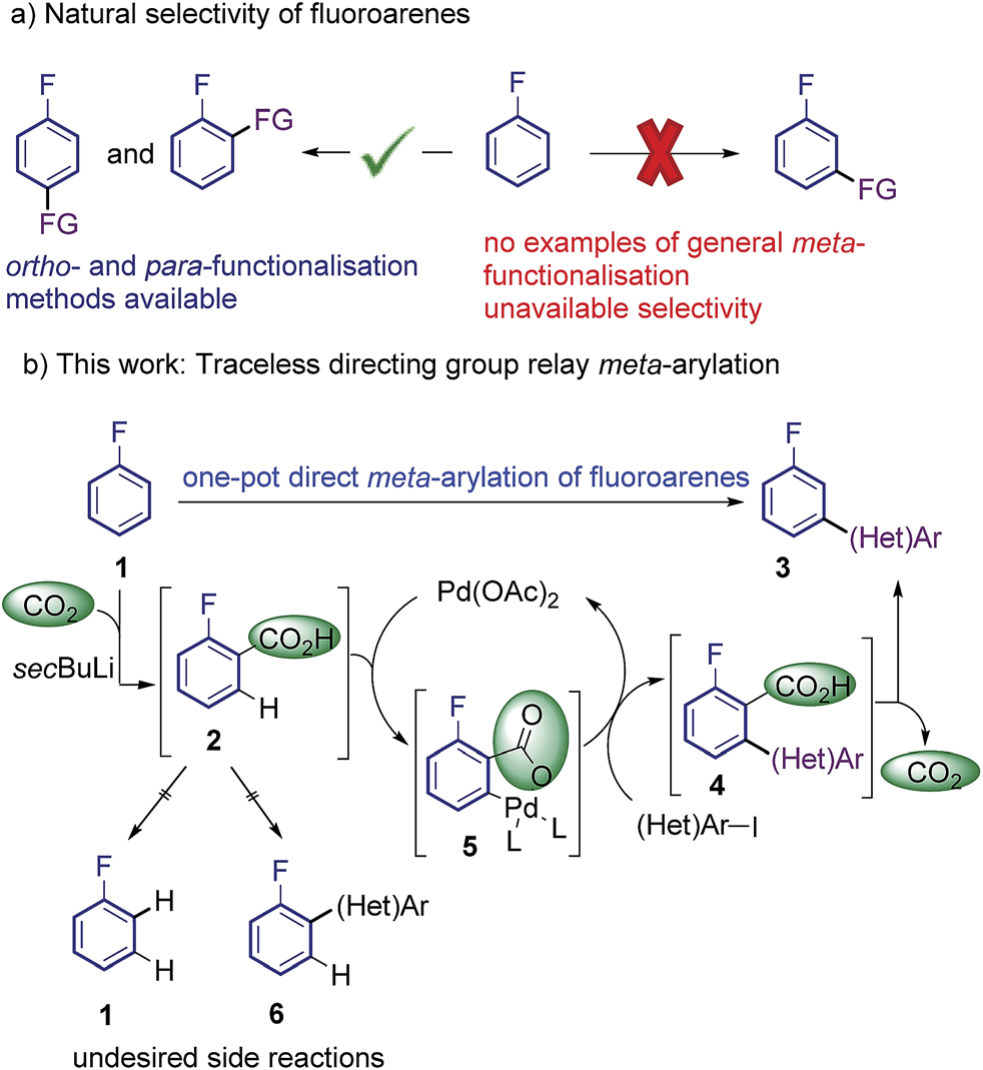
Proposed approach for regioselective *meta*-arylation of fluoroarenes.

Additional electron-withdrawing groups or π-complexation of a Cr(CO)_3_ fragment have been shown to result in improved reactivity towards *ortho*-arylation, allowing the use of these fluorobenzenes as limiting reagents.^9^,^10^ However, to date a methodology capable of overriding the inherent *ortho* and *para* reactivity of fluoroarenes, and affording general methods for the *meta*-selective arylation has been unattained.^11^ Thus, an effective and selective strategy for the *meta*-functionalisation of cheap and readily available fluoroarenes is highly desirable (Scheme 1a).

Current approaches for the selective *meta* C–H functionalisation of substituted arenes are scarce, generally relying on chelation-assistance and restricted to only a few classes of arenes bearing removable or non-removable directing groups.^12^,^13^ These strategies are not applicable to the fluoro functionality; hence an alternative approach is required. We envisioned that a traceless directing group relay strategy could be a suitable approach to provide the desired selectivity on fluoroarenes.^14^,^15^ Our working hypothesis (Scheme 1b) involved the installation of a temporary carboxylic acid group *ortho*-to-fluorine, which in turn would direct a palladium-catalyzed *ortho*-selective arylation^16^ (but *meta* relative to the fluorine group), prior to its cleavage. *ortho*-Carboxylation of fluorobenzenes has previously been achieved *via* directed *ortho*-metalation (D*o*M) with organolithiums and CO_2_.^17^

Indeed, one-pot D*o*M/cross-coupling sequences have proven successful for the rapid access to *ortho*-aryl fluorobenzenes.^18^–^20^ On the other hand, transition metal-catalysed protodecarboxylations have been extensively studied over the last decade and a vast repository of methods are available, making carboxylic acids ideal traceless directing groups.15d,^21^

The proposed approach poses several crucial challenges that have to be overcome in order to achieve the desired *meta*-selective arylation: (1) high yielding carboxylation reactions are needed. (2) Highly hindered adducts **4** need to readily undergo protodecarboxylation under the reaction conditions. (3) Undesired protodecarboxylation of benzoic acids **2** leading to fluoroarenes **1** has to be minimised. (4) Alternative decarboxylative *ipso*-arylation processes, which would lead to *ortho*-arylated fluoroarenes **6** have to be avoided.

## Results and discussion

**Scheme 2:**
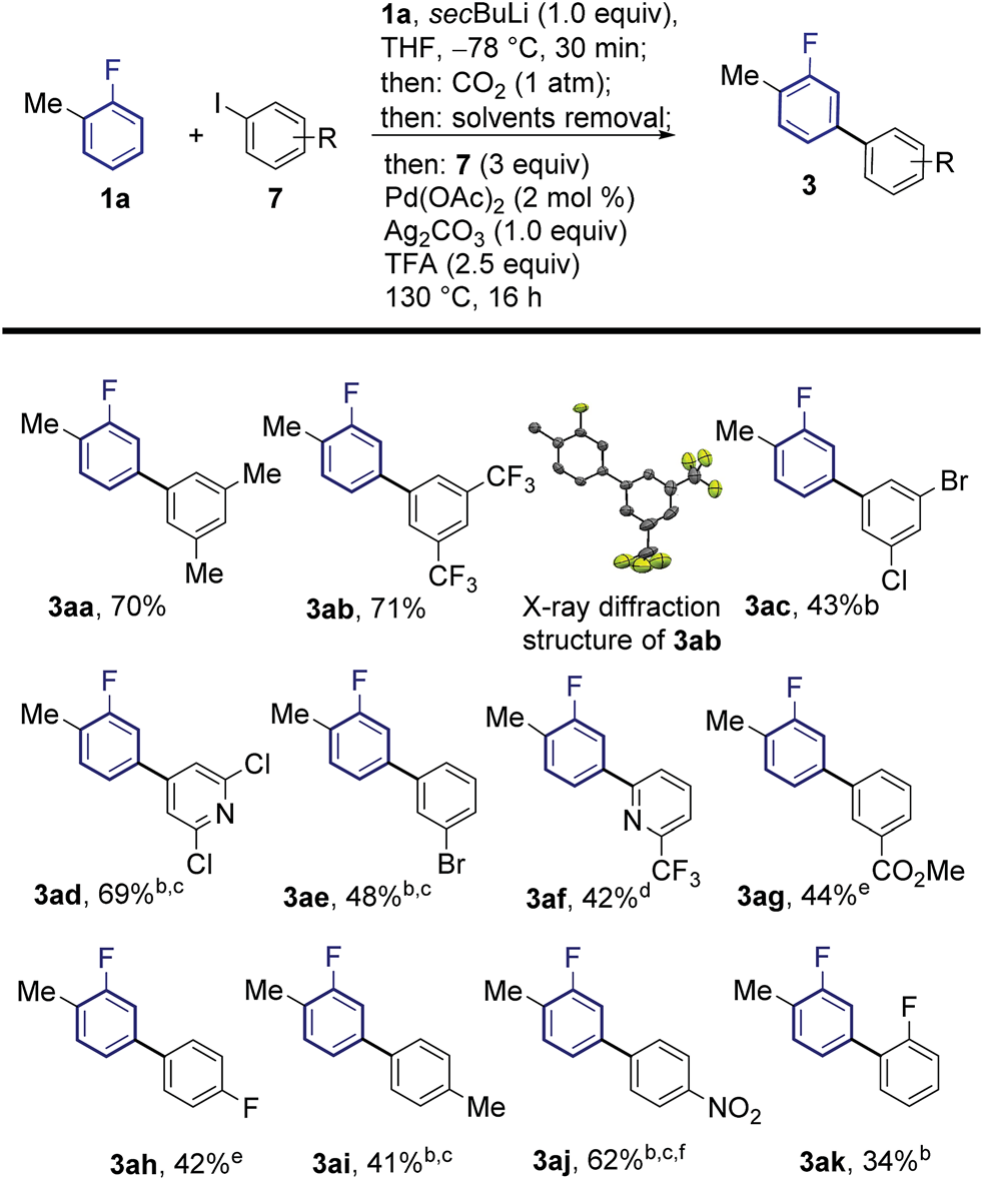
Substrate scope of the *meta*-arylation of fluoroarenes with aryl iodides. ^a^Yields are of pure isolated product. ^b^After 16 h, 2 mL of DMSO were added and the reaction was stirred at 170 °C for 3 h. ^c^1.5 equiv. of **7** employed. ^d^0.5 equiv. of **7**, 2 mol% Pd(OAc)_2_ and 0.5 equiv. of Ag_2_CO_3_ were employed. ^e^2 equiv. of **7** employed for 10 more hours. ^f^Arylation/decarboxylation step run at 150 °C.

The feasibility of this strategy was first assessed for the coupling of 2-fluorotoluene (**1a**) with 5-iodo-*meta*-xylene (**7a**, Table 1). Optimisation of the lithiation/carboxylation step, revealed that quantitative formation of 3-methyl-2-fluorobenzoic acid could be achieved by using *sec*BuLi and CO_2_ at atmospheric pressure. We then turned our attention to optimising the tandem arylation/protodecarboxylation process in order to obtain the desired *meta*-arylated fluoroarene products in a one-pot process. Examination of reaction conditions previously developed for the tandem arylation/decarboxylation of benzoic acids14*c* gave mixtures of the desired *meta*-arylation product **3aa** and the corresponding non-decarboxylated arylation product **4aa** even at high temperatures (entries 1 and 2). It has been shown that alkali carbonates can prevent protodecarboxylation in the *ortho*-arylation of benzoic acids.^22^ We hypothesised that the lithium benzoate formed in the lithiation/carboxylation step was responsible for the sluggish decarboxylation of **4aa**. Replacement of acetic acid for isobutyric and pivalic acid afforded almost no **3aa** product (entries 3 and 4). We then turned our attention to stronger carboxylic acids, which have proven beneficial in other Pd-catalysed protodecarboxylation reactions.^23^ Indeed, trifluoroacetic acid (TFA) led to 63% of the desired product **3aa**, although still 19% of non-decarboxylated product **4aa** was present after the reaction (entry 5). Gratifyingly, reducing the amount of TFA to only 2.5 equiv. led to 72% of the product with nearly complete decarboxylation of **4aa** (entry 6).^24^ Importantly, the product was formed with complete *meta*-regioselectivity showing no traces of *para*- or *ortho*-arylation products. With the optimised conditions in hand we set out to investigate the generality of the methodology with respect to the aryl iodide coupling partner. The developed reaction conditions tolerate electron-donating and electron-withdrawing groups in the *ortho*, *meta* and *para* positions of the aryl iodide (Scheme 2).

**Table 1:** Optimisation of the one-pot *meta*-arylation protocol^*a*^

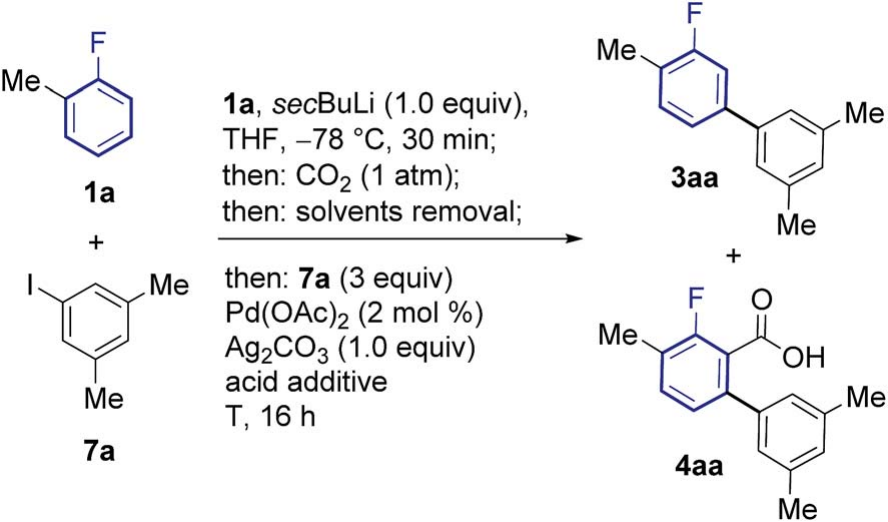				
Entry	Acid additive (equiv.)	*T* (°C)	**3aa** (%)	**4aa** (%)
1	AcOH (3.5)	130	30	34
2	AcOH (3.5)	150	42	16
3	*i*PrCOOH (3.5)	130	3	51
4	PivOH (3.5)	130	5	53
5	TFA (3.5)	130	63	19
**6**	**TFA (2.5)**	**130**	**72**	**1**
7	TFA (4)	130	45	23

^*a*^Yields were determined by ^19^F NMR analysis using 4-bromofluorobenzene as an internal standard.

**Scheme 3:**
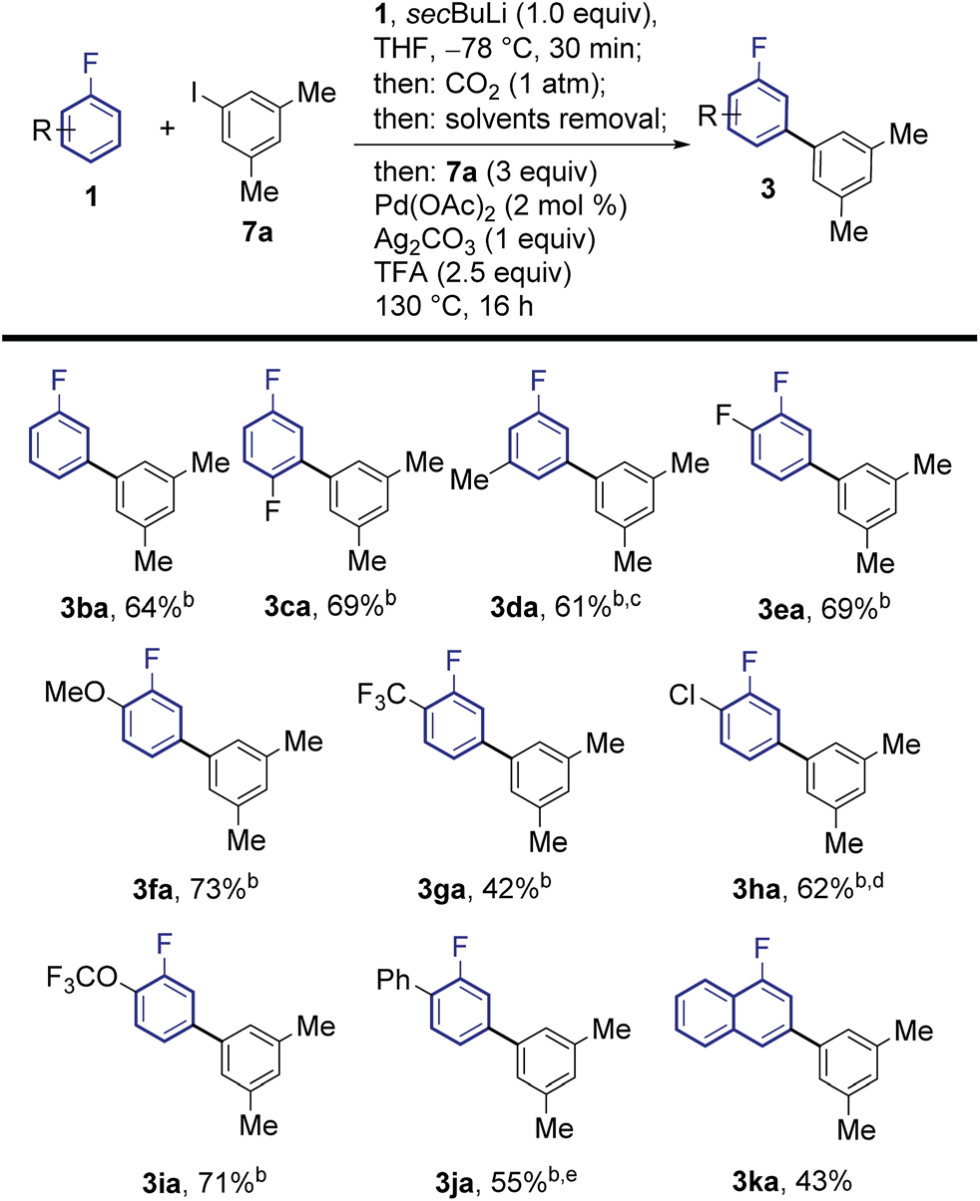
Substrate scope of the *meta*-arylation of fluoroarenes with aryl iodides. ^a^Yields are of pure isolated product. ^b^After 16 h, 2 mL of DMSO were added and the reaction was stirred at 170 °C for 3 h. ^c^0.5 equiv. **7a**, 2 mol% Pd(OAc)_2_ and 0.5 equiv. of Ag_2_CO_3_ were employed. ^d^After 6 h of arylation/decarboxylation reaction, an additional 2 mol% Pd(OAc)_2_ was added and the reaction was stirred at 130 °C for 10 more hours. ^e^1.5 equiv. **7a**.

The regiochemical outcome of the reaction was confirmed by X-ray diffraction analysis of compound **3ab**. Aryl iodides bearing nitro, methyl, ester and trifluoromethyl functionalities were compatible with this methodology. Halogen substituents were also compatible (**3ac**, **3ad**, **3ae**) serving as handles for further functionalisation of the products. Remarkably, 2-substituted pyridyl iodides are suitable coupling partners affording the corresponding *meta*-(hetero)arylfluoroarene products **3ad** and **3af**. Pd-catalysed protodecarboxylation is usually disfavoured for electron-poor benzoic acids,^23^ accordingly, some of the more electron-poor arylated fluorobenzoic acids underwent sluggish decarboxylation. This issue could be resolved by adding DMSO and heating for additional 3 h, triggering a Ag-catalysed decarboxylation, which is favourable on these substrates.^25^ This led to complete protodecarboxylation of **4**, thus providing the desired *meta*-fluorobiaryl products **3**. We then explored the effect of substitution at the fluoroarene core (Scheme 3). Pleasingly, the one-pot *meta*-arylation protocol showed compatibility with *ortho*, *meta* and *para* substitution patterns in the fluoroarene ring, while maintaining complete *meta*-regioselectivity for the arylation. Furthermore, bis-arylation products, a common and generally undesired pathway in many *meta*-C–H functionalization protocols were completely suppressed. This one-pot method afforded the *meta*-aryl fluorobenzene **3ba** in comparable yields to the reaction starting from fluorobenzoic acid.14*c*

Fluoroarenes bearing other functional groups known to induce directed *ortho*-metalation (D*o*M) reactions15b,^26^ (**3fa**, **3ga**, **3ia**) afforded only the desired *meta*-to-fluorine products showing the prevalence of fluorine to direct the lithiation step over other functional groups. 1-Chloro-2-fluorobenzene afforded the corresponding desired product **3ha** with no traces of dechlorinated side products. Our approach was also compatible with highly electron-poor fluoroarene rings (**3ea**, **3ga**). The previously reported *meta*-arylated fluorobenzene (**3ba**) could also be prepared using this method.

**Scheme 4:**
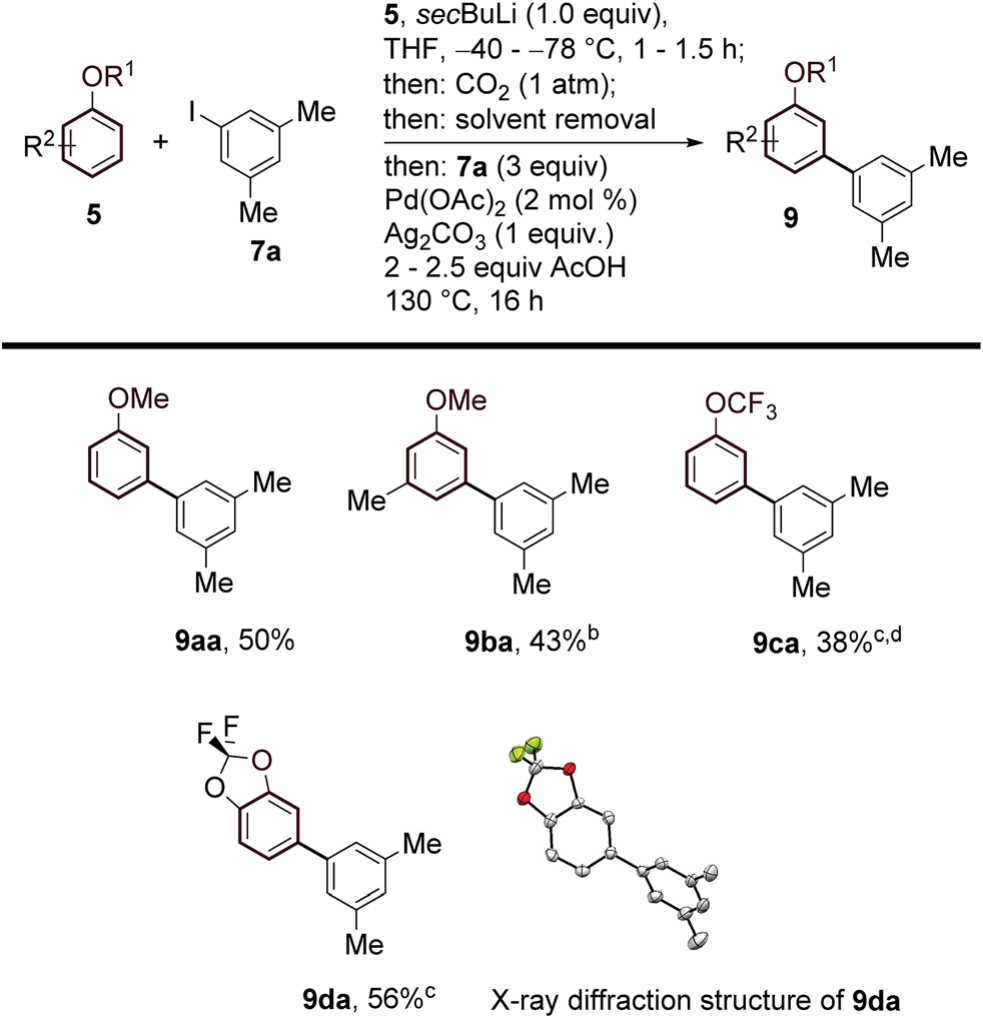
Substrate scope of the *meta*-arylation of substituted arenes with aryl iodides. ^a^Yields are of pure isolated product. ^b^0.5 equiv. **7a**, 2 mol% Pd(OAc)_2_ and 0.5 equiv. of Ag_2_CO_3_ were employed. ^c^After 16 h, 2 mL of DMSO were added and the reaction was stirred at 170 °C for 3 h. ^c^0.5 equiv. **7a**, 2 mol% Pd(OAc)_2_ and 0.5 equiv. of Ag_2_CO_3_ were employed. ^d^TFA used instead of AcOH.

Aryl ethers are useful, frequent intermediates in organic synthesis and are found in an impressive number of biologically active compounds and natural products.^27^ Pleasingly, we were also able to extend the scope of our protocol to this class of substrates (Scheme 4). After careful screening of reaction conditions for both the lithiation/carboxylation and arylation/decarboxylation steps, we found optimised conditions allowing the one-pot *meta*-arylation of anisoles, to form **9aa**,14*c* and **9ba**. Perfluorinated aryl alkyl ethers and acetals also showed good compatibility with our method and afforded the corresponding products **9ca** and **9da** with complete selectivity.

**Scheme 5:**
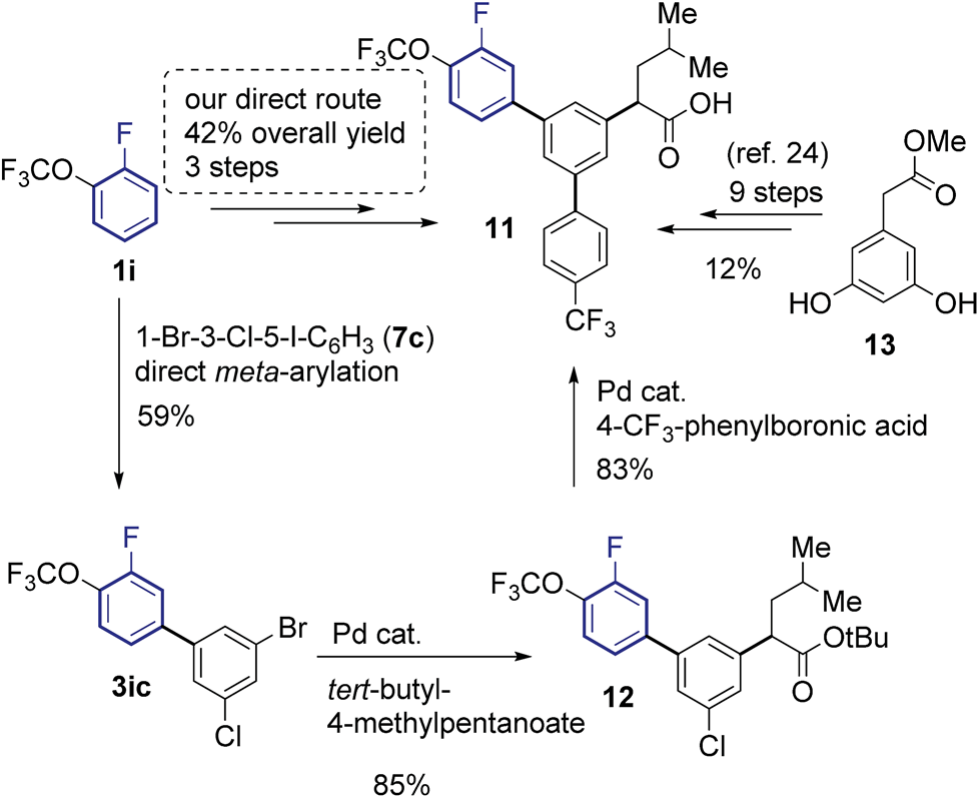
Synthesis of γ-secretase inhibitor **11**.

Since the developed method provides fast and selective access to *meta*-arylfluoroarenes, it can accelerate and lower the costs of synthesising compounds with such motifs. Indeed, the synthesis of γ-secretase inhibitor **11** (ref. 30) was accomplished in only three steps from 1-fluoro-2-(trifluoromethoxy)benzene **1ia** with an overall 42% yield (Scheme 5). The key *meta*-arylation step afforded a 59% yield of **3ib**, which upon subsequent alkylation^29^ and Suzuki coupling^30^ led to the desired product **12**. The previous synthesis required nine steps starting from methyl 2-(3,5-dihydroxyphenyl)acetate (**13**) affording **11** in 12% overall yield.^28^

## Conclusion

In summary, we have developed the first methodology for the direct *meta*-(hetero)arylation of fluoroarenes. Our method relies on the use of CO_2_ as traceless directing group. This one-pot protocol involving lithiation/carboxylation followed by tandem arylation/decarboxylation has proven compatible with different substituents and substitution patterns in both the fluoroarene and aryl iodide coupling partners. The method can be successfully applied to other classes of aryl ethers capable of directing selective *ortho*-lithiation events. It should be noted that most of the examples here presented cannot be currently directly made from the parent arenes *via* any other method.

## Conflicts of interest

There are no conflicts to declare.

## Supplementary Material

Supplementary information

Crystal structure data
