# Supplementary material for: meta-C–H arylation of fluoroarenes via traceless directing group relay strategy
Source: Chem Sci. 2018 Jul 25;9(35):7133–7. doi: 10.1039/c8sc02417k (PMC6137440; doi:10.1039/c8sc02417k)
Supplement: Supplementary file 1 [file SC-009-C8SC02417K-s001.pdf]

Electronic Supplementary Material (ESI) for Chemical Science.
This journal is © The Royal Society of Chemistry 2018

Supporting Information

meta-C–H arylation of fluoroarenes *via* traceless directing group relay strategy

Marc Font, Andrew R. A. Spencer, and Igor Larrosa*

School of Chemistry, University of Manchester, Oxford Road, M13 9PL, Manchester (United Kingdom)

Email: igor.larrosa@manchester.ac.uk

Table of Contents

General experimental information S2

Experimental procedures and characterisation data for *m*-arylated fluoroarenes S2

Experimental procedures for the stepwise synthesis of 3-fluoro-3',4,5'-trimethyl-1,1'-biphenyl S15

Experimental procedures and characterisation data for *m*-arylated aryl alkyl ethers and acetals S17

Scope of the one-pot *m*-arylation of fluoroarenes . ……………………………………..S20

Preparation of the γ-secretase inhibitor **7** S23

Crystallographic data S25

References...S28

NMR spectral data S29

General experimental information

THF was freshly distilled over sodium and benzophenone prior to its use. Commercial solutions of *sec*-butyl lithium were titrated using N-benzylbenzamide as indicator. All other solvents and reagents were purchased from commercial suppliers and used without further purification unless otherwise stated. All air and moisture-sensitive reactions and lithiation reactions were carried out under dry argon or nitrogen atmosphere.

Column chromatography was performed on silica gel (40-63 μm) unless otherwise stated. $AgNO_3$ impregnated silica gel was prepared by absorbed a solution of $AgNO_3$ in MeCN (10% wt of $AgNO_3$ to silica) on silica. The MeCN was removed under reduced pressure on a rotary evaporator and the silica was further dried at 80 °C and < 1 mbar for 1-2 h. Thin layer chromatography (TLC) was carried out on pre-coated silica gel F_{254} plates with visualization under UV light or using an aqueous basic $KMnO_4$ solution.

Melting points (mp) are uncorrected and were obtained using a Stuart SMP11 apparatus. IR spectra were recorded using a Thermo Scientific Nicolet iS5 FTIR spectrometer and the relevant peaks are quoted in cm^{-1}. NMR data was collected on a Bruker Avance III 400 MHz or Bruker AvanceII+ 500 MHz spectrometers. Chemical shifts are given in ppm (δ) and are referenced to the residual $CDCl_3$ solvent peak at 7.26 ppm (^1H NMR) and 77.16 ppm (^{13}C NMR). Conventional one-dimensional (1D) ^1H NMR, ^{19}F NMR, $^{13}C\{^1H\}$ NMR and two-dimensional (2D) ^1H-^{13}C heteronuclear single quantum coherence (HSQC) experiments were recorded at room temperature under routine conditions. High Resolution Mass Spectra (HRMS) were performed by the School of Chemistry Mass Spectrometry Service of the University of Manchester on a Thermo Finnigan MAT95XP spectrometer.

Experimental procedures and characterisation data for *m*-arylated fluoroarenes

General procedure A: A flame-dried crimpable glass vial (CEM Microwave Technologies, 10 mL volume) was loaded with the fluoroarene (if solid), capped with a rubber septum, evacuated and filled with argon three times. The vial was loaded with 1.25 mL of a 0.4 M solution of fluoroarene in dry THF if liquid or 1.25 mL of dry THF if solid. The solution was cooled down to -78 °C and 0.36 mL of a freshly titrated solution 1.4 M of *sec*-butyl lithium in cyclohexane were added dropwise. After reacting for 30 minutes at -78 °C, CO_2 was bubbled into the reaction mixture. The resulting mixture was warmed up to room temperature and the solvents were removed under a stream of nitrogen. To the resulting white solid, $Pd(OAc)_2$ (2.2 mg, 0.01 mmol), Ag_2CO_3 (137.9 mg, 0.5 mmol), aryl iodide (0.25-1.5 mmol) and trifluoroacetic acid (100 μl) were added, the vial was capped with a Teflon lined septum and the slurry was heated at the indicated temperature for 16 hours. Upon completion of the

reaction, the mixture was diluted with diethyl ether and filtered through a plug of Celite® and concentrated *in vacuo*. Purification by column chromatography loading the crude product absorbed on silica and using the eluents specified afforded the pure *m*-arylated products.

General procedure B: A flame-dried crimpable glass vial (CEM Microwave Technologies, 10 mL volume) was loaded with the fluoroarene (if solid), capped with a rubber septum, evacuated and filled with argon three times. The vial was loaded with 1.25 mL of a 0.4 M solution of fluoroarene in dry THF if liquid or 1.25 mL of dry THF if solid. The solution was cooled down to -78 °C and 0.36 mL of a freshly titrated solution 1.4 M of *sec*-butyl lithium in cyclohexane were added dropwise. After reacting for 30 minutes at -78 °C, CO_2 was bubbled into the reaction mixture. The resulting mixture was warmed up to room temperature and the solvents were removed under a stream of nitrogen. To the resulting white solid, $Pd(OAc)_2$ (2.2 mg, 0.01 mmol), Ag_2CO_3 (137.9mg, 0.5 mmol), aryl iodide (0.25-1.5 mmol) and trifluoroacetic acid (100 μl) were added, the vial was capped with a Teflon lined septum and the slurry was heated at the indicated temperature for 16 hours. After that time, the reaction mixture was cooled down to room temperature, DMSO (2 mL) was added and the reaction was stirred at 170 °C for 3 additional hours. Upon completion of the reaction, the mixture was diluted with diethyl ether filtered through a plug of Celite®, washed with a solution of saturated ammonium chloride and concentrated *in vacuo*. Purification by column chromatography loading the crude product absorbed on silica and using the eluents specified afforded the pure *m*-arylated products.

General procedure C: A flame-dried crimpable glass vial (CEM Microwave Technologies, 10 mL volume) was loaded with the fluoroarene (if solid), capped with a rubber septum, evacuated and filled with argon three times. The vial was loaded with 1.25 mL of a 0.4 M solution of fluoroarene in dry THF if liquid or 1.25 mL of dry THF if solid. The solution was cooled down to -78 °C and 0.36 mL of a freshly titrated solution 1.4 M of *sec*-butyl lithium in cyclohexane were added dropwise. After reacting for 30 minutes at -78 °C, CO_2 was bubbled into the reaction mixture. The resulting mixture was warmed up to room temperature and the solvents were removed under a stream of nitrogen. To the resulting white solid, $Pd(OAc)_2$ (2.2 mg, 0.01 mmol), Ag_2CO_3 (137.9 mg, 0.5 mmol), aryl iodide (0.25-1.5 mmol) and trifluoroacetic acid (100 μl) were added, the vial was capped with a Teflon lined septum and the slurry was heated at the indicated temperature for 16 hours. Then a second addition of $Pd(OAc)_2$ (2.2 mg, 0.02 mmol) was made and the reaction mixture was stirred at 130 C for 11 additional hours. After that time, the reaction mixture was cooled down to room temperature, DMSO (2 mL) was added and the reaction was stirred at 170 °C for 3 additional hours. Upon completion of the reaction, the mixture was diluted with diethyl ether filtered through a plug of Celite®, washed with a solution of saturated ammonium chloride and concentrated *in vacuo*. Purification by column chromatography loading the crude product absorbed on silica and using the eluents specified afforded the pure *m*-arylated products.

3-fluoro-3', 4 ,5'-trimethyl-1,1'-biphenyl

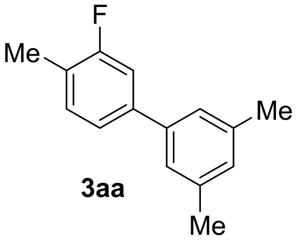

3aa

Following the general procedure A running the arylation/decarboxylation step at 130 °C, 2-fluorotoluene **1a** (0.50 mmol) was reacted with 1-iodo-3,5-dimethylbenzene **7a** (216 μl, 1.5 mmol). The crude product was purified by column chromatography (petroleum ether) to afford 3-fluoro-3',4,5'-trimethyl-1,1'-biphenyl (**3aa**) as a colourless oil (74.8 mg, 70%). **IR** (ATR), ν, cm^{-1}: 1580, 1162, 1127, 843, 817. **^{1}H NMR** (500 MHz, $CDCl_3$) δ, ppm: 7.28-7.24 (m, 2H), 7.23−7.21 (m, 1H), 7.19 (s, 2H), 7.00 (s, 1H), 2.39 (s, 6H), 2.32 (d, J_{HF} = 1.7 Hz, 3H). **^{13}C NMR** (126 MHz, $CDCl_3$) δ, ppm: 161.9 (d, J_{CF} = 244.2 Hz), 141.3 (d, J_{CF} = 7.6 Hz), 140.3 (d, J_{CF} = 2.1 Hz), 138.7, 131.9 (d, J_{CF} = 7.6 Hz), 129.5, 125.1, 123.8 (d, J_{CF} = 17.4 Hz), 122.7 (d, J_{CF} = 3.2 Hz), 113.9 (d, J_{CF} = 23.0 Hz), 21.7, 14.6 (d, J_{CF} = 3.4 Hz). **^{19}F-NMR** (471 MHz, $CDCl_3$) δ, ppm: -117.6 (m). **HRMS** (APCI, **[M-H]**$^+$) *m/z* calculated for $C_{15}H_{16}F^+$, 215.1229, found: 215.1231.

3-fluoro-4-methyl-3',5'-bis(trifluoromethyl)-1,1'-biphenyl

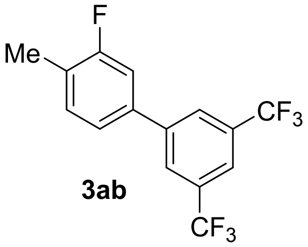

3ab

Following the general procedure A running the arylation/decarboxylation step at 130 °C, 2-fluorotoluene **1a** (0.50 mmol) was reacted with 1-iodo-3,5-bis(trifluoromethyl)benzene **7b** (271 μl, 1.5 mmol). The crude product was purified by column chromatography (petroleum ether) using small particle size silica (0.015-0.04 mm) to afford 3-3-fluoro-4-methyl-3',5'-bis(trifluoromethyl)-1,1'-biphenyl (**3ab**) as a white solid (118.0 mg, 71%). Suitable crystals of **3ab** for X-ray diffraction analysis were grown by slow evaporation from a concentrated solution of the compound in hexanes. **m.p.** = 51 °C. **IR** (ATR), ν, cm^{-1}: 1463, 1370, 1268, 1156, 1107, 894, 863, 702. **^{1}H NMR** (500 MHz, $CDCl_3$) δ, ppm: 7.98 (s, 2H), 7.86 (s, 1H), 7.34-7.25 (m, 3H), 2.35 (d, J_{HF} = 1.6 Hz, 3H). **^{13}C NMR** (126 MHz, $CDCl_3$) δ, ppm: 161.9 (d, J_{CF} = 246.4 Hz), 142.2 (d, J_{CF} = 2.0 Hz), 137.7 (d, J_{CF} = 7.7 Hz), 132.3 (q, J_{CF} = 33.3 Hz), 132.4, 127.1 (d, J_{CF} = 2.8 Hz), 125.9 (d, J_{CF} = 17.3 Hz), 123.5 (q, J_{CF} = 273.2 Hz), 122.6 (d, J_{CF} = 3.3 Hz), 121.3 (sept, J_{CF} = 3.8 Hz), 113.9 (d, J_{CF} = 23.7 Hz), 14.5 (d, J_{CF} = 3.3 Hz).

^{19}**F-NMR** (471 MHz, $CDCl_3$) δ, ppm: -62.9 (s), -116.1 (m). **HRMS** (EI, **[M]$^+$**) *m/z* calculated for $C_{15}H_9F_7^+$, 322.0587, found: 322.0577.

3'-bromo-5'-chloro-3-fluoro-4-methyl-1,1'-biphenyl

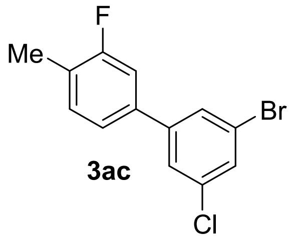

3ac

Following the general procedure B running the arylation/decarboxylation step at 130 °C, 2-fluorotoluene **1a** (0.50 mmol) was reacted with 1-bromo-3-chloro-5-iodobenzene **7c** (476 mg, 1.5 mmol). The crude product was purified by column chromatography on silica gel (petroleum ether) to afford 3'-bromo-5'-chloro-3-fluoro-4-methyl-1,1'-biphenyl (**3ac**) as white crystals (64 mg, 43%). **m.p** = 73-74°C. **IR** (ATR), ν, cm^{-1}: 1586, 1550, 1390, 1178, 856, 816, 780, 749. ^{1}**H NMR** (400 MHz, $CDCl_3$) δ, ppm: 7.58 (t, J = 1.7 Hz, 1H), 7.48 (app dt, J = 8.6 Hz, J = 1.8 Hz, 2H), 7.29 – 7.16 (m, 3H), 2.33 (d, J = 1.9 Hz, 3H).^{13}**C NMR** (101 MHz, $CDCl_3$) δ, ppm: 161.7 (d, J_{CF} = 245.6 Hz), 143.3, 138.0 (d, J_{CF}= 7.7 Hz), 135.6, 132.1, 130.3, 128.4, 126.0, 125.4 (d, J_{CF} = 17.5 Hz), 123.3, 122.4, 113.7 (d, J_{CF} = 23.5 Hz), 14.5 (d, J_{CF} = 3.4 Hz). ^{19}**F-NMR** (376 MHz, $CDCl_3$) δ, ppm: 116.52 (ddd, J = 10.5, J = 6.2, J = 2.1 Hz). **HRMS** (EI, **[M]$^+$**) *m/z* calculated for $C_{13}H_9BrClF^+$, 297.9560, found: 297.9565.

2,6-dichloro-4-(3-fluoro-4-methylphenyl)pyridine

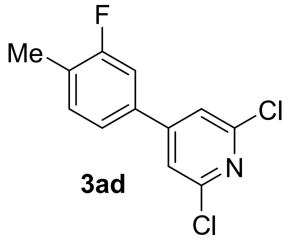

3ad

Following the general procedure B running the arylation/decarboxylation step at 130 °C, 2-fluorotoluene **1a** (0.50 mmol) was reacted with 2,6-dichloro-4-iodopyridine **7d** (205.4 mg, 0.75 mmol). The crude solution of the product was basified to pH 14 using a 2M solution of KOH prior to its wash with ammonium chloride. The crude product was purified by column chromatography on silica (hexanes:EtOAc 100:0 to 90:10) to afford methyl 2,6-dichloro-4-(3-fluoro-4-methylphenyl)pyridine (**3ad**) as a white solid (88.0 mg, 69%). **m.p.** = 117-119 °C. **IR** (ATR), ν, cm^{-1}: 1579, 1523, 1363, 1147, 1168, 853, 807, 769. ^{1}**H NMR** (400 MHz, $CDCl_3$) δ, ppm: 7.43 (s, 2H), 7.34-7.23 (m, 3H), 2.34 (d, J_{HF} = 1.8 Hz, 3H). ^{13}**C NMR** (101 MHz, $CDCl_3$) δ, ppm: 161.8 (d, J_{CF} = 247.6

Hz), 152.7 (d, J_{CF} = 2.2 Hz), 151.3, 135.1 (d, J_{CF} = 7.8 Hz), 132.6 (d, J_{CF} = 5.5 Hz), 127.6 (d, J_{CF} = 17.3 Hz), 122.5 (d, J_{CF} = 3.4 Hz), 120.6, 113.7 (d, J_{CF} = 24.0 Hz), 14.6 (d, J_{CF} = 3.3 Hz). **^{19}F-NMR** (376 MHz, $CDCl_3$) δ, ppm: -115.4 (m). **HRMS** (APCI, **[M-H]**$^+$) *m/z* calculated for $C_{12}H_9NCl_2F^+$, 256.0091, found: 256.0092.

3'-bromo-3-fluoro-4-methyl-1,1'-biphenyl

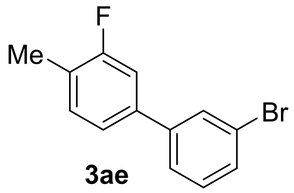

3ae

Following the general procedure B running the arylation/decarboxylation step at 130 °C, 2-fluorotoluene **1a** (0.50 mmol) was reacted with 3-bromoiodobenzene **7e** (96 µl, 0.75 mmol). The crude product was purified by column chromatography (hexanes) using small particle size silica (0.015-0.04 mm) to afford 3'-bromo-3-fluoro-4-methyl-1,1'-biphenyl (**3ae**) as a colourless oil (63.5 mg, 48%). **IR** (ATR), ν, cm^{-1}: 1513, 1022, 932, 717. **^{1}H NMR** (400 MHz, $CDCl_3$) δ, ppm: 7.72 (t, J = 1.8 Hz, 1H), 7.50-7.48 (dd, J = 7.8 Hz, J = 1.6 Hz, 2H), 7.35-7.21 (m, 3H), 2.33 (d, J_{HF} = 1.9 Hz, 3H). **^{13}C NMR** (101 MHz, $CDCl_3$) δ, ppm: 161.7 (d, J_{CF} = 246.0 Hz), 142.2 (d, J_{CF} = 2.1 Hz), 139.3 (d, J_{CF} = 7.7 Hz), 132.0 (d, J_{CF} = 5.6 Hz), 130.6, 130.5, 130.1, 125.6, 124.6 (d, J_{CF} = 17.3 Hz), 123.1, 122.5 (d, J_{CF} = 3.3 Hz), 113.7 (d, J_{CF} = 23.4 Hz), 14.5 (d, J_{CF} = 3.4 Hz). **^{19}F-NMR** (376 MHz, $CDCl_3$) δ, ppm: -117.0 (m). **HRMS** (APCI, **[M]**$^+$) *m/z* calculated for $C_{13}H_{10}BrF^+$, 263.9944, found: 263.9943.

2-(3-fluoro-4-methylphenyl)-6-(trifluoromethyl)pyridine

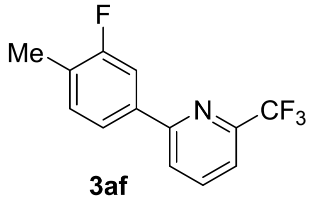

3af

Following the general procedure A running the arylation/decarboxylation step at 130 °C, 2-fluorotoluene **1a** (0.50 mmol) was reacted with 2-iodo-6-(trifluoromethyl)pyridine **7f** (71.8 mg, 0.25 mmol) using 1.1 mg (0.005 mmol) of $Pd(OAc)_2$ and 69 mg (0.25 mmol) of Ag_2CO_3. The crude solution of the product was basified to pH 14 using a 2M solution of KOH prior to its wash with ammonium chloride. The crude product was purified by column chromatography on silica (hexanes:EtOAc 100:0 to 90:10) to afford methyl 2-(3-fluoro-4-methylphenyl)-6-(trifluoromethyl)pyridine (**3af**) as a white solid (26.9 mg, 42%). **m.p.** = 98-99 °C. **IR** (ATR), ν, cm^{-1}: 1496, 1457, 1227, 1183, 1144, 1031, 847, 810, 708. **^{1}H NMR** (400 MHz, $CDCl_3$) δ, ppm: 7.93-7.86 (m, 2H), 7.78-7.72 (m, 2H), 7.60 (dd, J_{HH} = 7.4 Hz, J_{HH} = 0.9 Hz, 1H), 7.29 (app t, J = 7.6 Hz, 1H),

2.34 (d, J_{HF} = 1.6 Hz, 3H). **^{13}C NMR** (101 MHz, $CDCl_3$) δ, ppm: 161.9 (d, J_{CF} = 245.7 Hz), 156.7 (d, J_{CF} = 2.5 Hz), 148.3 (q, J_{CF} = 34.8 Hz), 138.3, 137.5 (d, J_{CF} = 7.7 Hz), 132.0 (d, J_{CF} = 5.3 Hz), 126.9 (d, J_{CF} = 17.5 Hz), 122.6 (d, J_{CF} = 0.6 Hz), 122.4 (d, J_{CF} = 3.3 Hz), 121.6 (q, J_{CF} = 275.4 Hz), 118.8 (q, J_{CF} = 2.8 Hz), 113.8 (d, J_{CF} = 24.2 Hz), 14.7 (d, J_{CF} = 3.4 Hz). **^{19}F-NMR** (376 MHz, $CDCl_3$) δ, ppm: -68.2 (s), -116.8 (m). **HRMS** (APCI, **[M-H]$^+$**) *m/z* calculated for $C_{13}H_{10}NF_4^+$, 256.0744, found: 256.0741.

methyl 3'-fluoro-4'-methyl-[1,1'-biphenyl]-3-carboxylate

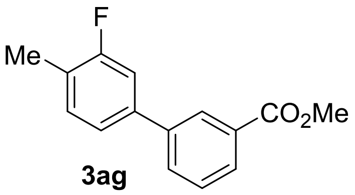

3ag

Following the general procedure A running the arylation/decarboxylation step at 130 °C, 2-fluorotoluene **1a** (0.50 mmol) was reacted with methyl 3-iodobenzoate **7g** (262 mg, 1 mmol). The crude product was purified by column chromatography on silica (hexanes:EtOAc 99:1) to afford methyl methyl 3'-fluoro-4'-methyl-[1,1'-biphenyl]-3-carboxylate (**3ag**) as a colourless oil (53.7 mg, 44%). **IR** (ATR), ν, cm^{-1}: 1722, 1309, 1253, 1172, 1109, 750, 730. **^{1}H NMR** (400 MHz, $CDCl_3$) δ, ppm: 8.24 (t, J = 1.6 Hz, 1H), 8.01 (dt, J = 7.8 Hz, J = 1.4 Hz, 1H), 7.74 (ddd, J = 7.8 Hz, J = 1.9 Hz, J = 1.2 Hz, 1H), 7.50 (t, J = 7.7 Hz, 1H), 7.31–7.23 (m , 3H), 3.95 (s, 3H), 2.32 (d, J_{HF} = 1.7 Hz, 3H). **^{13}C NMR** (101 MHz, $CDCl_3$) δ, ppm: 167.1, 161.8 (d, J_{CF} = 245.7 Hz), 140.3 (d, J_{CF} = 2.0 Hz), 139.7 (d, J_{CF} = 7.7 Hz), 132.0 (d, J_{CF} = 5.6 Hz), 131.3, 130.9, 129.0, 128.7, 128.1, 124.4 (d, J_{CF} = 17.3 Hz), 122.5 (d, $J_{C\text{-}F}$ = 3.2 Hz), 113.7 (d, J_{CF} = 23.3 Hz), 52.3, 14.4 (d, J_{CF} = 3.4 Hz). **^{19}F-NMR** (376 MHz, $CDCl_3$) δ, ppm: -117.1 (m). **HRMS** (APCI, **[M-H]$^+$**) *m/z* calculated for $C_{15}H_{14}O_2F^+$, 245.0972, found: 245.0971.

3,4'-difluoro-4-methyl-1,1'-biphenyl

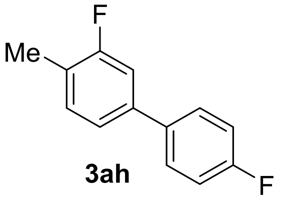

3ah

Following the general procedure B running the arylation/decarboxylation step at 130 °C, 2-fluorotoluene **1a** (0.50 mmol) was reacted with 4-fluoroiodobenzene **7h** (116 µl, 1 mmol). The crude product was purified by column chromatography (petroleum ether) using small particle size silica (0.015-0.04 mm) to afford 3,4'-difluoro-4-methyl-1,1'-biphenyl (**3ah**) as a colourless oil (42.7 mg, 42%). **IR** (ATR), ν, cm^{-1}: 1499, 907, 725. **^{1}H NMR** (400 MHz, $CDCl_3$) δ, ppm: 7.54-7.19 (m, 2H),

7.24-7.18 (m, 3H), 7.15-7.09 (m, 2H), 2.31 (d, J_{HF} = 1.8 Hz, 3H). **^{13}C NMR** (101 MHz, $CDCl_3$) δ, ppm: 162.7 (d, J_{CF} = 247.6 Hz), 161.7 (d, J_{CF} = 245.5 Hz), 139.9 (d, J_{CF} = 7.8 Hz), 136.3-136.2 (dd, J_{CF} = 2.3 Hz, J_{CF} = 0.8 Hz), 131.9 (d, J_{CF} = 5.6 Hz), 128.6 (d, J_{CF} = 8.1 Hz), 123.8 (d, J_{CF} = 17.4 Hz), 122.3 (d, J_{CF} = 3.2 Hz), 115.8 (d, J_{CF} = 21.1 Hz), 113.6 (d, J_{CF} = 23.2 Hz), 14.4 (d, J_{CF} = 3.4 Hz). **^{19}F-NMR** (376 MHz, $CDCl_3$) δ, ppm: -115.3 (m), -117.3 (m). **HRMS** (EI, **[M]$^+$**) *m/z* calculated for $C_{13}H_{10}F_2^+$, 204.0745, found: 204.0737.

3-fluoro-4,4'-dimethyl-1,1'-biphenyl

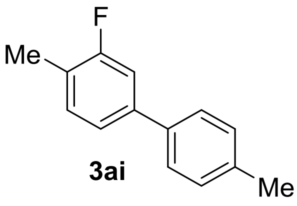

3ai

Following the general procedure B running the arylation/decarboxylation step at 130 °C, 2-fluorotoluene **1a** (0.50 mmol) was reacted with 4-iodotoluene **7i** (218 mg, 1 mmol). The crude product was purified by column chromatography on silica (petroleum ether) to afford 3-fluoro-4,4'-dimethyl-1,1'-bipheny (**3ai**) as a white solid (41.5 mg, 41%). **m.p.** = 78-79 °C. **IR**: 1561, 1486, 1125, 889, 876, 805. **^{1}H NMR** (500 MHz, $CDCl_3$) δ, ppm: 7.49 (d, J = 8.0 Hz, 2H), 7.30-7.23 (m, 5H), 2.43 (s, 3H), 2.34 (d, J = 1.7 Hz, 3H). **^{13}C NMR** (126 MHz, $CDCl_3$) δ, ppm: 161.7 (d, J_{CF} = 244.7 Hz), 140.9 (d, J_{CF} = 7.7 Hz), 137.5, 137.2 (d, J_{CF} = 2.0 Hz), 131.8 (d, J_{CF} = 5.7 Hz), 129.7, 126.9, 123.5 (d, J_{CF} = 17.4 Hz), 122.3 (d, J_{CF} = 3.1 Hz), 113.5 (d, J_{CF} = 22.9 Hz), 21.2, 14.4 (d, J_{CF} = 3.4 Hz). **^{19}F-NMR** (471 MHz, $CDCl_3$) δ, ppm: -117.6 (m). **HRMS** (APCI, **[M]$^+$**) *m/z* calculated for $C_{14}H_{13}F^+$, 200.0996, found: 200.0995.

3-fluoro-4-methyl-4'-nitro-1,1'-biphenyl

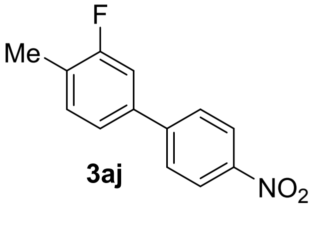

3aj

Following the general procedure B running the arylation/decarboxylation step at 150 °C, 2-fluorotoluene **1a** (0.50 mmol) was reacted with 1-iodo-4-nitrobenzene **7j** (190 mg, 0.75 mmol). The crude product was purified by column chromatography (hexanes:EtOAc 97:3) to afford 3-fluoro-4-methyl-4'-nitro-1,1'-biphenyl (**3aj**) as a white solid (71.5 mg, 62%). **m.p** = 100-102 °C. **IR** (ATR), ν, cm^{-1}: 2927, 1583, 1575, 1512, 1486, 1339, 1334, 1170, 881, 805, 747. **^{1}H NMR** (500 MHz, $CDCl_3$) δ, ppm: 8.30-8.28 (m, 2H), 7.72-7.69 (m, 2H), 7.32-7.27 (m, 3H), 2.34 (d, J_{HF} = 1.9 Hz, 3H). **^{13}C NMR** (126 MHz, $CDCl_3$) δ, ppm: 161.8 (d, J_{CF} = 246.3 Hz), 147.4, 146.4 (d, J_{CF} = 2.1 Hz), 138.3 (d, J_{CF} =

7.7 Hz), 132.3 (d, J_{CF} = 5.6 Hz), 127.7, 126.0 (d, J_{CF} = 17.3 Hz), 124.3, 122.8 (d, J_{CF} = 3.3 Hz), 114.0 (d, J_{CF} = 23.6 Hz), 14.5 (d, J_{CF} = 3.4 Hz). **^{19}F-NMR** (471 MHz, $CDCl_3$) δ, ppm: -116.3 (m). **HRMS** (EI, **[M]$^+$**) *m/z* calculated for $C_{13}H_{10}O_2NF^+$, 231.0690, found: 231.0693.

2,3'-difluoro-4'-methyl-1,1'-biphenyl

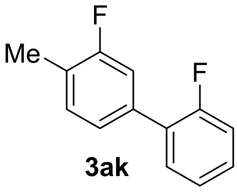

3ak

Following the general procedure B running the arylation/decarboxylation step at 130 °C, 2-fluorotoluene **1a** (0.50 mmol) was reacted with 1-fluoro-2-iodobenzene **7k** (175 µl, 1.5 mmol). The crude product was purified by column chromatography on silica gel (petroleum ether) to afford 2,3'-difluoro-4'-methyl-1,1'-biphenyl (**3ak**) as a colourless oil (35 mg, 34%). **IR** (ATR), ν, cm^{-1}:1482, 1406, 1256, 1217, 1127, 900, 811, 753. **^{1}H NMR** (400 MHz, $CDCl_3$) δ 7.44 (app td, J = 7.8, 1.8 Hz, 1H), 7.34 (app tdd, J = 7.3, J = 4.9, J = 1.8 Hz, 1H), 7.28 – 7.15 (m, 5H), 2.35 (d, J = 1.8 Hz, 3H). **^{13}C NMR** (126 MHz, $CDCl_3$) δ 161.6 (d, J_{CF} =184.4 Hz), 159.6 (d, J_{CF} =188.4 Hz), 135.3 (d, J_{CF} = 7.9 Hz), 131.5 (d, J_{CF} = 5.5 Hz), 130.6 (d, J_{CF} = 3.1 Hz), 129.3 (d, J_{CF} = 8.3 Hz), 128.1 (dd, J_{CF} = 13.1 Hz, J_{CF} = 2.2 Hz), 124.5 (d, J_{CF} = 3.9 Hz), 124.5 (d, J_{CF} = 3.3 Hz) 124.4 (d, J_{CF} = 23.9 Hz), 116.3 (d, J_{CF} = 22.6 Hz), 115.7 (dd, J_{CF} = 23.3 Hz, J_{CF} = 3.3 Hz), 14.5 (d, J_{CF} = 3.5 Hz). **^{19}F NMR** (376 MHz, $CDCl_3$) δ -117.48 – -117.63 (m), -117.79 – -117.91 (m). **HRMS** (EI, **[M]$^+$**) *m/z* calculated for $C_{13}H_{10}F_2^+$, 204.0751, found: 204.0749.

3'-fluoro-3,5-dimethyl-1,1'-biphenyl

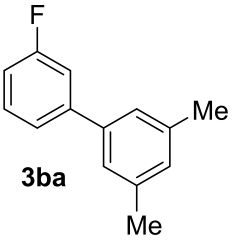

3ba

Following the general procedure B running the arylation/decarboxylation step at 130 °C, fluorobenzene **1b** (0.50 mmol) was reacted with 1-iodo-3,5-dimethylbenzene **7a** (216 µl, 1.5 mmol). The crude product was purified by column chromatography on silica (hexanes) to afford 3'-fluoro-3,5-dimethyl-1,1'-biphenyl (**3ba**) as a colourless oil (63.9 mg, 64%). Spectroscopic data matched the previously reported.[1] **^{1}H NMR** (400 MHz, $CDCl_3$) δ, ppm: 7.43-7.38 (m, 2H), 7.33-7.30 (m, 1H), 7.24 (s, 2H), 7.08-7.03 (m, 2H), 2.42 (s, 6H). **^{13}C NMR** (101 MHz, $CDCl_3$) δ, ppm: 163.3 (d, J_{CF} = 246.2 Hz), 143.9 (d, J_{CF} = 7.7 Hz), 140.1 (d, J_{CF} = 2.1 Hz), 138.5, 130.2 = (d, J_{CF} = 8.4 Hz), 129.6, 125.1,

122.9 (d, J_{CF} = 2.7 Hz), 114.1 (d, J_{CF} = 18.8 Hz), 113.9 (d, J_{CF} = 18.1 Hz), 21.5. **^{19}F-NMR** (376 MHz, $CDCl_3$) δ, ppm: -113.3 (m).

2,5-difluoro-3',5'-dimethyl-1,1'-biphenyl

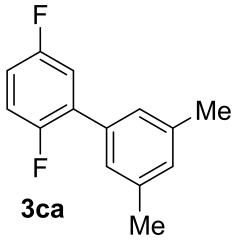

3ca

Following the general procedure B running the arylation/decarboxylation step at 130 °C, 1,4-difluorobenzene **1c** (0.50 mmol) was reacted with 1-iodo-3,5-dimethylbenzene **7a** (216 µl, 1.5 mmol). The crude product was purified by column chromatography (petroleum ether) using small particle size silica (0.015-0.04 mm) to afford 2,5-difluoro-3',5'-dimethyl-1,1'-biphenyl (**3ca**) as a colourless oil (75.4 mg, 69%). Spectroscopic data matched the previously reported.[2] **^{1}H NMR** (500 MHz, $CDCl_3$) δ, ppm: 7.18 (s, 2H), 7.17−7.09 (m, 2H), 7.07 (s, 1H), 7.02-6.97 (m, 1H), 2.41 (s, 6H). **^{13}C NMR** (126 MHz, $CDCl_3$) δ, ppm: 158.8 (dd, J_{CF} = 242.4 Hz, J_{CF} = 2.4 Hz), 155.9 (dd, J_{CF} = 243.8 Hz, J_{CF} = 2.4 Hz), 138.2, 134.8, 130.8 (dd, J_{CF} = 16.1 Hz, J_{CF} = 7.9 Hz), 130.0, 126.8 (d, J_{CF} = 3.0 Hz), 117.3-116.9 (m, 2C), 115.0 (dd, J_{CF} = 24.1 Hz, J_{CF} = 8.6 Hz), 21.4. **^{19}F-NMR** (471 MHz, $CDCl_3$) δ, ppm: -119.3 (m), -123.8 (m).

3-fluoro-3',5,5'-trimethyl-1,1'-biphenyl

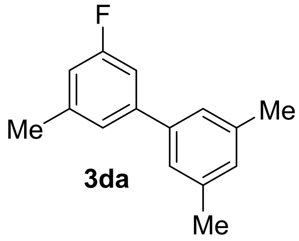

3da

Following the general procedure B running the arylation/decarboxylation step at 130 °C, 3-fluorotoluene **1d** (0.50 mmol) was reacted with 1-iodo-3,5-dimethylbenzene **7a** (37 µl, 0.25 mmol) using 1.1 mg (0.005 mmol) of $Pd(OAc)_2$ and 69 mg (0.25 mmol) of Ag_2CO_3. The crude product was purified by column chromatography (petroleum ether) using small particle size silica (0.015-0.04 mm) to afford 3-fluoro-3',5,5'-trimethyl-1,1'-biphenyl (**3da**) as a white solid (32.6 mg, 61%). **m.p.** = 32-33 °C. **IR** (ATR), ν, cm^{-1}: 1615, 1584, 1259, 1134, 836, 734. **^{1}H NMR** (400 MHz, $CDCl_3$) δ, ppm: 7.20-7.19 (m, 3H), 7.10 (d, J_{HF} = 10.0 Hz, 1H), 7.03 (s, 1H), 6.86 (d, J_{HF} = 9.4 Hz, 1H), 2.43 (s, 3H), 2.40 (s, 6H). **^{13}C NMR** (101 MHz, $CDCl_3$) δ, ppm: 163.2 (d, J_{CF} = 245.4 Hz), 143.5 (d, J_{CF} = 8.3 Hz), 140.5 (d, J_{CF} = 8.3 Hz), 140.3 (d, J_{CF} = 2.3 Hz), 138.5, 129.5, 125.1, 123.7 (d, J_{CF} = 2.3 Hz), 114.6 (d, J_{CF} =

21.2 Hz), 111.2 (d, J_{CF} = 22.1 Hz), 21.6 (d, J_{CF} = 1.9 Hz), 21.5. **^{19}F-NMR** (376 MHz, $CDCl_3$) δ, ppm: -114.5 (t, J_{FH} = 9.7 Hz). **HRMS** (APCI, **[M]$^+$**) *m/z* calculated for $C_{15}H_{15}F^+$, 214.1152, found: 214.1152.

3,4-difluoro-3',5'-dimethyl-1,1'-biphenyl

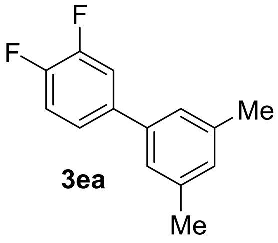

3ea

Following the general procedure B running the arylation/decarboxylation step at 130 °C, 1,2-difluorobenzene **1e** (0.50 mmol) was reacted with 1-iodo-3,5-dimethylbenzene **7a** (216 µl, 1.5 mmol). The crude product was purified by column chromatography (petroleum ether) using small particle size silica (0.015-0.04 mm) to afford 3,4-difluoro-3',5'-dimethyl-1,1'-biphenyl (**3ea**) as a colourless oil (75.6 mg, 69%). **IR** (ATR), ν, cm^{-1}: 1499, 903, 721. **^{1}H NMR** (400 MHz, $CDCl_3$) δ, ppm: 7.37 (ddd, J = 11.6 Hz, J = 7.6 Hz, J = 2.2 Hz, 1H), 7.30−7.27 (m, 1H), 7.23-7.17 (m, 1H), 7.14 (s, 2H), 7.03 (s, 1H), 2.39 (s, 6H). **^{13}C NMR** (101 MHz, $CDCl_3$) δ, ppm: 150.6 (dd, J_{CF} = 248.5 Hz, J_{CF} = 12.8 Hz), 149.9 (dd, J_{CF} = 248.9 Hz, J_{CF} = 12.9 Hz), 139.3, 138.7 (dd, J_{CF} = 5.9 Hz, J_{CF} = 3.8 Hz), 138.6, 129.6, 125.0, 123.1 (dd, J_{CF} = 6.2 Hz, J_{CF} = 3.4 Hz), 117.5 (d, J_{CF} = 17.3 Hz), 116.1 (d, J_{CF} = 17.7 Hz), 21.5. **^{19}F-NMR** (376 MHz, $CDCl_3$) δ, ppm: -137.9 (m), -140.7 (m). **HRMS** (APCI, **[M]$^+$**) *m/z* calculated for $C_{14}H_{12}F_2^+$, 218.0902, found: 218.0901.

3-fluoro-4-methoxy-3',5'-dimethyl-1,1'-biphenyl

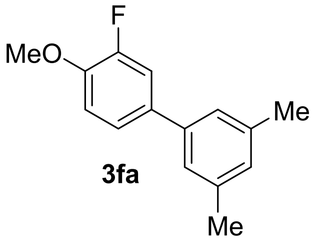

3fa

Following the general procedure B running the arylation/decarboxylation step at 130 °C, 2-fluoroanisole **1f** (0.50 mmol) was reacted with 1-iodo-3,5-dimethylbenzene **7a** (216 µl, 1.5 mmol). The crude product was purified by column chromatography on silica (hexanes:EtOAc 98:2) to afford 3-fluoro-4-methoxy-3',5'-dimethyl-1,1'-biphenyl (**3fa**) as a colourless oil (84.0 mg, 73%). **IR** (ATR), ν, cm^{-1}: 1516, 1272, 1029, 908, 724. **^{1}H NMR** (500 MHz, $CDCl_3$) δ, ppm: 7.34-7.28 (m, 2H), 7.15 (s, 2H), 7.03-6.98 (m, 1H), 6.98 (s, 1H), 3.93 (s, 3H), 2.38 (s, 6H). **^{13}C NMR** (126 MHz, $CDCl_3$) δ, ppm: 152.6 (d, J_{CF} = 245.1 Hz), 147.0 (d, J_{CF} = 10.8 Hz), 139.8 (d, J_{CF} = 1.6 Hz), 138.5, 134.9 (d, J_{CF} = 6.5 Hz), 129.0, 124.8, 122.7 (d, J_{CF} = 3.4 Hz), 114.9 (d, J_{CF} = 18.7 Hz), 113.7 (d, J_{CF} = 2.1 Hz), 56.5, 21.5.

^{19}**F-NMR** (471 MHz, $CDCl_3$) δ, ppm: -135.3 (m). **HRMS** (APCI, **[M-H]**$^+$) *m/z* calculated for $C_{15}H_{16}OF^+$, 231.1180, found: 231.1180.

3-fluoro-3',5'-dimethyl-4-(trifluoromethyl)-1,1'-biphenyl

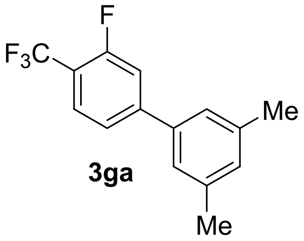

3ga

Following the general procedure B running the arylation/decarboxylation step at 130 °C, 2-fluorobenzotrifluoride **1g** (0.50 mmol) was reacted with 1-iodo-3,5-dimethylbenzene **7a** (216 µl, 1.5 mmol). The crude product was purified by column chromatography on 10% $AgNO_3$-impregnated silica (petroleum ether) to afford 3-fluoro-3',5'-dimethyl-4-(trifluoromethyl)-1,1'-biphenyl (**3ga**) as a colourless oil (56.5 mg, 42%). **IR** (ATR), ν, cm^{-1}: 1633, 1317, 1130, 903, 729. ^{1}**H NMR** (400 MHz, $CDCl_3$) δ, ppm: 7.64 (app. t, J = 7.80 Hz, 1H), 7.45-7.38 (m, 2H), 7.20 (s, 2H), 7.08 (s, 1H), 2.40 (s, 6H). ^{13}**C NMR** (101 MHz, $CDCl_3$) δ, ppm: 160.1 (dq, J_{CF} = 256.3 Hz, J_{CF} = 2.1 Hz), 147.9 (d, J_{CF} = 8.1 Hz), 138.9, 138.6 (d, J_{CF} = 1.7 Hz), 130.6, 127.5 (qd, J_{CF} = 4.5 Hz, J_{CF} = 2.0 Hz), 125.2, 123.0 (qd, J_{CF} = 273.0 Hz, J_{CF} = 0.7 Hz), 122.7 (d, J_{CF} = 3.3 Hz), 116.8 (qd, J_{CF} = 33.2 Hz, J_{CF} = 12.7 Hz), 115.4 (d, J_{CF} = 21.3 Hz), 21.5. ^{19}**F-NMR** (376 MHz, $CDCl_3$) δ, ppm: -61.03 (d, J = 12.3 Hz), -114.5 (m). **HRMS** (EI, **[M]**$^+$) *m/z* calculated for $C_{15}H_{12}F_4^+$, 268.0870, found: 268.0879.

4-chloro-3-fluoro-3',5'-dimethyl-1,1'-biphenyl

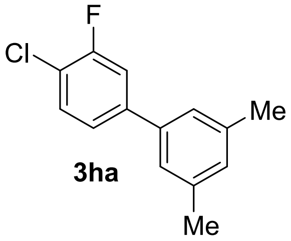

3ha

Following the general procedure C running the arylation/decarboxylation step at 130 °C, 1-chloro-2-fluorobenzene **1h** (0.50 mmol) was reacted with 1-iodo-3,5-dimethylbenzene **7a** (216 µl, 1.5 mmol). The crude product was purified by column chromatography on silica (petroleum ether) to afford 4-chloro-3-fluoro-3',5'-dimethyl-1,1'-biphenyl (**3ha**) as a colourless oil (73.1 mg, 62%). **IR** (ATR), ν, cm^{-1}: 1555, 1473, 1175, 906, 723. ^{1}**H NMR** (500 MHz, $CDCl_3$) δ, ppm: 7.43 (app. t, 1H, J = 8.0 Hz), 7.37 (dd, 1H, J = 10.4 Hz, J = 1.9 Hz), 7.31(dd, 1H, J = 8.3 Hz, J = 1.6 Hz), 7.17 (s, 2H), 7.05 (s, 1H), 2.40 (s, 6H). ^{13}**C NMR** (126 MHz, $CDCl_3$) δ, ppm: 158.4 (d, J_{CF} = 248.6 Hz), 142.2 (d, J_{CF} = 6.9 Hz), 139.0 (d, J_{CF} = 1.8 Hz), 138.7, 130.8, 129.9, 124.9, 123.5 (d, J_{CF} = 3.4 Hz), 119.7 (d, J_{CF} = 17.8 Hz),

115.2 (d, J_{CF} = 21.5 Hz), 21.5. **^{19}F-NMR** (471 MHz, $CDCl_3$) δ, ppm: -115.5 (dd, J_{FH} = 10.4 Hz, J_{FH} = 7.8 Hz). **HRMS** (EI, **[M]**$^+$) *m/z* calculated for $C_{14}H_{12}ClF^+$, 234.0606, found: 234.0606.

3-fluoro-3',5'-dimethyl-4-(trifluoromethoxy)-1,1'-biphenyl

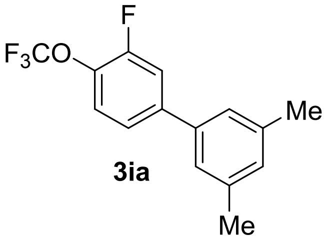

3ia

Following the general procedure B running the arylation/decarboxylation step at 130 °C, 2-(trifluoromethoxy)fluorobenzene **1i** (0.50 mmol) was reacted with 1-iodo-3,5-dimethylbenzene **7a** (216 µl, 1.5 mmol). The crude product was purified by column chromatography on silica (petroleum ether) to afford 3-fluoro-3',5'-dimethyl-4-(trifluoromethoxy)-1,1'-biphenyl (**3ia**) as a colourless oil (97.3 mg, 71%). **IR** (ATR), ν, cm^{-1}: 1594, 1510, 1265, 1210, 906, 734. **^{1}H NMR** (400 MHz, $CDCl_3$) δ, ppm: 7.42-7.39 (m, 1H), 7.35-7.34 (m, 2H), 7.16 (s, 2H), 7.05 (s, 1H), 2.40 (s, 6H). **^{13}C NMR** (101 MHz, $CDCl_3$) δ, ppm: 154.7 (d, J_{CF} = 253.1 Hz), 142.4 (d, J_{CF} = 6.8 Hz), 138.9 (d, J_{CF} = 1.6 Hz), 138.8, 135.6 (dq, J_{CF} = 12.7 Hz, J_{CF} = 1.9 Hz), 130.0, 125.1, 123.9, 123.2 (d, J_{CF} = 3.5 Hz), 120.7 (q, J_{CF} = 259.7 Hz), 116.0 (d, J_{CF} = 19.2 Hz), 21.5. **^{19}F-NMR** (376 MHz, $CDCl_3$) δ, ppm: -58.8 (d, J_{FF} = 4.8 Hz), -128.8 (m). **HRMS** (APCI, **[M-H]**$^+$) *m/z* calculated for $C_{15}H_{13}OF_4^+$, 285.0897, found: 285.0897.

2'-fluoro-3'',5''-dimethyl-1,1':4',1''-terphenyl

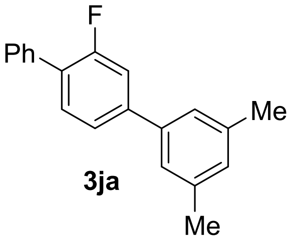

3ja

Following the general procedure B running the arylation/decarboxylation step at 130 °C, 1,2-difluorobenzene **1j** (89.7 mg, 0.50 mmol) was reacted with 1-iodo-3,5-dimethylbenzene **7a** (113 µl, 0.75 mmol). The crude product was purified by column chromatography (petroleum ether) using small particle size silica (0.015-0.04 mm) to afford 2'-fluoro-3'',5''-dimethyl-1,1':4',1''-terphenyl (**3ja**) as a colourless oil (76.1 mg, 55%). **IR** (ATR), ν, cm^{-1}: 2919, 1602, 1466, 1397, 824, 766. **^{1}H NMR** (500 MHz, $CDCl_3$) δ, ppm: 7.63 (d, J = 8.0 Hz, 2H), 7.54–7.46 (m, 4H), 7.43-7.40 (m, 2H), 7.27 (s, 2H), 7.06 (s, 1H), 2.43 (s, 6H). **^{13}C NMR** (126 MHz, $CDCl_3$) δ, ppm: 160.1 (d, J_{CF} = 248.1 Hz), 142.7 (d, J_{CF} = 8.0 Hz), 139.6 (d, J_{CF} = 1.7 Hz), 138.6, 131.0 (d, J_{CF} = 4.1 Hz), 129.7, 129.1 (d, J_{CF} = 2.9 Hz),

128.6, 127.8, 127.6 (d, J_{CF} = 13.6 Hz), 125.0, 123.1 (d, J_{CF} = 3.1 Hz), 114.7 (d, J_{CF} = 23.7 Hz), 21.5. **^{19}F-NMR** (471 MHz, $CDCl_3$) δ, ppm: -118.1 (m). **HRMS** (EI, **[M]**$^+$) *m/z* calculated for $C_{20}H_{17}F^+$, 276.1309, found: 276.1311.

3-(3,5-dimethylphenyl)-1-fluoronaphthalene

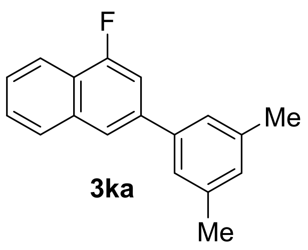

Following the general procedure A running the arylation/decarboxylation step at 130 °C, 1-fluoronaphthalene **1k** (0.50 mmol) was reacted with 1-iodo-3,5-dimethylbenzene **7a** (216 µl, 1.5 mmol). The crude product was purified by column chromatography on 10% $AgNO_3$-impregnated silica (hexanes:EtOAc 99.5:0.5) to afford 3-(3,5-dimethylphenyl)-1-fluoronaphthalene (**3ka**) as a white solid (53.8 mg, 43%). **m.p**. = 60-62 °C. **IR** (ATR), ν, cm^{-1}: 1598, 1048, 839, 748. **^{1}H NMR** (500 MHz, $CDCl_3$) δ, ppm: 8.12 (d, J = 8.0 Hz, 1H), 7.91 (d, J = 7.5 Hz, 1H), 7.84 (s, 1H), 7.57-7.52 (m, 2H), 7.45 (dd, J = 11.9 Hz, J = 0.6 Hz, 1H), 7.33 (s, 2H), 7.06 (s, 1H), 2.43 (s, 6H). **^{13}C NMR** (126 MHz, $CDCl_3$) δ, ppm: 159.1 (d, J_{CF} = 251.7 Hz), 140.3 (d, J_{CF} = 2.1 Hz), 139.3 (d, J_{CF} = 7.9 Hz), 138.6, 135.1 (d, J_{CF} = 5.3 Hz), 129.6, 128.0 (d, J_{CF} = 3.3 Hz), 127.3, 126.2 (d, J_{CF} = 1.4 Hz), 125.3, 122.9 (d, J_{CF} = 16.9 Hz), 121.4 (d, J_{CF} = 3.5 Hz), 120.6 (d, J_{CF} = 4.7 Hz), 109.4 (d, J_{CF} = 20.7 Hz), 21.6. **^{19}F-NMR** (471 MHz, $CDCl_3$) δ, ppm: -123.4 (dd, J_{FH} = 11.9 Hz, J_{FH} = 0.8 Hz). **HRMS** (APCI, **[M]**$^+$) *m/z* calculated for $C_{18}H_{15}F^+$, 250.1152, found: 250.1151.

Experimental procedures for the stepwise synthesis of 3-fluoro-3', 4 ,5'-trimethyl-1,1'-biphenyl

Lithiation/carboxylation of 2-fluorotoluene

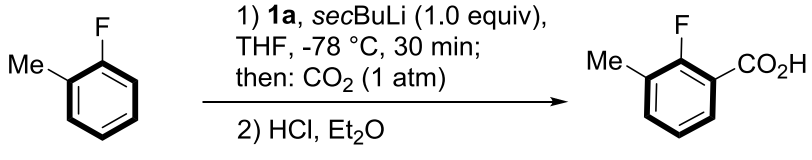

Scheme 1. Lithiation/carboxylation of 2-fluorotoluene. Yield of pure isolated product.

2-fluoro-3-methylbenzoic acid

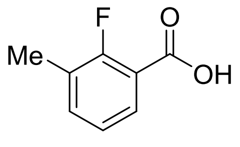

2a

A flame-dried crimpable glass vial (CEM Microwave Technologies, 10 mL volume) was loaded with 1.25 mL of a 0.4 M solution of fluoroarene in dry THF. The solution was cooled down to -78 °C and 0.36 mL of a freshly titrated solution 1.4 M of *sec*-butyl lithium in cyclohexane were added dropwise. After reacting for 30 minutes at -78 °C, CO_2 was bubbled into the reaction mixture. 0.1 mL of a 2M solution of HCl in Et_2O were added to the mixture and it was allowed to warm at room temperature. The crude product was filtered through a plug of Celite®, concentrated *in vacuo* and purified by column chromatography on silica (hexanes:EtOAc:AcOH 70:28:2) to afford 2-fluoro-3-methylbenzoic acid (**2a**) as a white solid (70.1 mg, 91%). Spectroscopic data matched that of the commercial sample. **^{1}H NMR** (400 MHz, d_6-acetone), δ, ppm: 7.76 (td, *J* = 8.0 Hz, *J* = 1.8 Hz, 1H), 7.51 (t, *J* = 7.3 Hz, 1H), 7.18 (t, *J* = 7.6 Hz, 1H), 2.30 (d, *J* = 2.5 Hz, 3H). **^{19}F NMR** (376 MHz, d_6-acetone), δ, ppm: -115.30 (m).

Palladium-catalyzed arylation/decarboxylation of 2-fluoro-3-methylbenzoic acid

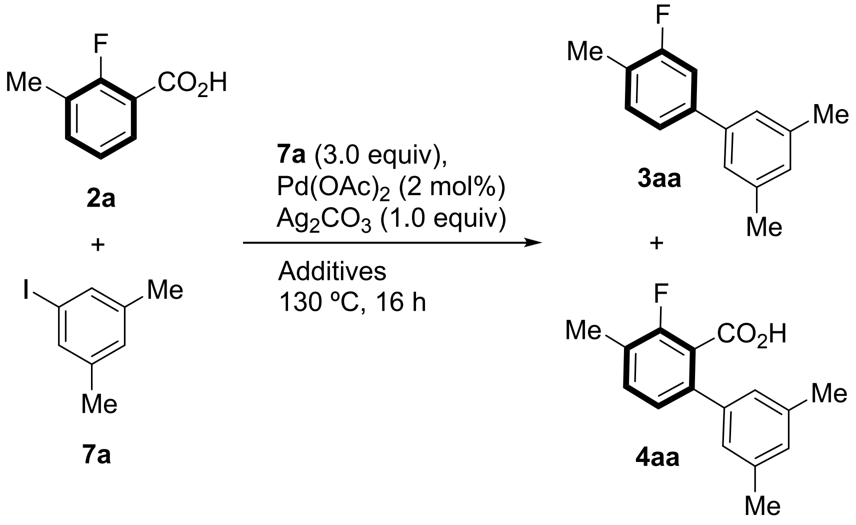

Scheme 2. Pd-catayzed arylation/decarboxylation of 2-fluoro-3-methylbenzoic acid.

Table 1. Pd-catayzed arylation/decarboxylation of 2-fluoro-3-methylbenzoic acid Yield of isolated pure product.

Entry	Additives (equiv)	**3aa** [%][a]	**4aa** [%][a]
1	AcOH (3.5)	77	0
2	TFA (1.5)<br>LiTFA (1.0)	79%	traces

A crimpable glass vial (CEM Microwave Technologies, 10 mL volume) was loaded with 2-fluoro-3-methylbenzoic acid (77.1 mg, 0.5 mmol), $Pd(OAc)_2$ (2,2 mg, 0.01 mmol), Ag_2CO_3 (137.9 mg, 0.5 mmol), 5-iodo-*m*-xylene (216 µl, 1.5 mmol), LiTFA (0-0.5 mmols) and acetic acid or trifluoroacetic acid (100 µl). The vial was capped with a Teflon lined septum and the slurry was heated at 130 °C for 16 hours. Upon completion of the reaction, 0.1 mLof a 2M solution of HCl in diethyl ether were added to the mixture and was further diluted with diethyl ether. The mixture was filtered through a plug of Celite® and concentrated *in vacuo*. The crude product was purified by column chromatography (petroleum ether) to afford 3-fluoro-3',4,5'-trimethyl-1,1'-biphenyl (**3aa**) as a colourless oil.

Experimental procedures and characterisation data for *m*-arylated aryl alkyl ethers and acetals.

General procedure D: A flame-dried crimpable glass vial (CEM Microwave Technologies, 10 mL volume) was loaded with 1.25 mL of a 0.4 M solution of aryl alkyl ether or acetal in dry THF. The solution was cooled down to the given temperature and 0.36 mL of a freshly titrated solution 1.4 M of *sec*-butyl lithium in cyclohexane were added dropwise. After reacting for the given time at the given temperature, CO_2 was bubbled into the reaction mixture. The resulting mixture was warmed up to room temperature and the solvents were removed under a stream of nitrogen. To the resulting white solid, $Pd(OAc)_2$ (2,2 mg, 0.01 mmol), Ag_2CO_3 (137.9 mg, 0.5 mmol), aryl iodide (0.25-1.5 mmol) and acetic acid (150 µl) were added, the vial was capped with a Teflon lined septum and the slurry was heated at 130 °C for 16 hours. Upon completion of the reaction, the mixture was diluted with diethyl ether and filtered through a plug of Celite® and concentrated *in vacuo*. Purification by column chromatography using the eluents specified afforded the pure *m*-arylated products.

3'-methoxy-3,5-dimethyl-1,1'-biphenyl

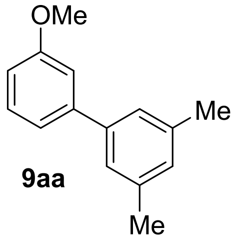

9aa

Following the general procedure D running the lithiation/carboxylation step for 1 hour at -40 °C, ansiole **8a** (0.50 mmol) was reacted with 1-iodo-3,5-dimethylbenzene **7a** (216 µl, 1.5 mmol). The crude product was purified by column chromatography (hexanes:EtOAc 98:2) using small particle size silica (0.015-0.04 mm) to afford 3'-methoxy-3,5-dimethyl-1,1'-biphenyl (**9aa**) as a colourless oil (53.2 mg, 50%). Spectroscopic data matched the previously reported.[1] **^{1}H NMR** (400 MHz, $CDCl_3$) δ, ppm: 7.35 (t, J = 7.9 Hz, 2H), 7.22 (s, 2H), 7.18 (ddd, J = 7.6 Hz, 1.6 Hz, 1.0 Hz, 1H), 7.13-7.12 (m), 7.01 (s, 1H), 6.89 (ddd, J = 8.2 Hz, 2.6 Hz, 0.9 Hz, 1H), 3.88 (s, 3H), 2.39 (s, 6H). **^{13}C NMR** (101 MHz, $CDCl_3$) δ, ppm: 160.0, 143.2, 141.3, 138.4, 129.7, 129.2, 125.3, 119.9, 113.0, 112.7, 55.4, 21.5. **HRMS** (APCI, **[M-H]$^+$**) *m/z* calculated for $C_{15}H_{17}O^+$, 213.1274, found: 213.1273.

3-methoxy-3',5,5'-trimethyl-1,1'-biphenyl

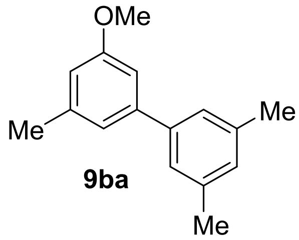

9ba

Following the general procedure D running the lithiation/carboxylation step for 1.5 hours at -40 ° C, 3-methylanisole **8b** (0.50 mmol) was reacted with 1-iodo-3,5-dimethylbenzene **7a** (37 µl, 0.25 mmol) using 1.1 mg (0.005 mmol) of $Pd(OAc)_2$, 69 mg (0.25 mmol) of Ag_2CO_3 and 100 µl of acetic acid. The crude product was purified by column chromatography on silica (hexanes:EtOAc 98:2) to afford 3-methoxy-3',5,5'-trimethyl-1,1'-biphenyl (**6ba**) as a colourless oil (24.2 mg, 43%). **IR** (ATR), ν, cm^{-1}: 1588, 906, 732. **^{1}H NMR** (400 MHz, $CDCl_3$) δ, ppm: 7.19 (s, 2H), 7.00 (s, 2H), 6.92 (s, 1H), 6.71(s, 1H), 3.85 (s, 3H), 2.39 (s, 3H), 2.38 (s, 6H). **^{13}C NMR** (101 MHz, $CDCl_3$) δ, ppm: 160.0, 143.0, 141.4, 139.7, 138.3, 129.1, 125.3, 120.8, 113.6, 110.0, 55.4, 21.8, 21.5. **HRMS** (APCI, **[M-H]**$^+$) *m/z* calculated for $C_{16}H_{19}O^+$, 227.1430, found: 227.1430.

3,5-dimethyl-3'-(trifluoromethoxy)-1,1'-biphenyl

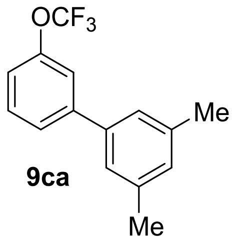

9ca

Following the general procedure D running the lithiation/carboxylation step for 1 hour at -78 °C, trifluoromethoxybenzene **8c** (0.50 mmol) was reacted with 1-iodo-3,5-dimethylbenzene **7a** (216 µl, 1.5 mmol) using 100 µl of trifluoroacetic acid. The crude product was purified by column chromatography on silica (petroleum ether) to afford 3,5-dimethyl-3'-(trifluoromethoxy)-1,1'-biphenyl (**9ca**) as a colourless oil (50.2 mg, 38%). **IR** (ATR), ν, cm^{-1}: 1595, 1253, 1219, 1256, 903, 727. **^{1}H NMR** (400 MHz, $CDCl_3$) δ, ppm: 7.52-7.50 (m, 1H), 7.46-7.42 (m, 2H), 7.19-7.17 (m, 3H), 7.04 (s, 1H), 2.40 (s, 6H). **^{13}C NMR** (101 MHz, $CDCl_3$) δ, ppm: 149.7 (q, J_{CF} = 1.8 Hz), 143.7, 139.8, 138.7, 130.0, 129.8, 125.7, 125.2, 120.7 (q, J_{CF} = 258.0 Hz), 119.9, 119.5, 21.5. **^{19}F-NMR** (376 MHz, $CDCl_3$) δ, ppm: -57.66 (s). **HRMS** (EI, **M-H]**$^+$) *m/z* calculated for $C_{13}H_{11}OF_3^+$, 266.0913, found: 266.0904.

5-(3,5-dimethylphenyl)-2,2-difluorobenzo[*d*][1,3]dioxole

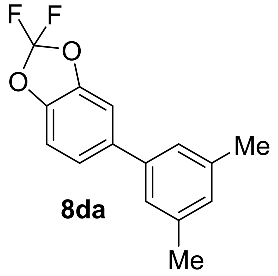

8da

Following the general procedure D running the lithiation/carboxylation step for 1 hour at -78 °C, 2,2-difluorobenzo-1,3-dioxole **8d** (0.50 mmol) was reacted with 1-iodo-3,5-dimethylbenzene **7a** (216 µl, 1.5 mmol) using 150 µl of acetic acid. The crude product was purified by column chromatography on silica (hexanes) to afford 5-(3,5-dimethylphenyl)-2,2-difluorobenzo[*d*][1,3]dioxole (**9da**) as a white solid (72.9 mg, 56%). Suitable crystals of **9da** for X-ray diffraction analysis were grown by slow evaporation from a concentrated solution of the compound in hexanes. **m.p.** = 55-56 °C. **IR** (ATR), ν, cm^{-1}: 1496, 1457, 1227, 1144, 1031, 847, 810. ^{1}**H NMR** (400 MHz, $CDCl_3$) δ, ppm: 7.26-7.24 (m, 2H), 7.11 (s, 2H), 7.08-7.06 (m, 1H), 7.00 (s, 1H), 2.37 (s, 6H). ^{13}**C NMR** (101 MHz, $CDCl_3$) δ, ppm: 144.3, 143.2, 140.1, 138.7, 138.2, 131.9 (t, J_{CF} = 256.0 Hz), 129.4, 125.2, 122.6, 109.5, 108.6, 21.5. ^{19}**F-NMR** (376 MHz, $CDCl_3$) δ, ppm: -50.00 (s). **HRMS** (EI, $[\mathbf{M}]^+$) *m/z* calculated for $C_{15}H_{12}O_2F_2^+$, 262.0786, found: 262.0788.

Scope of the one-pot *m*-arylation of arenes

Scope of aryl iodide coupling partners

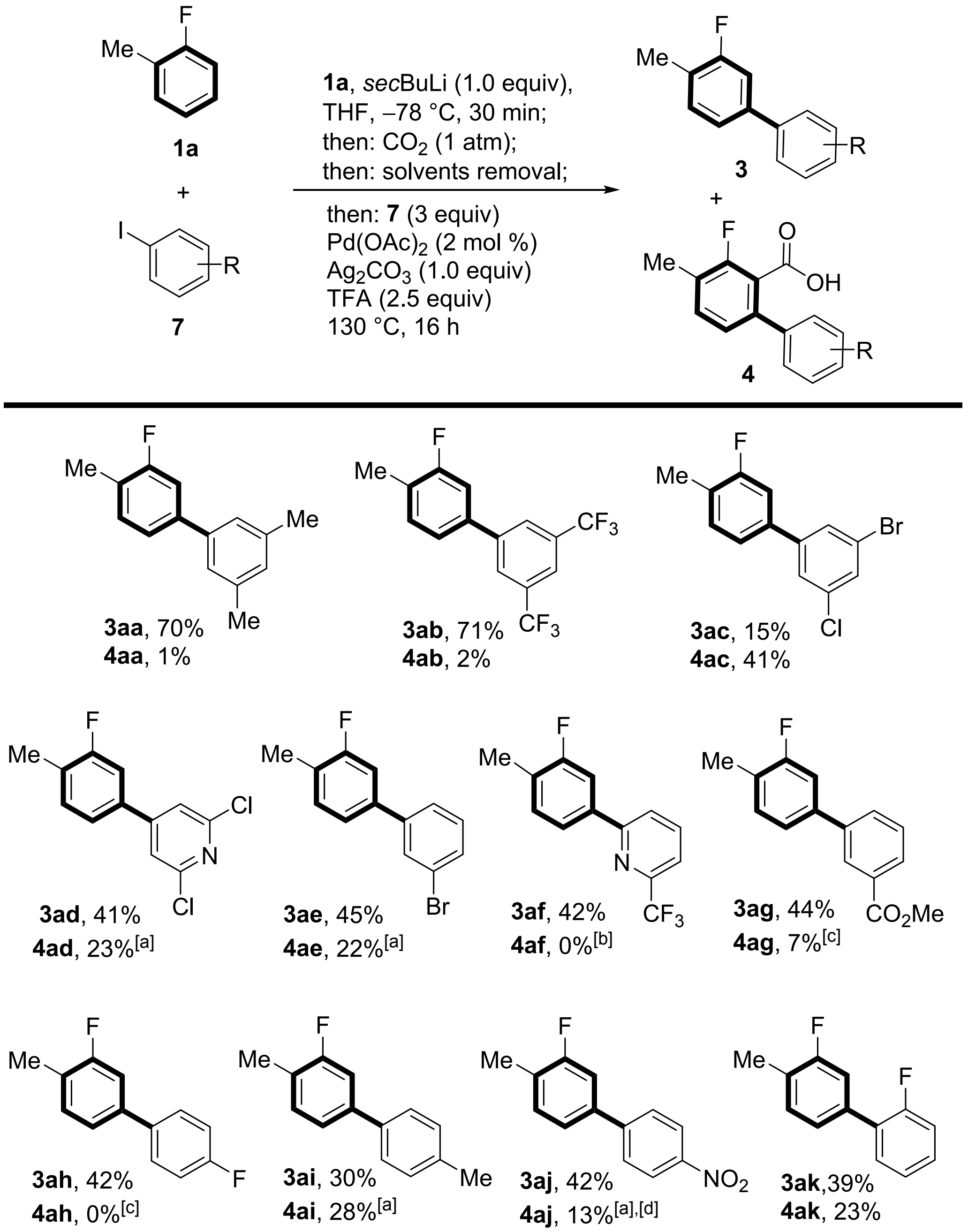

Scheme 3. Substrate scope of the *meta*-arylation of fluoroarenes with aryl iodides. Yields are of pure isolated product. [a] 1.5 equiv of **7** employed. [b] 0.5 equiv of **7**, 2 mol % of $Pd(OAc)_2$ and 0.5 equiv of Ag_2CO_3 were employed. [c] 2 equiv of **7** employed. [d] Arylation/decarboxylation step run at 150 °C.

Scope of fluoroarene coupling partners

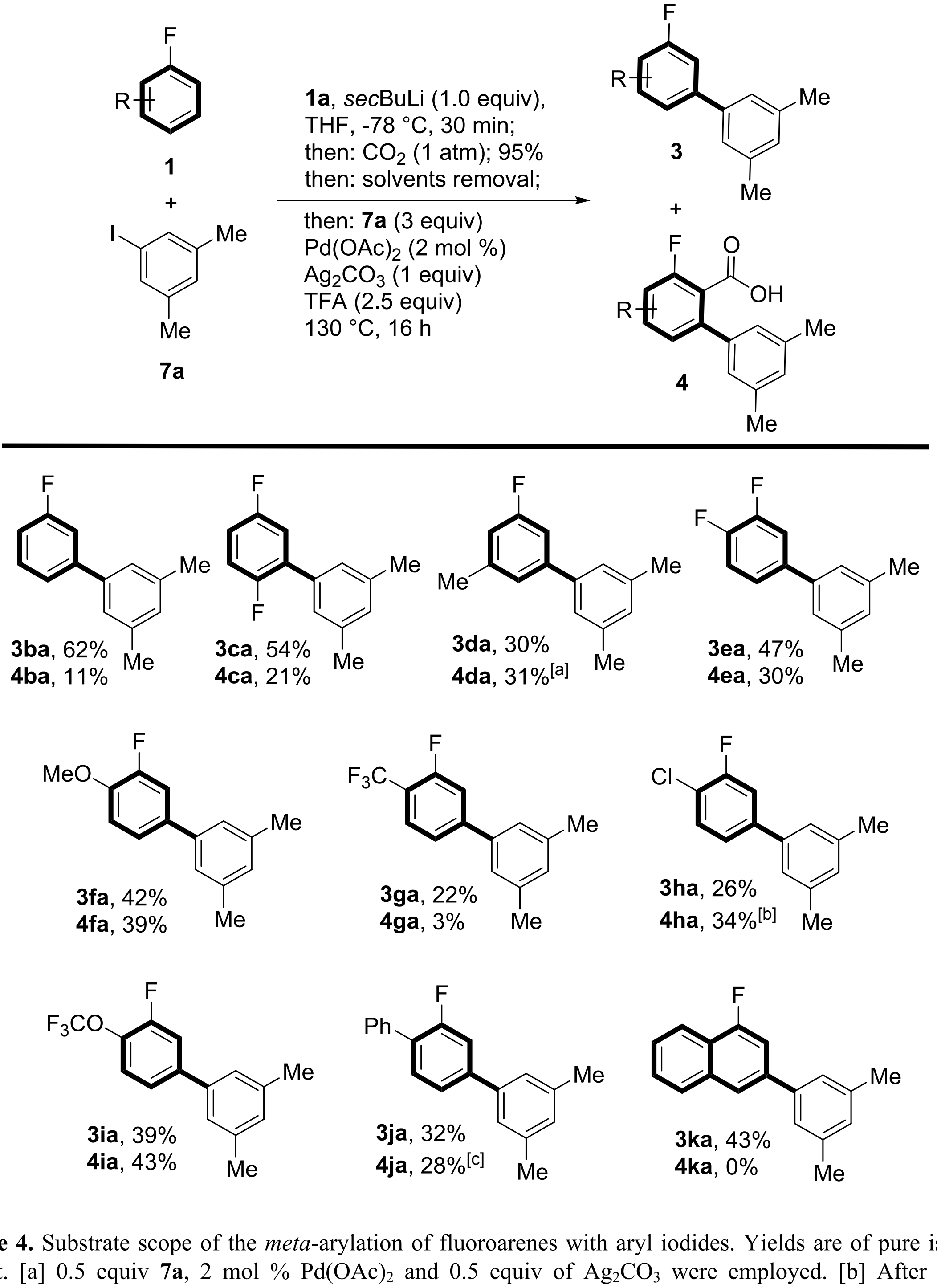

Scheme 4. Substrate scope of the *meta*-arylation of fluoroarenes with aryl iodides. Yields are of pure isolated product. [a] 0.5 equiv **7a**, 2 mol % $Pd(OAc)_2$ and 0.5 equiv of Ag_2CO_3 were employed. [b] After 6 h of arylation/decarboxylation reaction, an additional 2 mol % $Pd(OAc)_2$ was added and the reaction was stirred at 130 °C for 10 more hours. [c] 1.5 equiv **7a**.

Scope of aryl alkyl ethers and acetal coupling partners

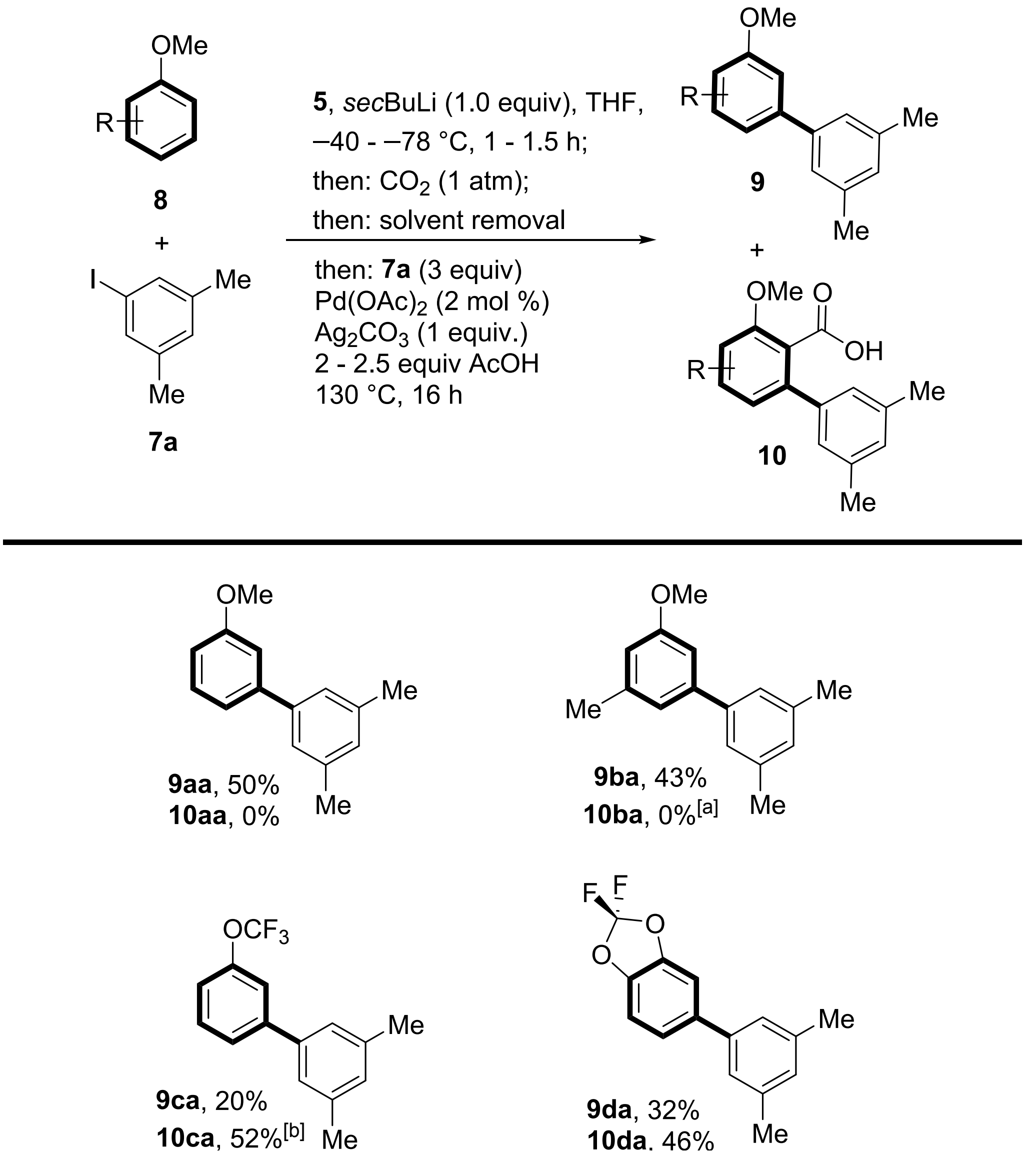

Scheme 5. Substrate scope of the *meta*-arylation of substituted arenes with aryl iodides. Yields are of pure isolated product. [a] 0.5 equiv of **7a**, 2 mol % of $Pd(OAc)_2$ and 0.5 equiv of Ag_2CO_3 were employed. [b] After 16 h, of arylation/decarboxylation reaction 2 mL of DMSO were added and the reaction was stirred at 170 °C for 3 h. [c] TFA used instead of AcOH.

Preparation of the γ-secretase inhibitor 7

3'-bromo-5'-chloro-3-fluoro-4-(trifluoromethoxy)-1,1'-biphenyl

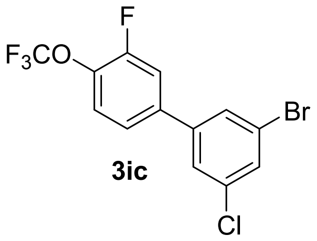

3ic

Following the general procedure B running the arylation/decarboxylation step at 130 °C, in a flame-dried crimpable glass vial (CEM Microwave Technologies, 25 mL volume), 2-(trifluoromethoxy)fluorobenzene **1i** (1.50 mmol) was reacted with 1-bromo-3-chloro-5-iodobenzene **7c** (833 mg, 2.62 mmol). The crude product was purified by column chromatography (hexanes) using small particle size silica (0.015-0.04 mm) to afford 3'-bromo-5'-chloro-3-fluoro-4-(trifluoromethoxy)-1,1'-biphenyl (**3ic**) as a colourless oil (327.0 mg, 59%). **IR**: 1324, 1254, 1216, 1164, 1146, 1124, 1069, 839 cm^{-1}. **^{1}H NMR** (400 MHz, $CDCl_3$) δ, ppm: 7.56 (t, J = 1.4 Hz, 1H), 7.54 (t, J = 1.7 Hz, 1H), 7.45 (t, J = 1.6 Hz, 1H), 7.41-7.35 (m, 2H), 7.33-7.30 (m, 1H). **^{13}C NMR** (101 MHz, $CDCl_3$) δ, ppm: 154.8 (d, J_{CF} = 254.9 Hz), 142.0 (d, J_{CF} = 1.6 Hz), 139.2 (d, J_{CF} = 6.8 Hz), 136.7 (dq, J_{CF} = 12.5 Hz, J_{CF} = 1.9 Hz), 131.1, 128.6, 126.2, 124.3, 123.5, 123.3 (J_{CF} = 3.7 Hz), 120.6 (q, J_{CF} = 260.5 Hz), 116.2 (d, J_{CF} = 19.8 Hz). **^{19}F-NMR** (376 MHz, $CDCl_3$) δ, ppm: -58.7 (d, J_{CF} = 4.5 Hz), -127.4 (m). **HRMS** (APCI, **$[M]^+$**) *m/z* calculated for $C_{13}H_6OBrCrF_4^+$, 376.9227, found:376.9233.

tert-butyl 2-(5-chloro-3'-fluoro-4'-(trifluoromethoxy)-[1,1'-biphenyl]-3-yl)-4-methylpentanoate

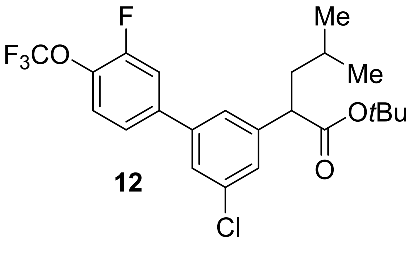

12

This reaction was conducted under an argon atmosphere. A flame-dried Schlenk tube was charged with LiHMDS (42 mg, 0.425 mmol), $Pd(OAc)_2$ (0.7 mg, 0.003 mmol), DavePhos (2.25 mg, 0.0063 mmmol) and dissolved in dry toluene (0.4 mL) in an Ar filled dry box. The Schlenk tube was sealed with a rubber septum, removed from the dry box and allowed to stir for 10 minutes at room temperature under a positive pressure of N_2. The Schlenk tube was cooled to -10°C and *tert*-butyl 4-methylpentanoate (47 µL, 0.23 mmol) was added dropwise and the resulting mixture was stirred for 10 minutes at -10 °C. To the solution of lithium enolate was added aryl bromide **3ic** (37 mg, 0.1 mmol) dropwise as a solution in dry toluene (0.25mL). The reaction mixture was allowed to warm at

room temperature and stirred at 60°C for 5 hours. Purification by column chromatography loading the crude product absorbed on silica and eluted using a gradient of hexanes:Et_2O (100:0 to 96:4) afforded *tert*-butyl 2-(5-chloro-3'-fluoro-4'-(trifluoromethoxy)-[1,1'-biphenyl]-3-yl)-4-methylpentanoate (**12**) as a colourless oil (39.1mg, 85%). **IR**: 1727, 1255, 1218, 1199, 1174, 1145, 865, 767 cm^{-1}. **^{1}H NMR** (400 MHz, $CDCl_3$) δ, ppm: 7.41-7.32 (m, 6H), 3.58 (t, J = 7.7 Hz, 1H), 2.00-1.93 (m, 1H), 1.64-1.57 (m, 1H), 1.56-1.49 (m, 1H), 1.43 (s, 9H), 0.94 (d, J = 6.5 Hz, 3H), 0.93 (d, J = 6.5 Hz, 3H). **^{13}C NMR** (101 MHz, $CDCl_3$) δ, ppm: 172.8, 154.8 (d, J_{CF} = 254.1 Hz), 142.9, 140.6, 140.6 (d, J_{CF} = 8.0 Hz), 136.3 (dq, J_{CF} = 12.6 Hz, J_{CF} = 1.9 Hz), 135.1, 128.0, 125.9, 125.1, 124.2, 123.3 (d, J_{CF} = 3.6 Hz), 120.6 (q, J_{CF} = 260.0 Hz), 116.1 (q, J_{CF} = 19.6 Hz), 81.3, 50.6, 42.9, 28.1, 26.2, 22.6, 22.6. **^{19}F-NMR** (376 MHz, $CDCl_3$) δ, ppm: -58.8 (d, J_{CF} = 4.6 Hz), -128.0 (m). **HRMS** (APCI, **[M]$^+$**) *m/z* calculated for $C_{23}H_{26}O_3ClF_4^+$, 461.1501, found: 461.1504.

2-(3-fluoro-4-(trifluoromethoxy)-4''-(trifluoromethyl)-[1,1':3',1''-terphenyl]-5'-yl)-4-methylpentanoic acid

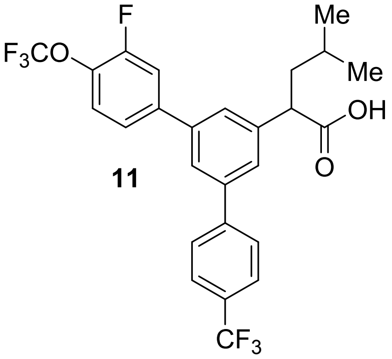

11

This reaction was conducted under an argon atmosphere. A flame-dried microwave vial was charged with aryl chloride **12** (44 mg, 0.095 mmol), 4-trifluoromethylboronic acid (27 mg, 0.14 mmol), $Pd(OAc)_2$ (1.07 mg, 0.005 mmol), SPhos (3.9 mg, 0.0095 mmols), K_3PO_4 (40 mg, 0.19 mg) and dissolved in dry THF (0.25 mL) in an Ar filled dry box. The Schlenk tube was sealed with a rubber septum and allowed to stir for 18 h at 80 ºC. The microwave vial was allowed to cool down to room temperature and 0.32 ml of a mixture 3:10 TFA:MeCN was added to the reaction mixture and stirred for 18 hours at 80 ºC. Purification by column chromatography loading the crude product absorbed on silica and eluted using a gradient of hexanes:EtOAc:AcOH (100:0 to 86:12:2) afforded 2-(3-Fluoro-4-trifluoromethoxy-4''-trifluoromethyl-[1,1';3',1'']terphenyl-5'-yl)-4-methyl-pentanoic acid (**7**) as an off white solid (40.9 mg, 83%). Spectroscopic data matched that that was previously reported.[3] **^{1}H NMR** (400 MHz,C_6D_6) δ 7.53 (s, 1H), 7.45 – 7.40 (m, 3H), 7.25 – 7.18 (m, 3H), 7.01 (dd, J = 10.9 Hz, 2.1 Hz, 1H), 6.93 (m, 1H), 6.82 (m, 1H), 3.83 (t, J = 7.8 Hz, 1H), 2.11 (dt, J = 13.6 Hz, 7.7 Hz, 1H), 1.70 (dt, J = 13.9 Hz, 7.1 Hz, 1H), 1.54 (hept, J = 6.7 Hz, 1H), 0.83 (dd, J = 6.5 Hz, 5.1 Hz, 6H). **^{13}C NMR** (126 MHz, $CDCl_3$) δ 180.0, 154.8 (d, J_{CF} = 253.1 Hz), 144.0, 141.4 (d, J_{CF} =6.7 Hz) 141.3,

140.5, 140.3, 136.2 (dd, J_{CF} = 12.5 Hz, 1.9 Hz), 130.1 (q, J_{CF} = 32.5 Hz), 127.8, 127.1, 126.8, 126.0 (d, J_{CF} = 3.8 Hz), 125.5, 124.3 (q, J_{CF} = 272.0 Hz) 124.2, 123.4 (d, J_{CF} = 3.5 Hz), 120.7 (q, J_{CF} = 259.0 Hz) 116.3 (d, J_{CF} = 19.4 Hz), 49.7, 42.3, 26.0, 22.7, 22.4. **^{19}F-NMR** (376 MHz, C_6D_6) δ -58.72 (d, J = 4.8 Hz), -62.13, -127.84 (ddd, J = 11.4 Hz, 8.9 Hz, 5.1 Hz).

Crystallographic data

Single crystal X-ray diffraction data were collected for crystal structures **3ab** and **6da** at a temperature of 150 K using Mo-Kα radiation on an Agilent Supernova, equipped with an Oxford Cryosystems Cobra nitrogen flow gas system. Data were measured using CrysAlisPro suite of programs.

X-ray data was processed and reduced using CrysAlisPro suite of programs for crystals **3ab** and **6da**. Absorption correction was performed using empirical methods based upon symmetry-equivalent reflections combined with measurements at different azimuthal angles using SCALE3 ABSPACK. The crystal structure was solved and refined against all F2 values using the SHELX and Olex 2 suite of programs.[4,5] All the non-hydrogen atoms were refined anisotropically. Hydrogen atoms were placed in calculated positions and refined using idealized geometries (riding model) and assigned fixed isotropic displacement parameters. The CF_3 moieties were disordered and modelled over two different positions. The C–F distances were restrained to be equal using SHELX command SADI. The atomic displacement parameters were also restrained using SHELX commands SIMU and RIGU.

Crystallographic data of compound 3ab

CCDC 1533208 and CCDC 1549121 contain the supplementary crystallographic data for **3ab** and **6da** respectively. These data can be obtained free of charge via www.ccdc.cam.ac.uk/conts/retrieving.html (or from the Cambridge Crystallographic Data Centre, 12 Union Road, Cambridge CB21EZ, UK; fax: (+44)1223-336-033; or deposit@ccdc.cam.ac.uk).

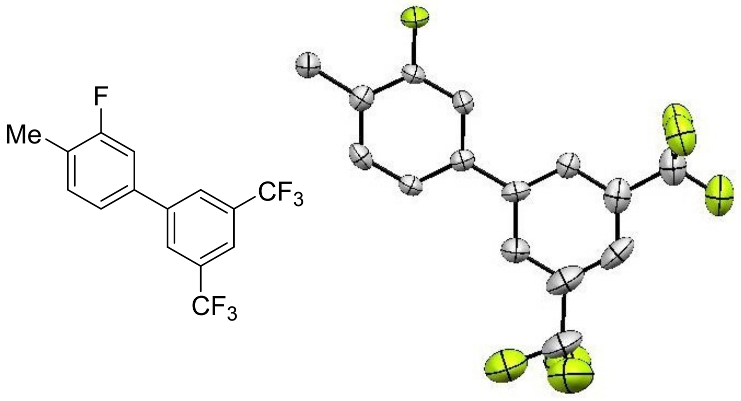

Table 2. Crystallographic information for **3ab**

Compound	**3ab**
Empirical formula	$C_{15}H_9F_7$
Formula weight	323.08
Wavelength, Å	0.71073
Temperature, K	150
Crystal colour	colourless
Crystal size, mm	0.25 × 0.25 × 0.02
Crystal system	orthorombic
Space group, *Z*	Pbcm, *40*
a, Å	12.0690(5)
α, deg	90.00
b, Å	30.5791(11)
β, deg	90.00
c, Å	36.076(2)
γ, deg	90.00
V, $Å^3$	13314.1(10)
Density, $mg.m^{-3}$	1.612
μ(Mo-Kα), mm^{-1}	0.163
2Θ range, °	6.41 to 49.424
Reflns collected	43850
Independent reflns (R_{int})	11520 (0.0882)
L.S. parameters (*p*)	1151
No. of restraints (*r*)	285
$R1$ $(F)^a$ $I > 2.0\sigma(I)$	0.1041
$wR2(F^2)^b$, all data	0.3542
$S(F^2)^c$, all data	1.035

^{a} $R1(F) = \Sigma(|F_o| - |F_c|)/\Sigma|F_o|$; ^{b} $wR2(F^2) = [\Sigma w(F_o^2 - F_c^2)^2/\Sigma wF_o^4]^{½}$; ^{c} $S(F^2) = [\Sigma w(F_o^2 - F_c^2)^2/(n + r - p)]^{½}$

Crystallographic data of compound 9da

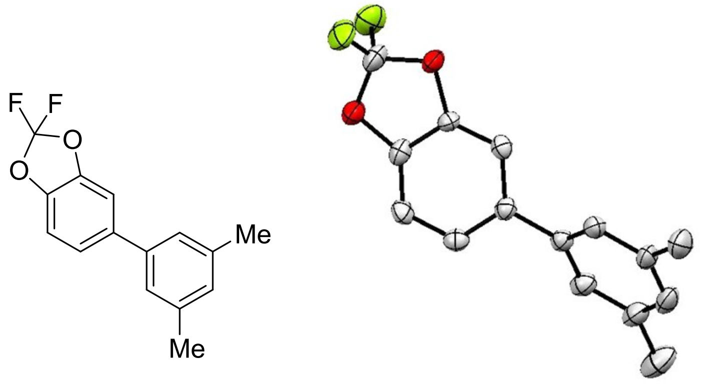

Table 3. Crystallographic information for **9da**

Compound	**9da**
Empirical formula	$C_{15}H_{12}F_2O_2$
Formula weight	262.25
Wavelength, Å	0.71073
Temperature, K	150
Crystal colour	colourless
Crystal size, mm	0.35 × 0.1 × 0.1
Crystal system	monoclinic
Space group, *Z*	P21/c, *4*
a, Å	13.442(2)
α, deg	90.00
b, Å	13.4424(16)
β, deg	93.892(14)
c, Å	7.0546(2)
γ, deg	90.00
V, Å^3	1271.8(3)
Density, mg.m^{-3}	1.370
μ(Mo-Kα), mm^{-1}	0.109
2Θ range, °	7.362 to 45.55
Reflns collected	4604
Independent reflns (R_{int})	2332 (0.0566)
L.S. parameters (*p*)	174
No. of restraints (*r*)	0

$R1\ (F)^a\ I > 2.0\sigma(I)$	0.0661
$wR2(F^2)^b$, all data	0.1633
$S(F^2)^c$, all data	1.016

[a] $R1(F) = \Sigma(|F_o| - |F_c|)/\Sigma|F_o|$; [b] $wR2(F^2) = [\Sigma w(F_o^2 - F_c^2)^2/\Sigma wF_o^4]^{½}$; [c] $S(F^2) = [\Sigma w(F_o^2 - F_c^2)^2/(n + r - p)]^{½}$

References

[1] J. Cornella, M. Righi and I. Larrosa, *Angew. Chem. Int. Ed.* 2011, **50**, 9429-9432.

[2] M. Simonetti, G. J. P. Perry, X. C. Cambeiro, F. Juliá-Hernández, J. N. Arokianathar and I. Larrosa, *J. Am. Chem. Soc.* 2016, **138**, 3596-3606.

[3] C. Y. Ho (Janssen Pharmaceutica N. V.), *US Pat*., 20070981257P, 2007

[4] G. M. Sheldrick, *Acta Crystallogr.* 2015, **C71**, 3-8.

[5] O. V. Dolomanov, L. J. Bourhis, R. J. Gildea, J. A. K. Howard and H. Puschmann, *J. Appl. Cryst.* 2009, **42**, 339-341.

NMR spectral data

^{1}H NMR

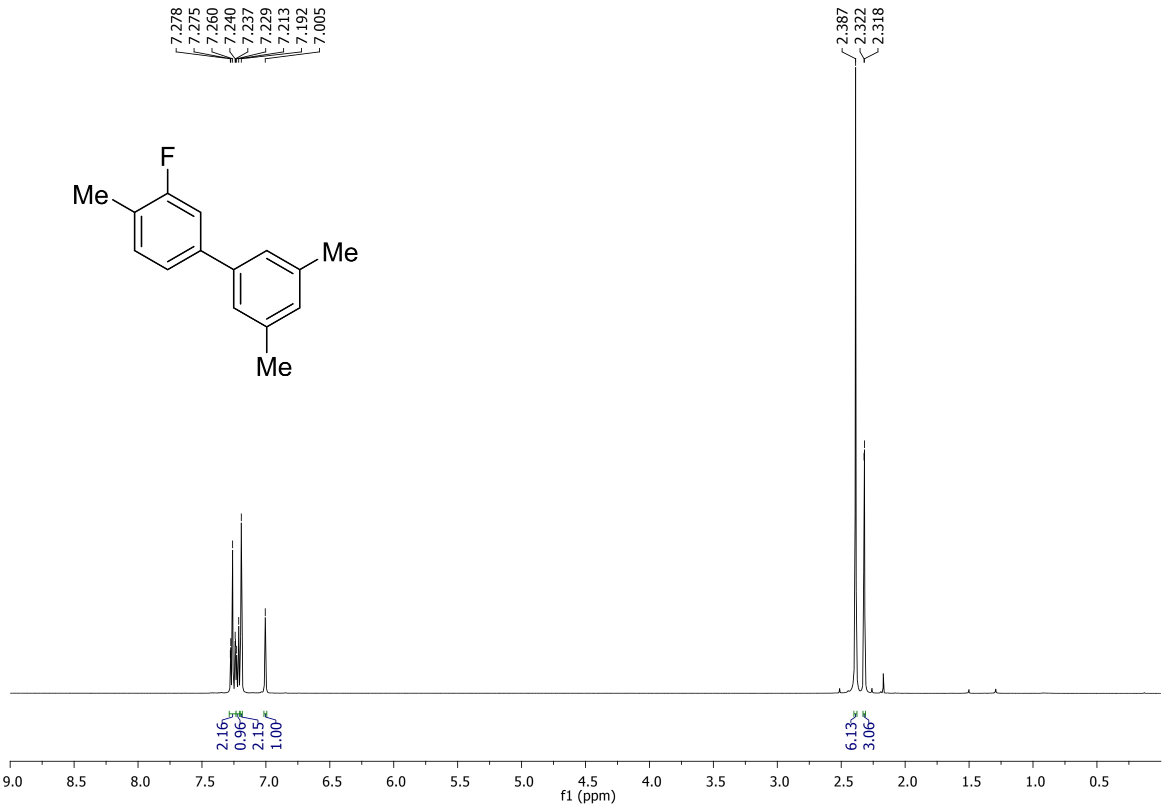

^{13}C NMR

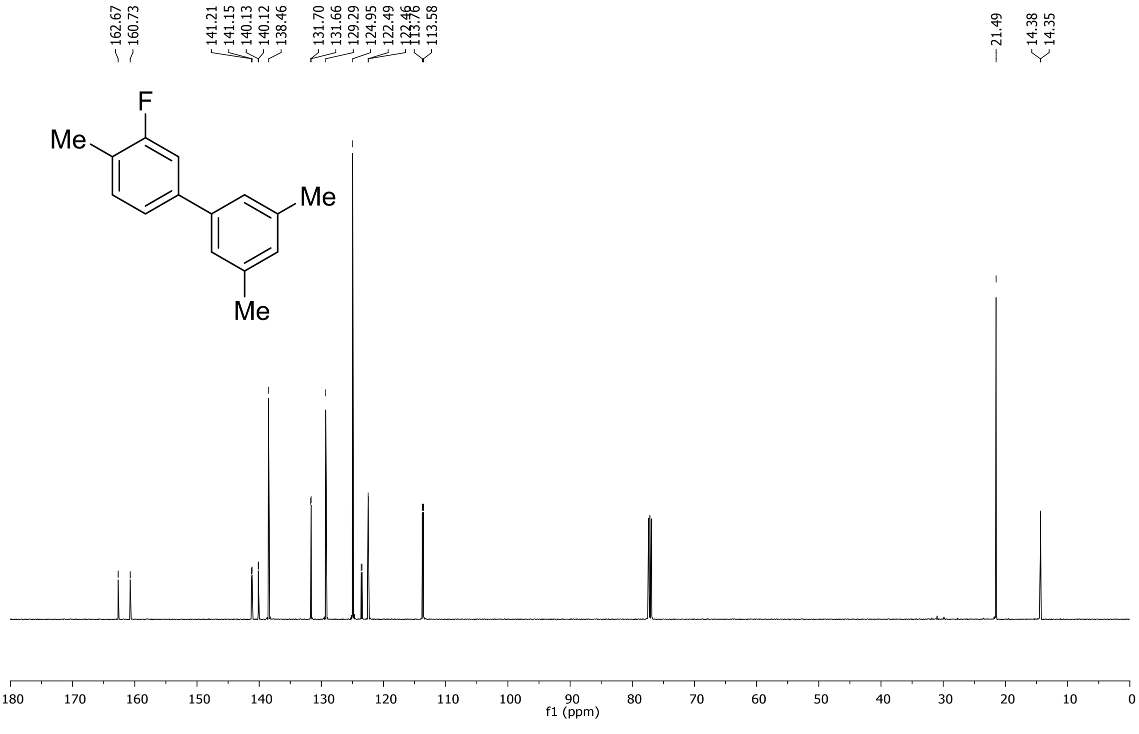

^{19}F NMR

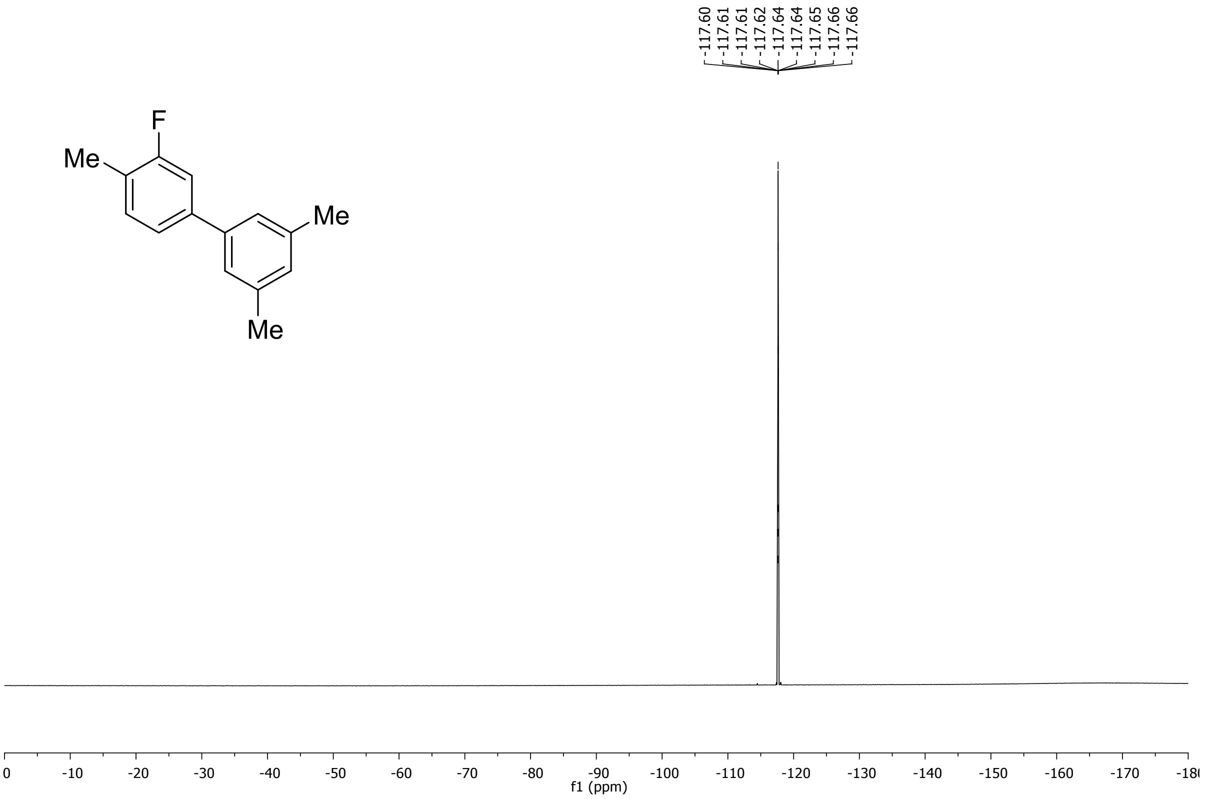

^{1}H NMR

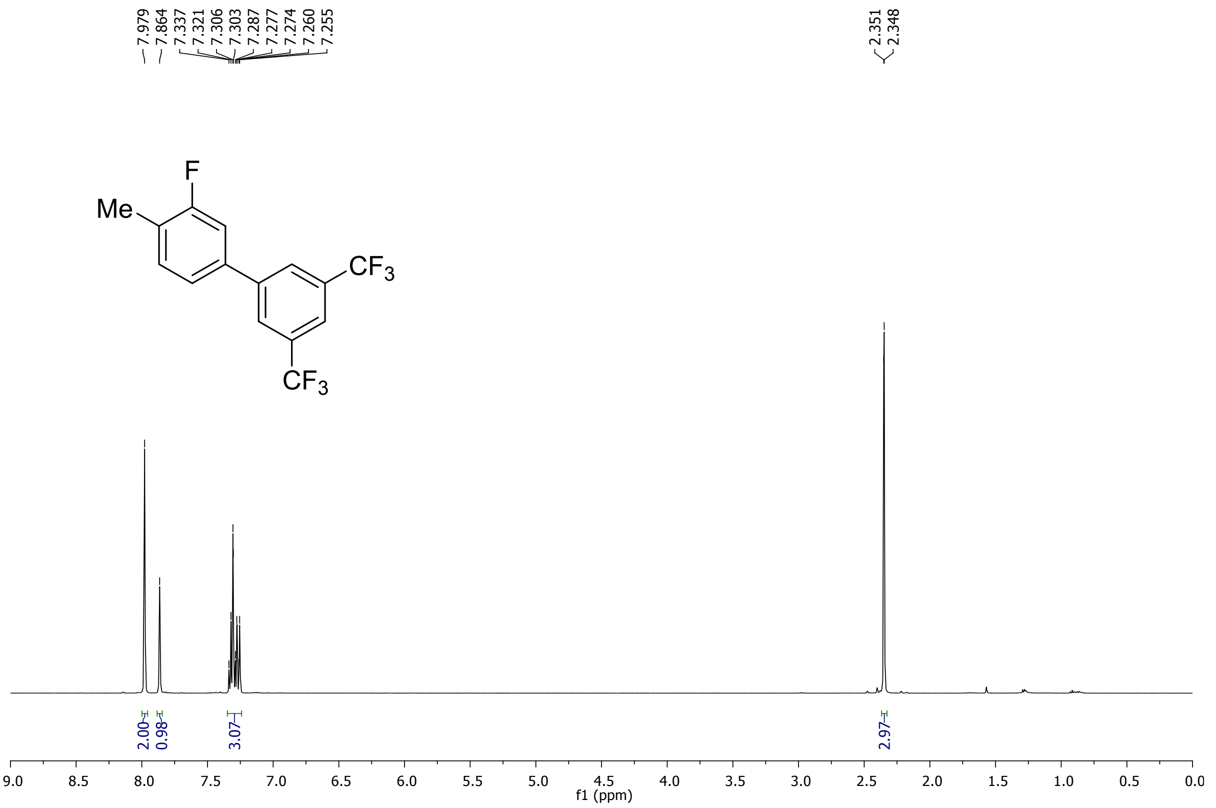

^{13}C NMR

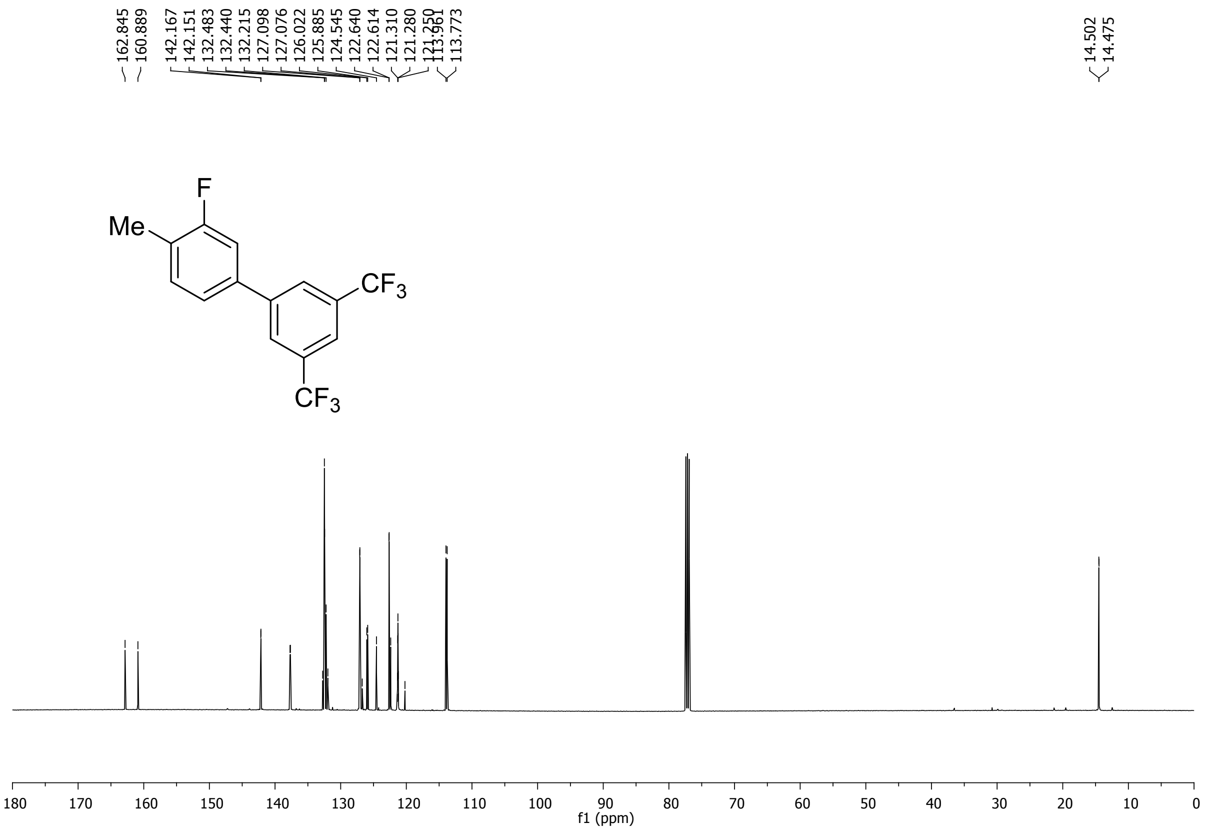

^{19}F NMR

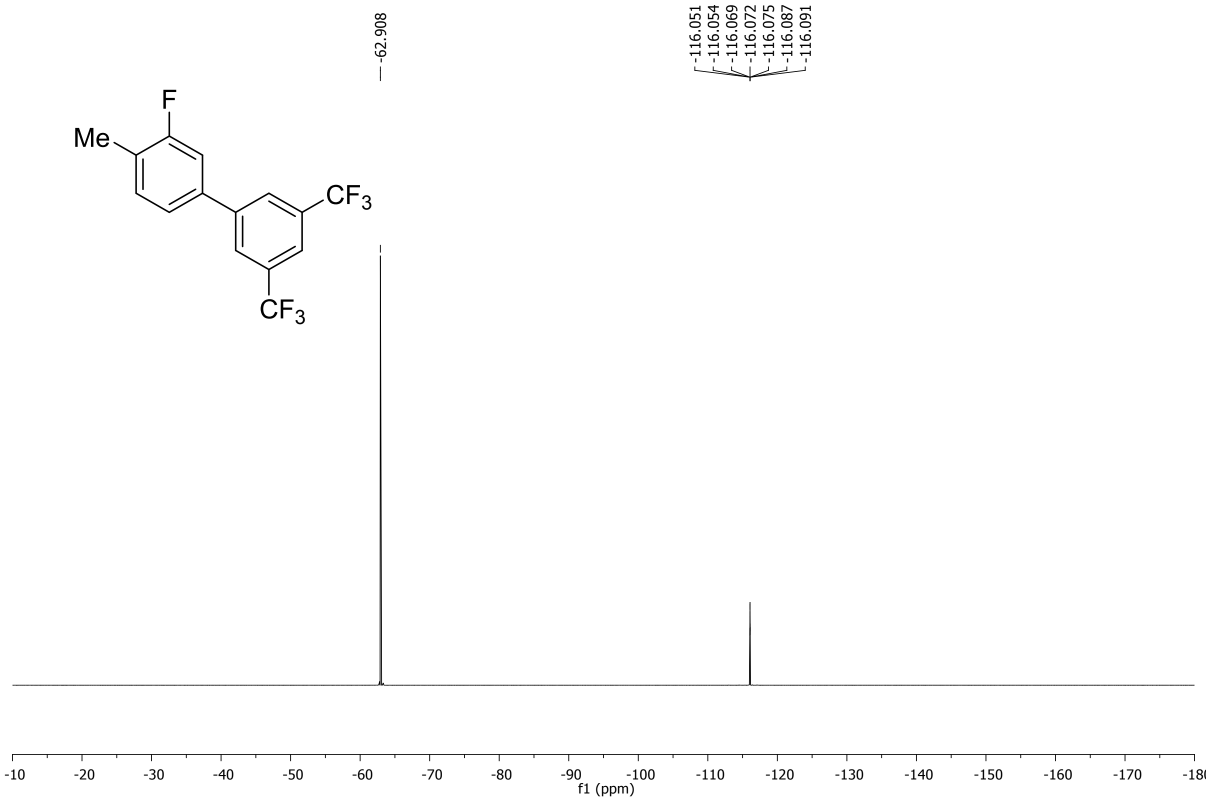

^{1}H NMR

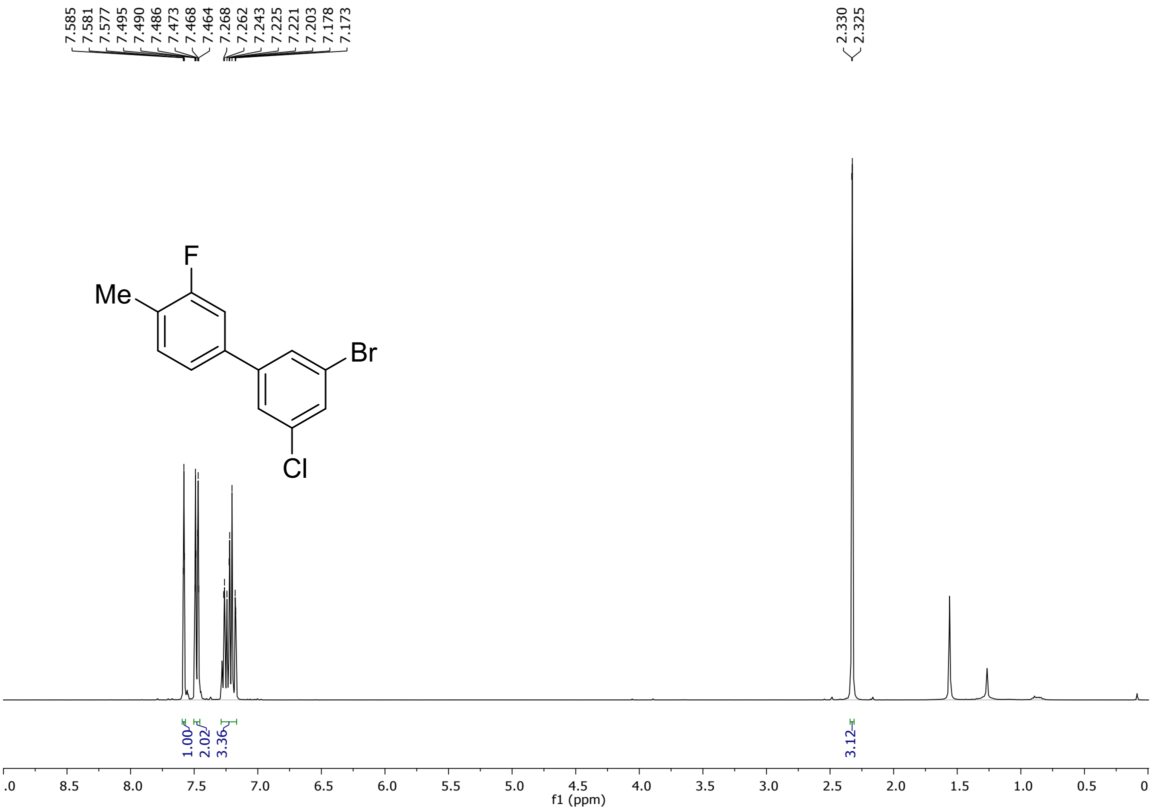

^{13}C NMR

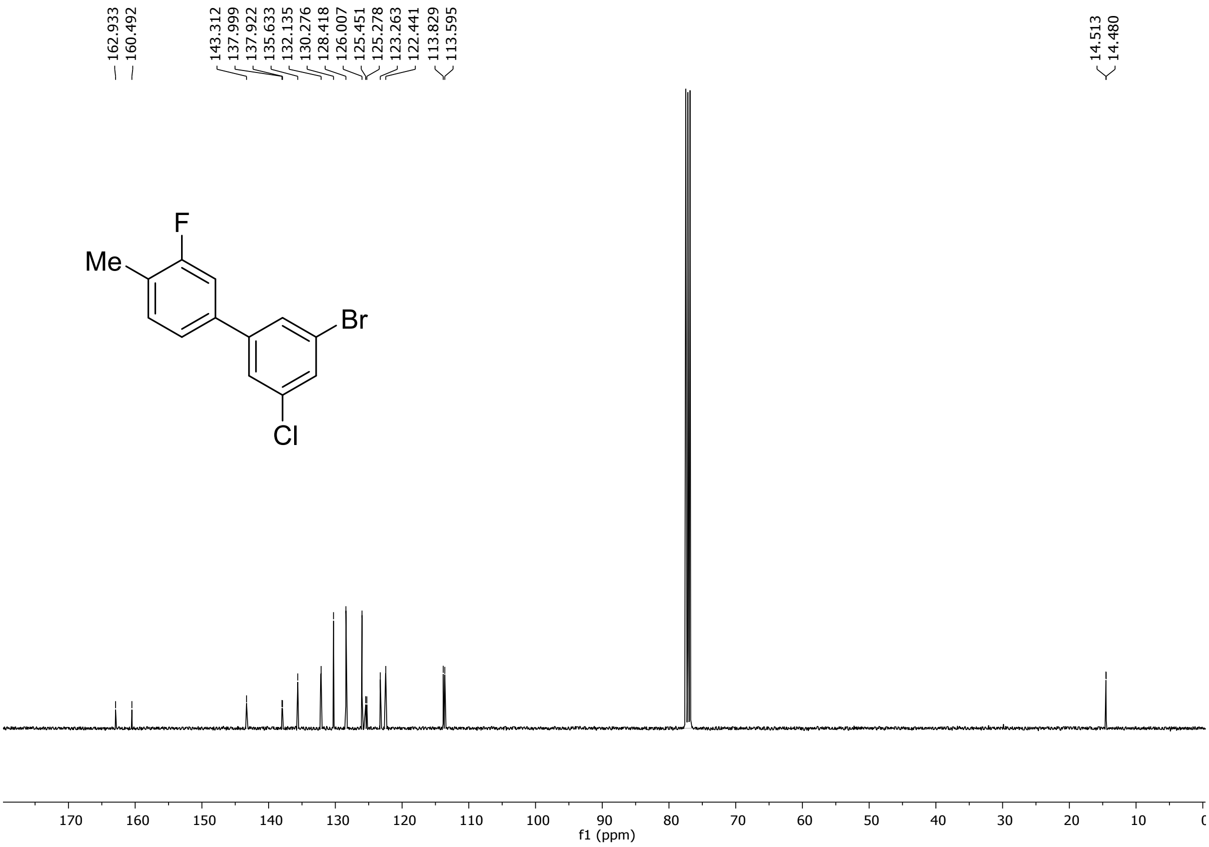

^{19}F NMR

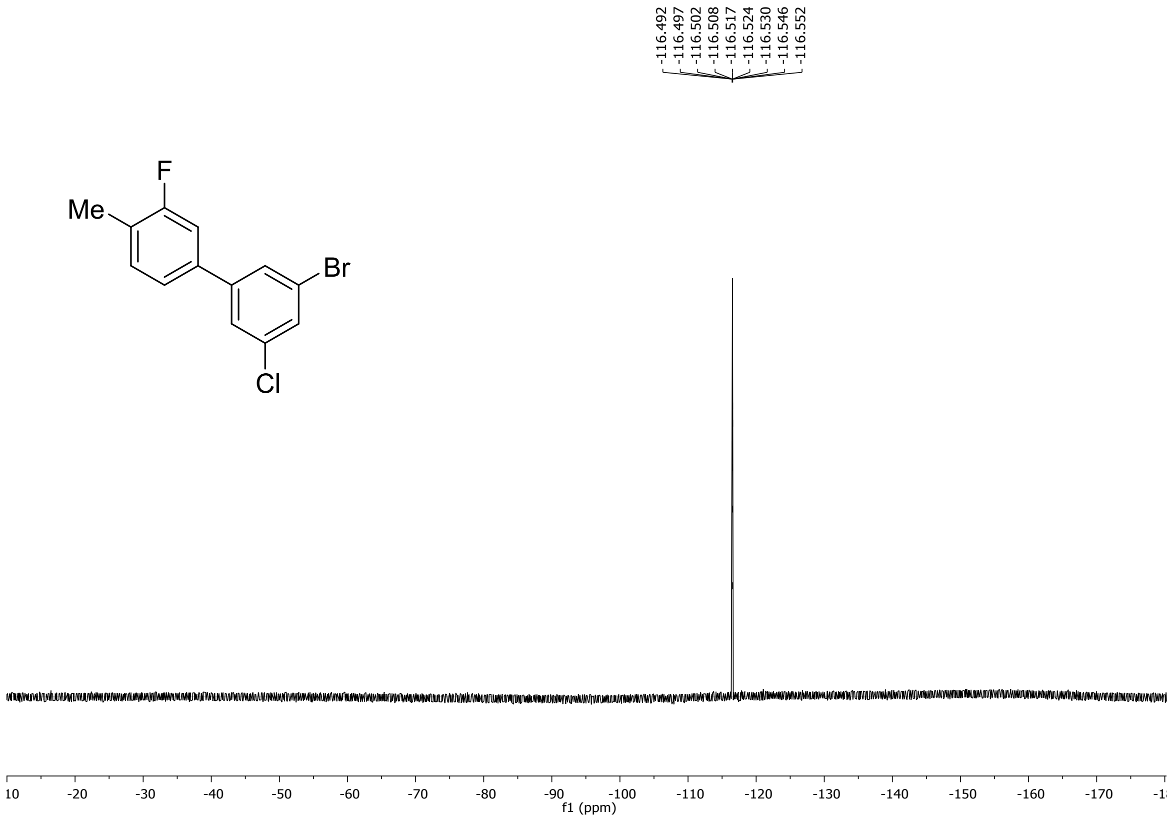

^{1}H NMR

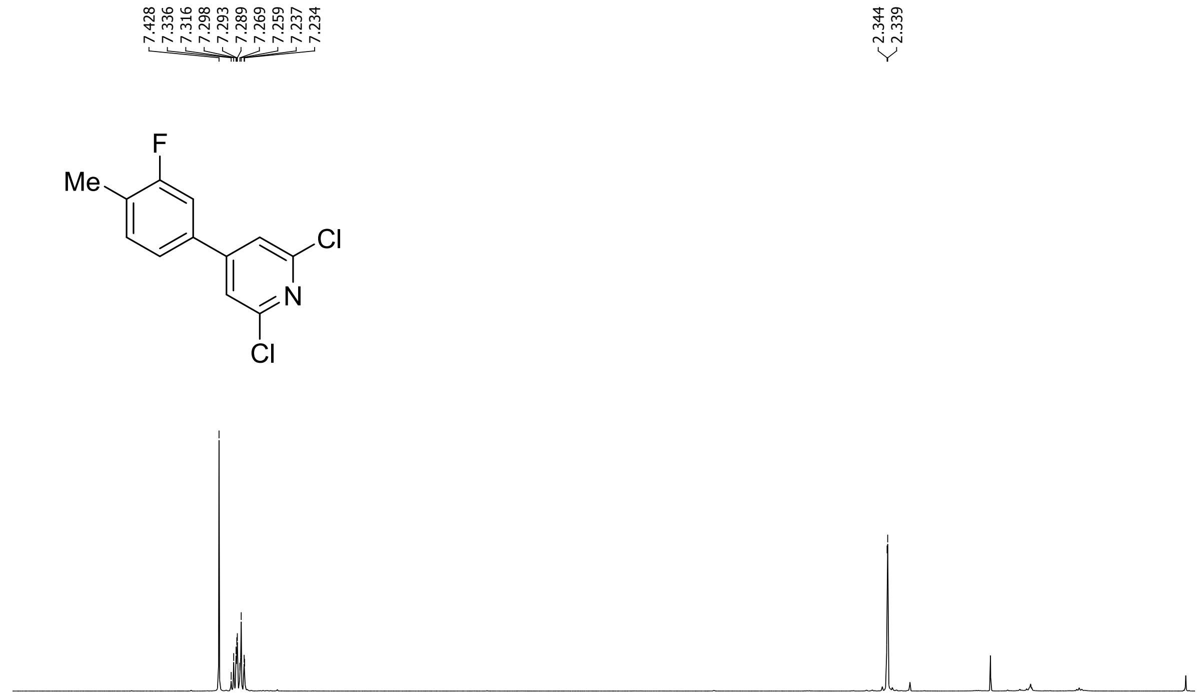

^{13}C NMR

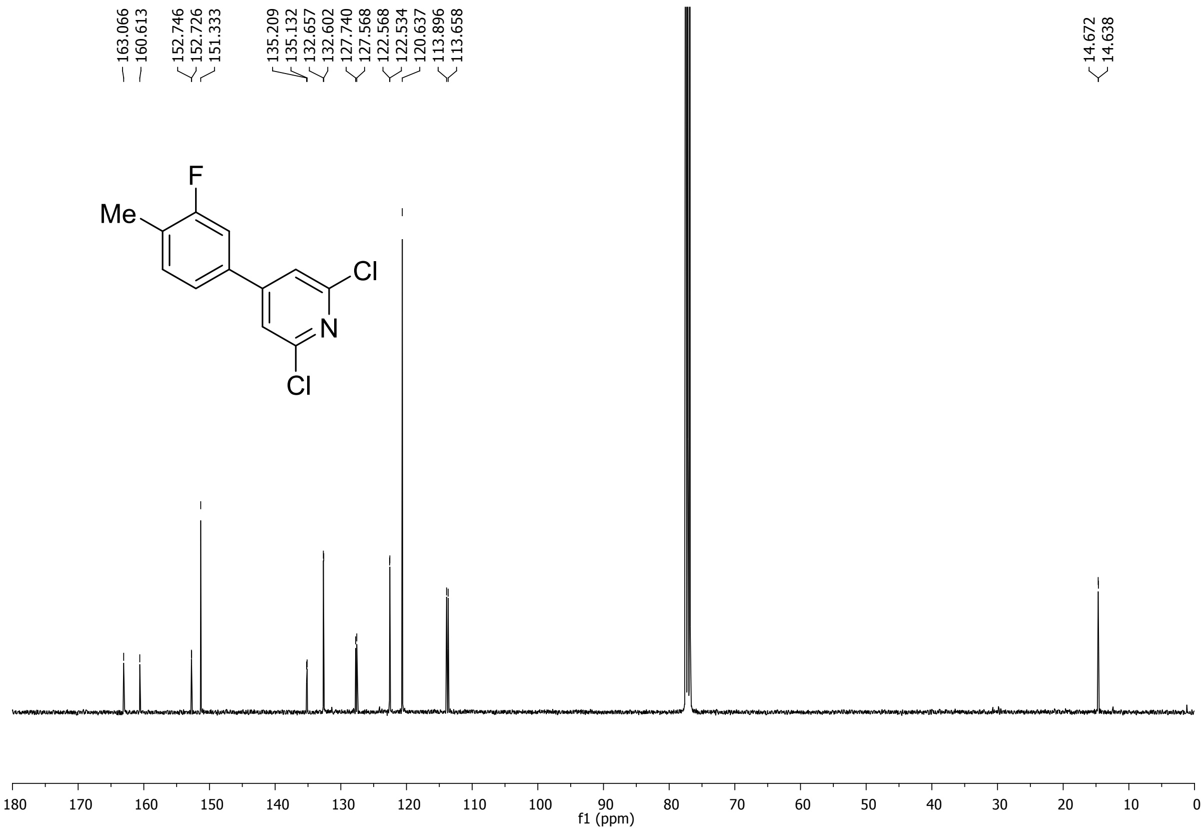

^{19}F NMR

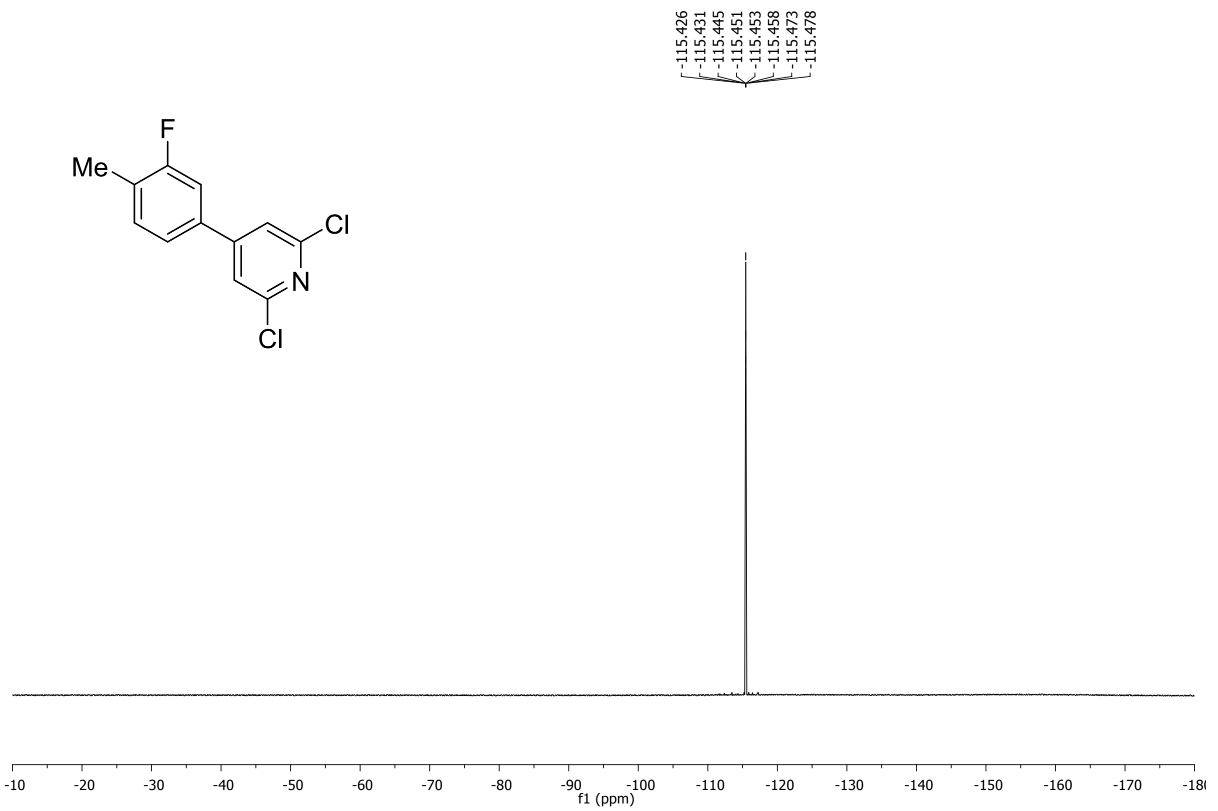

^{1}H NMR

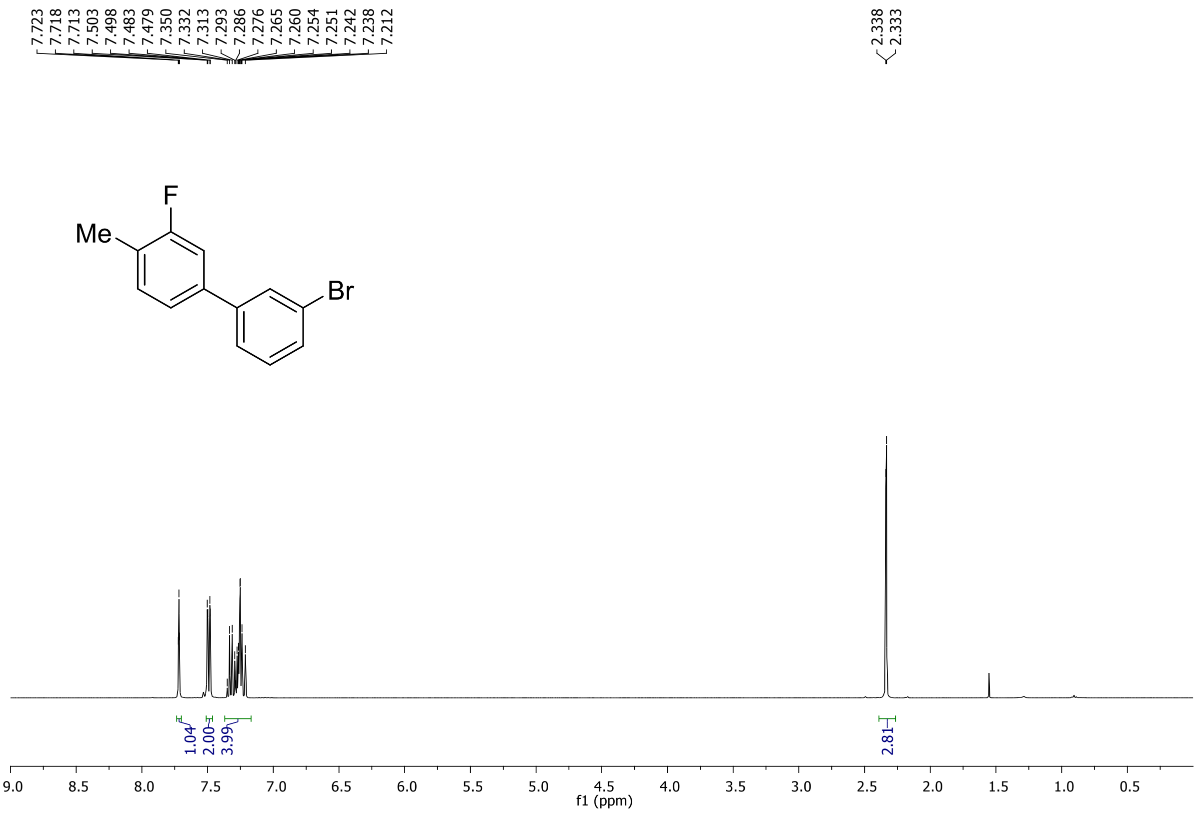

^{13}C NMR

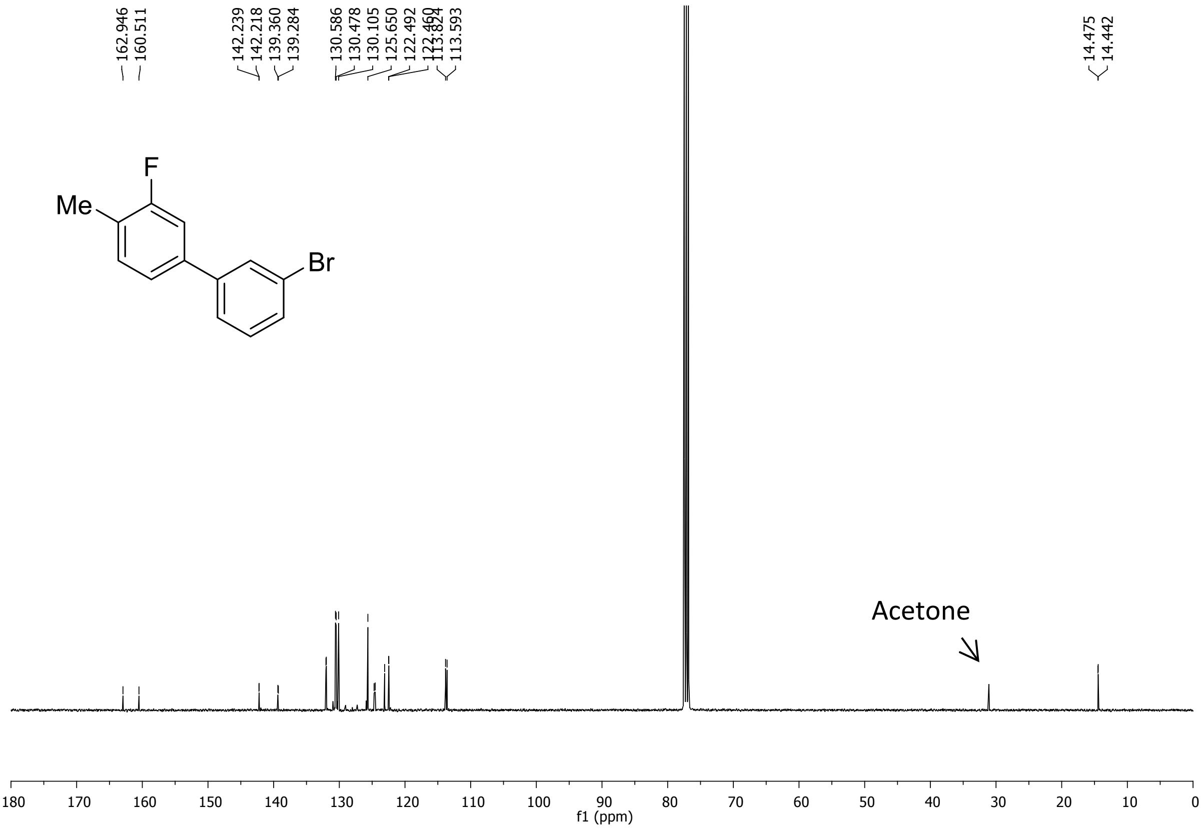

^{19}F NMR

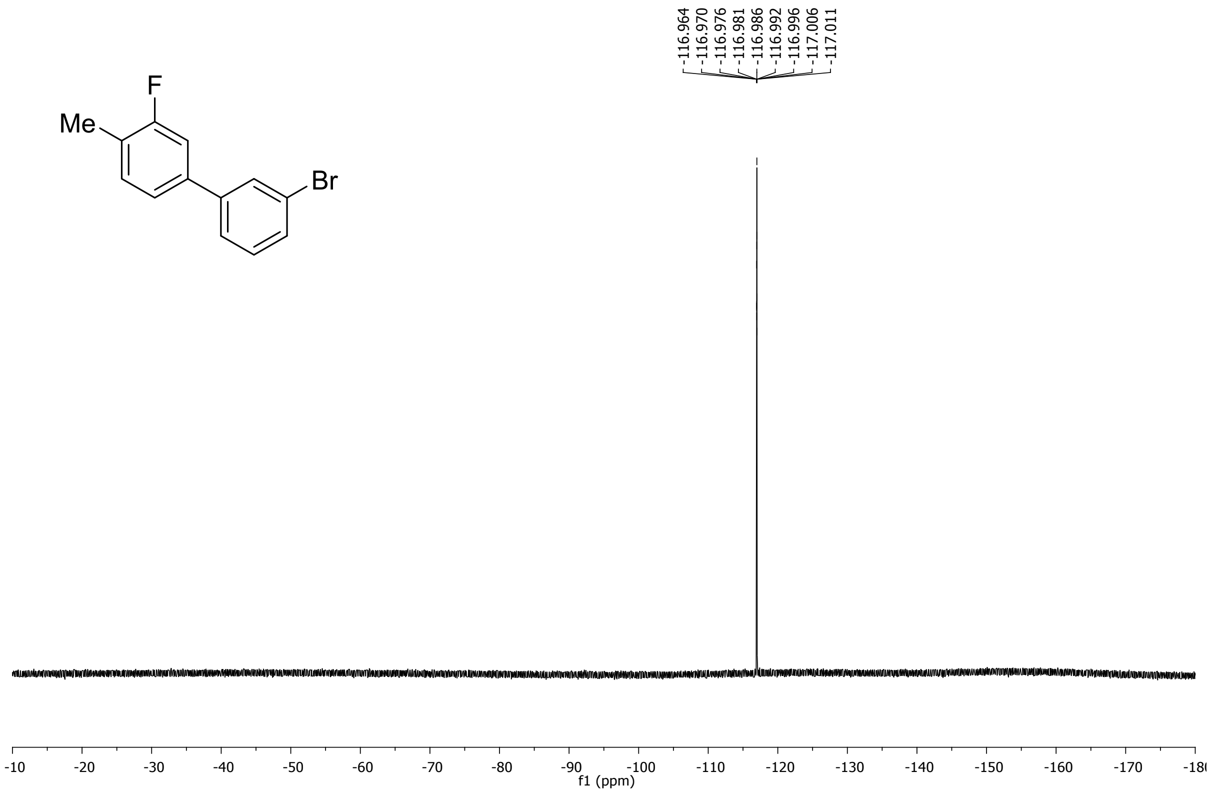

^{1}H NMR

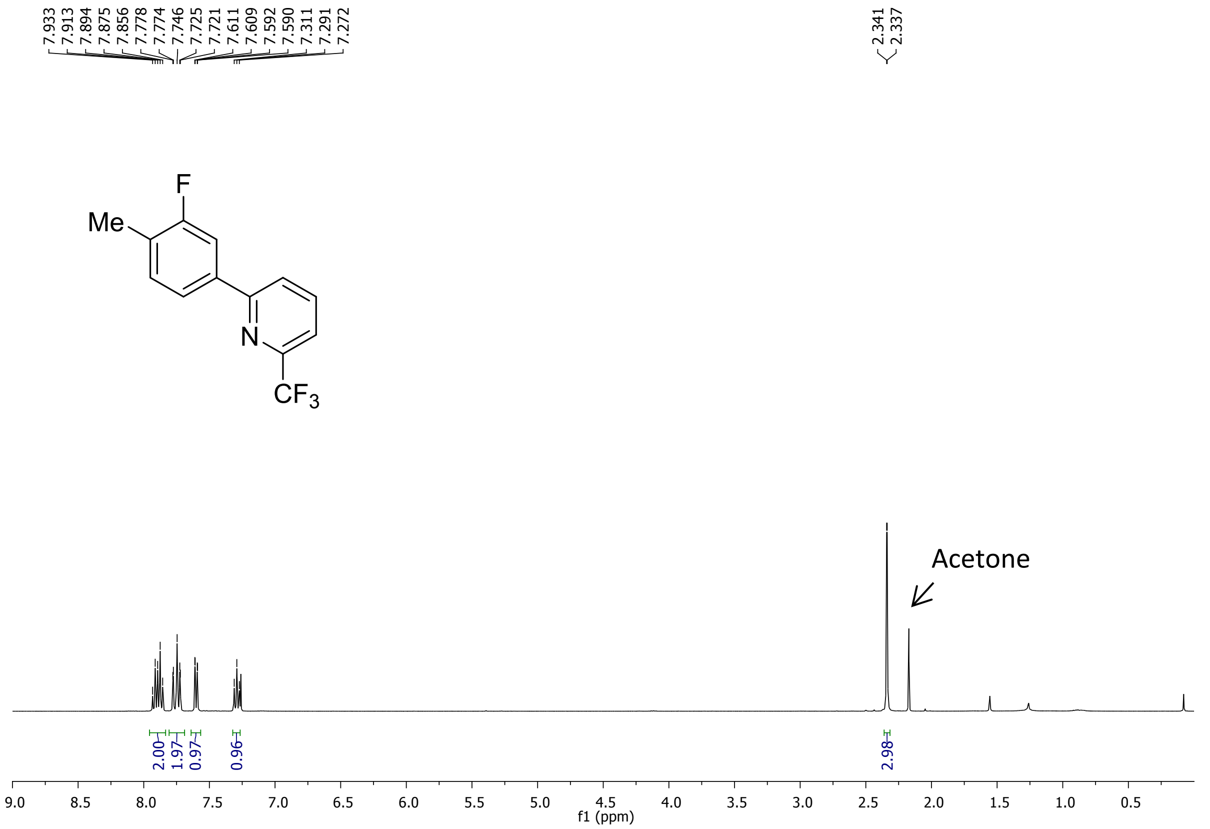

^{13}C NMR

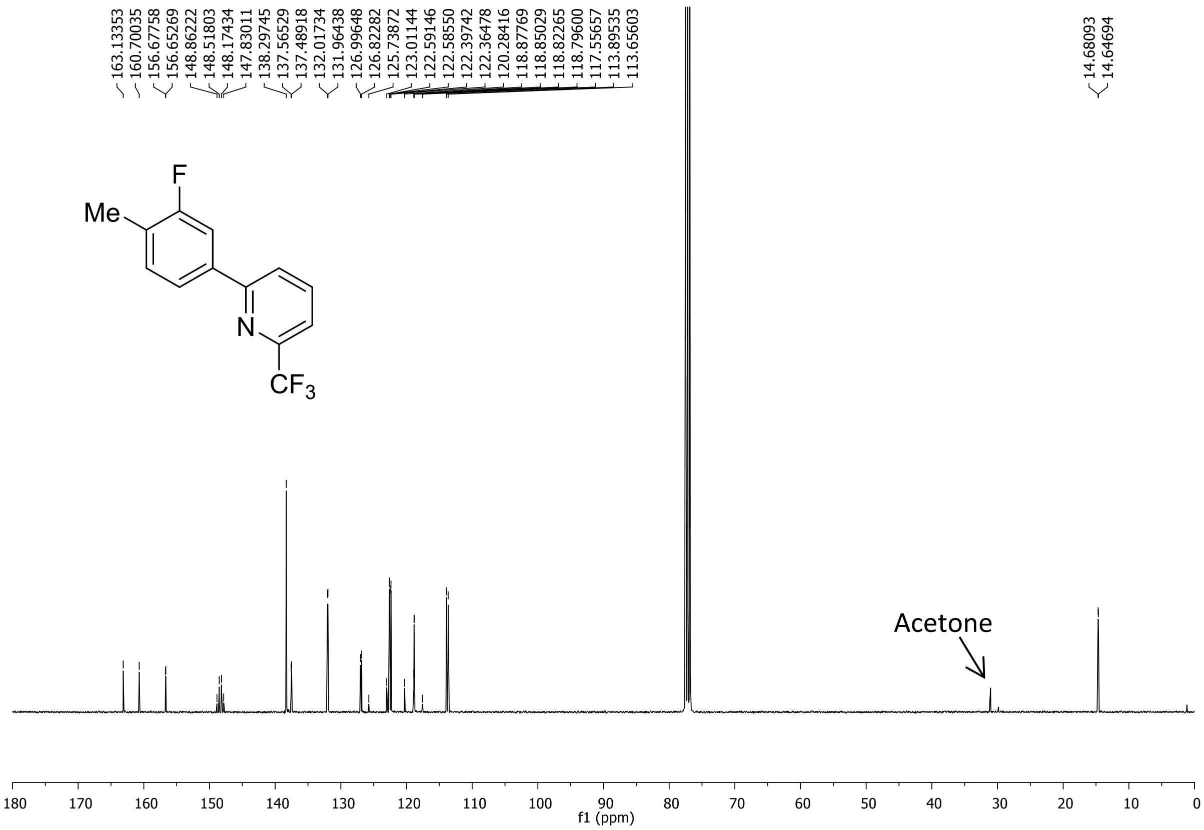

^{19}F NMR

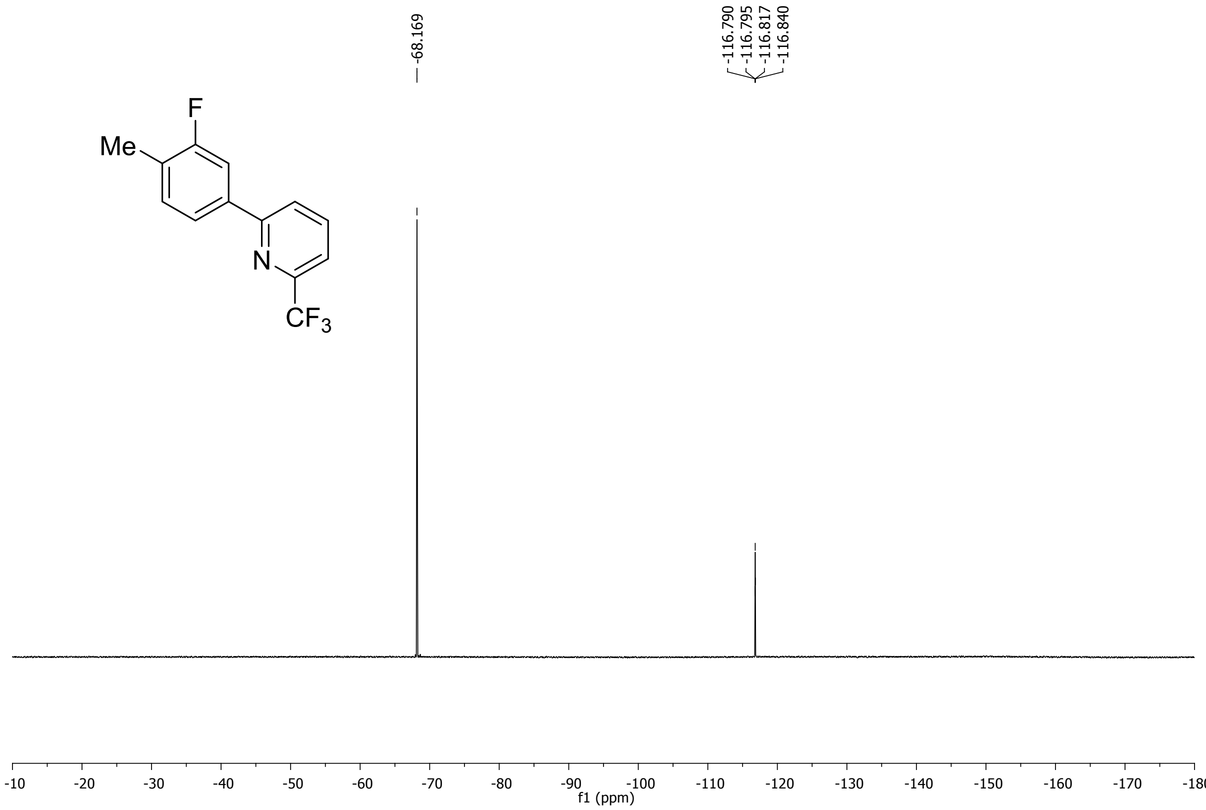

^{1}H NMR

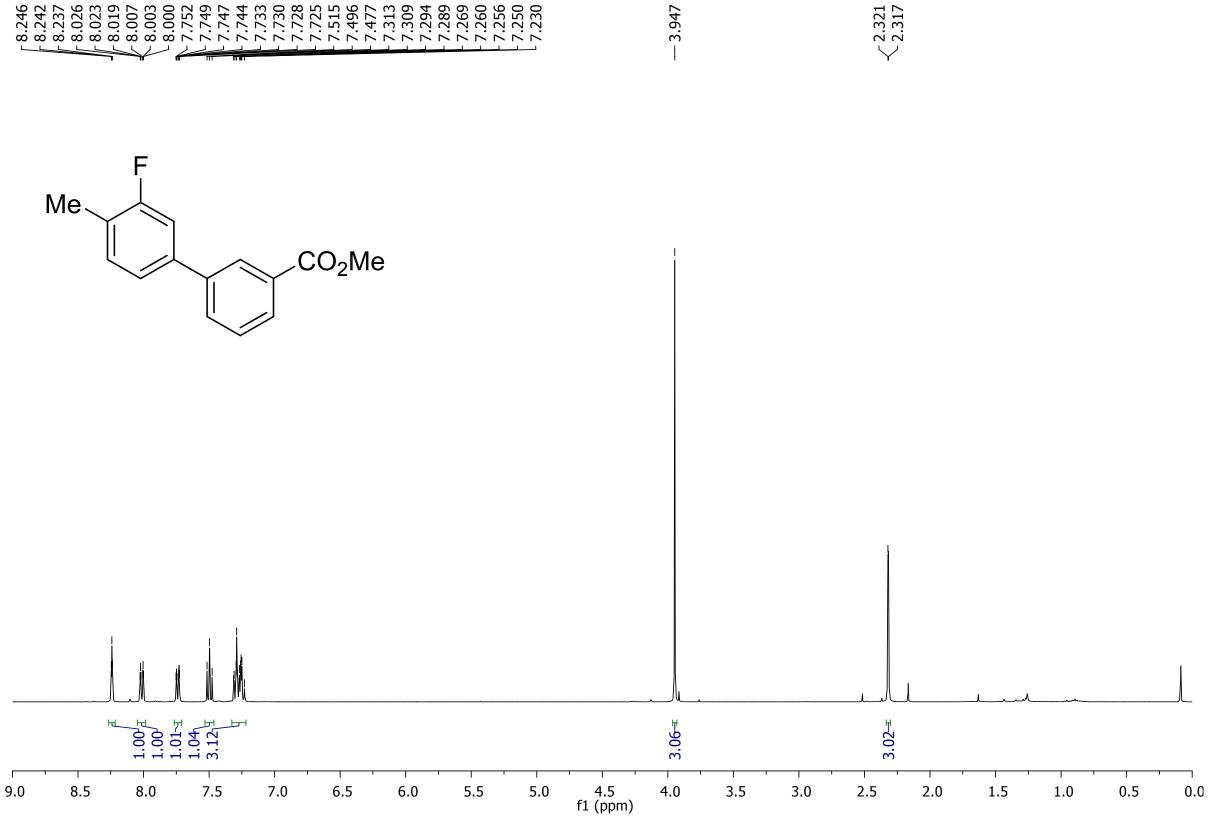

^{13}C NMR

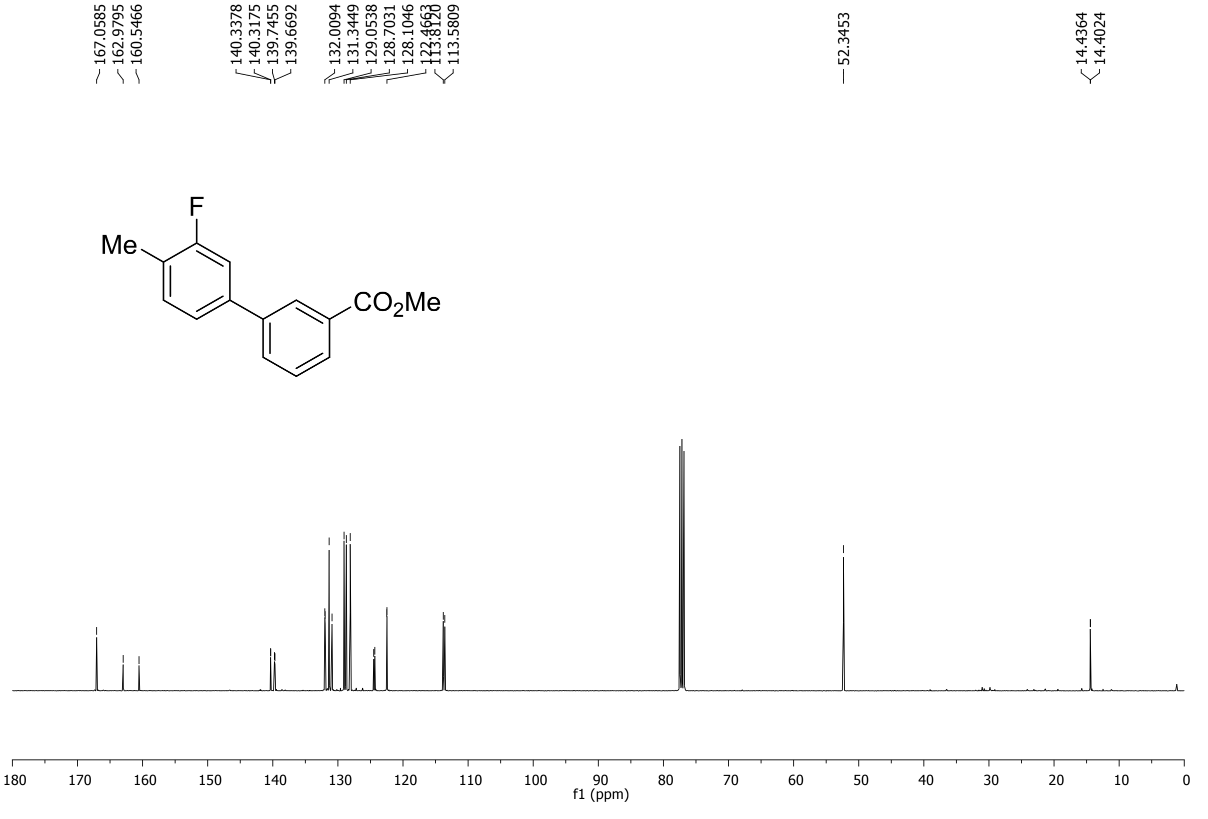

^{19}F NMR

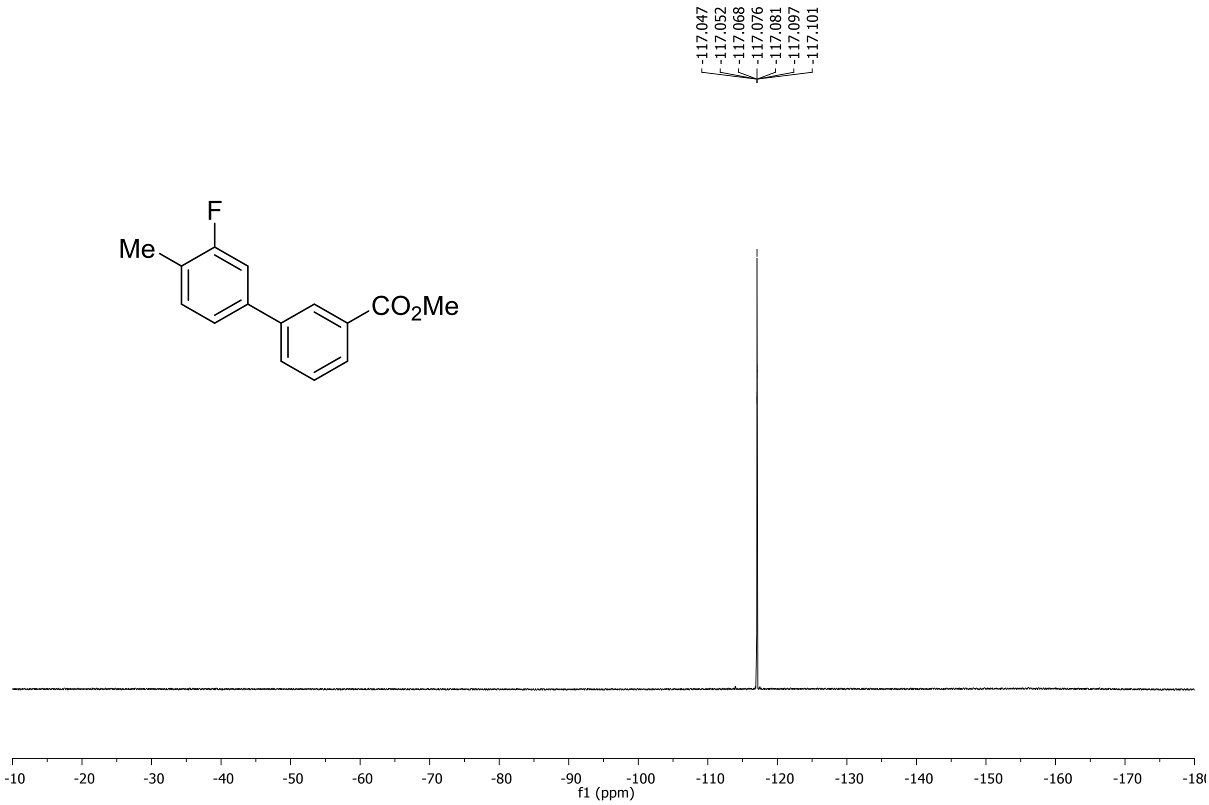

^{1}H NMR

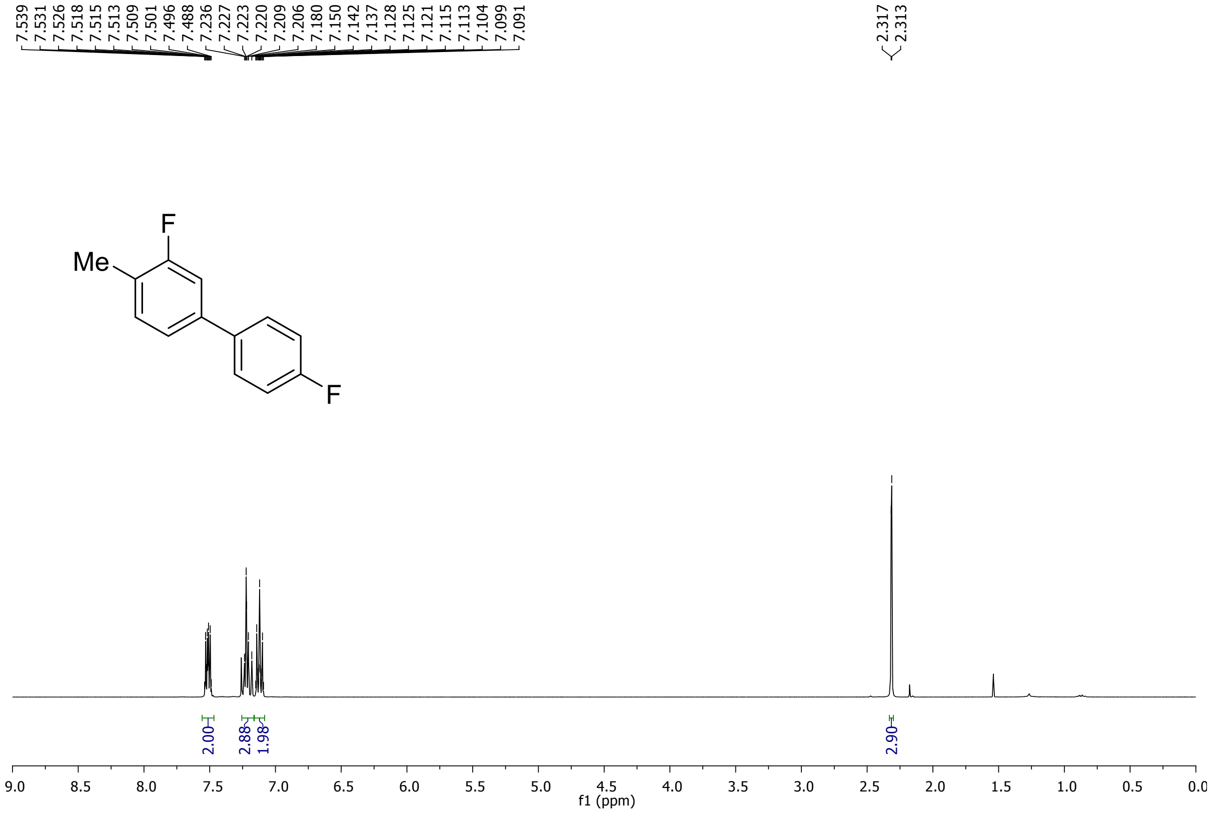

^{13}C NMR

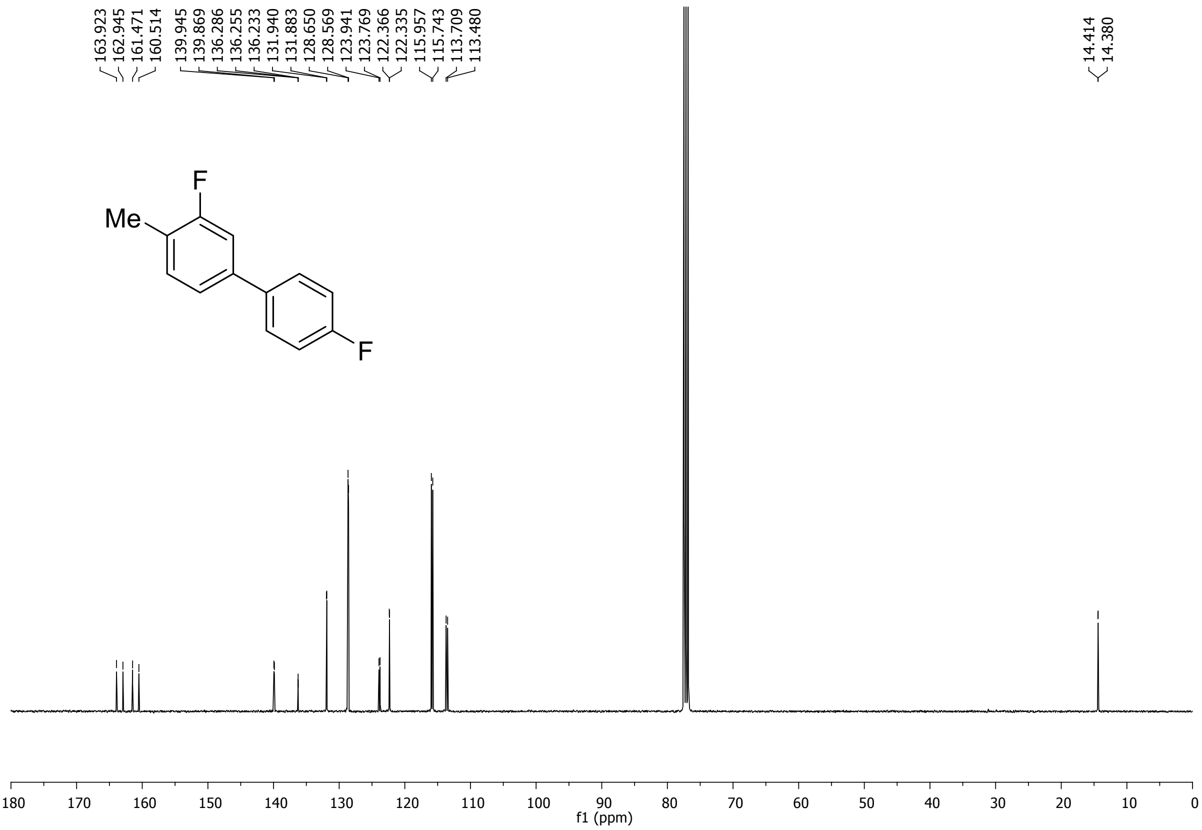

^{19}F NMR

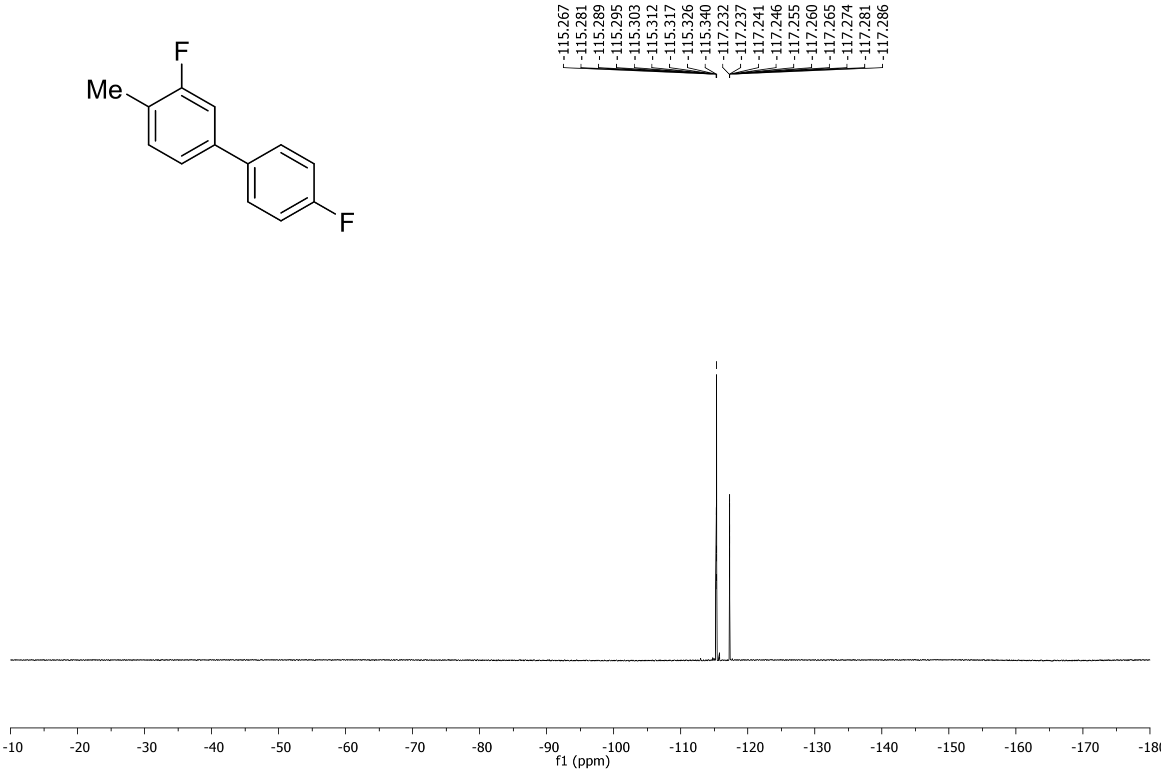

^{1}H NMR

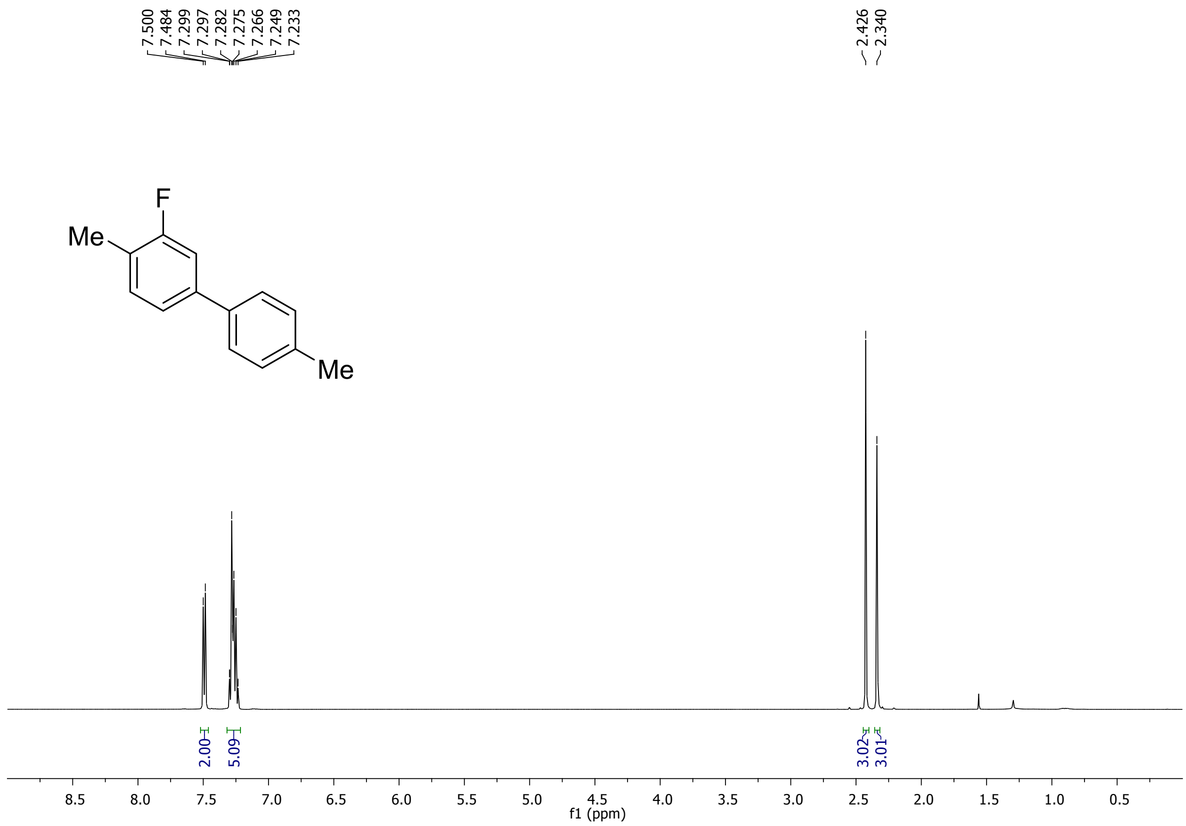

^{13}C NMR

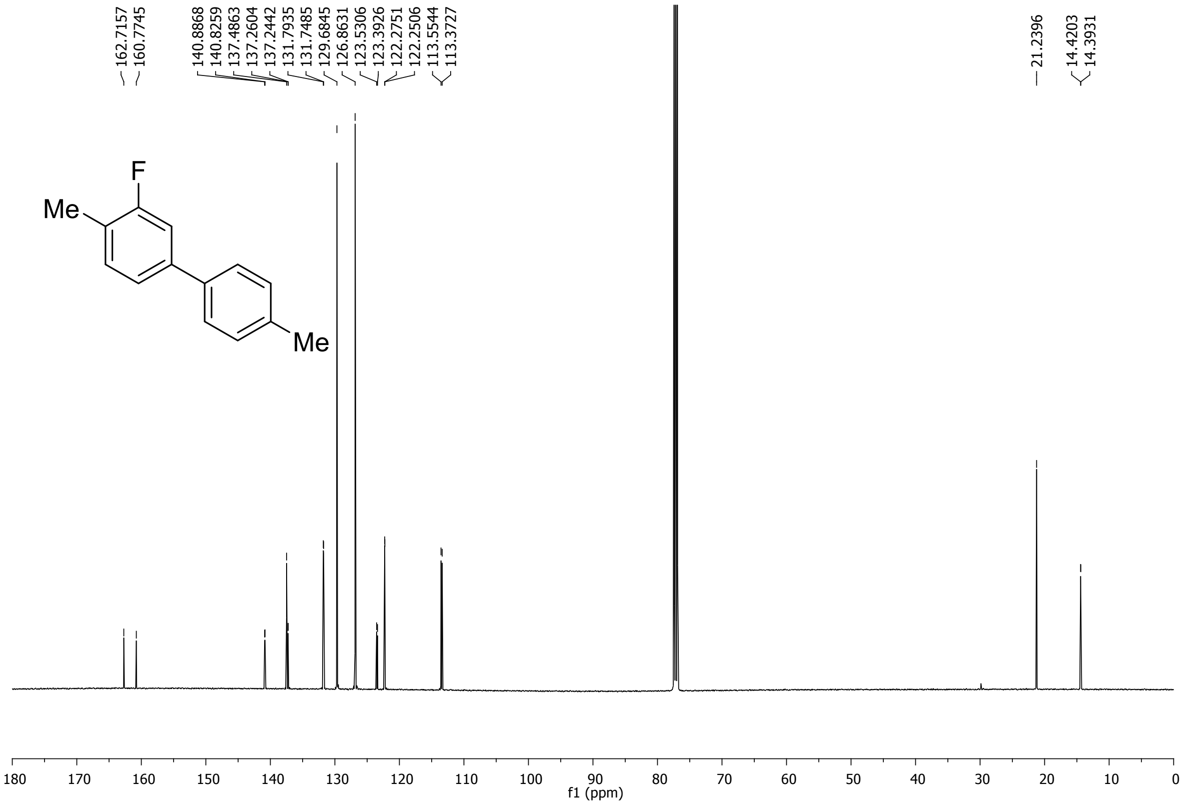

^{19}F NMR

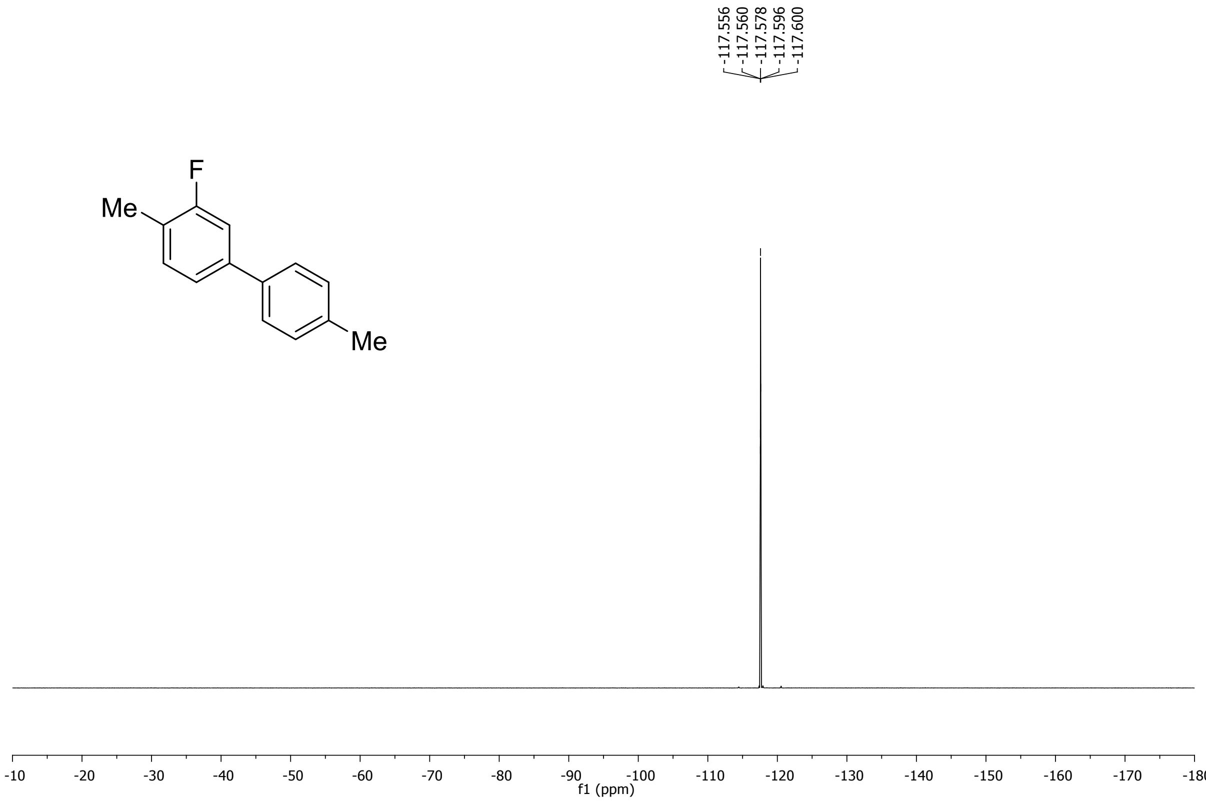

^{1}H NMR

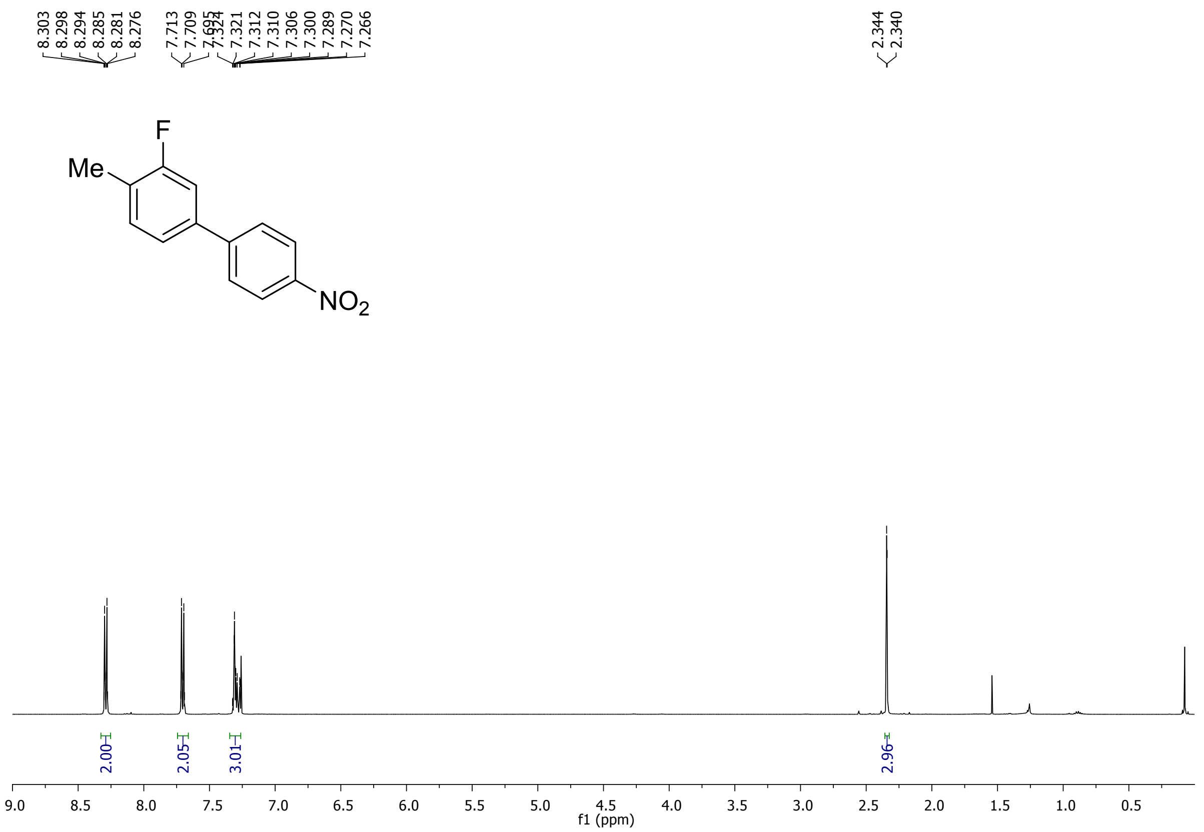

^{13}C NMR

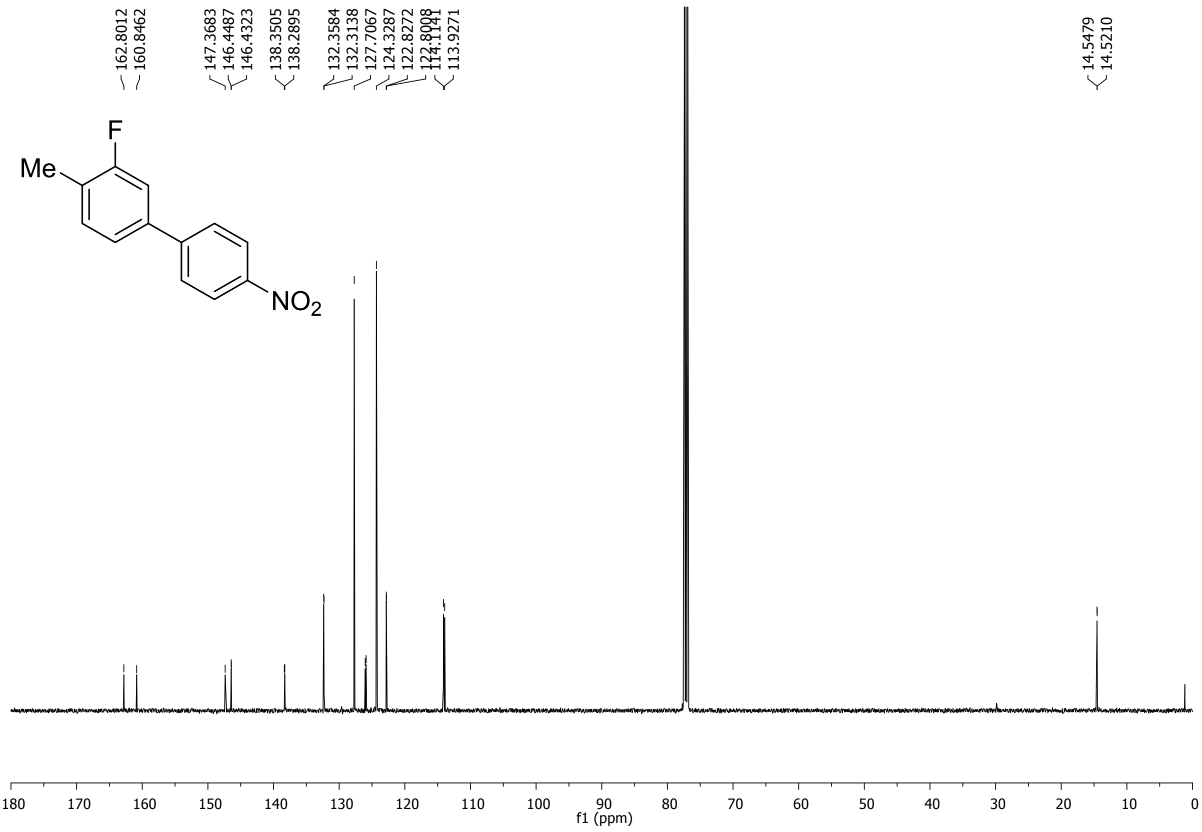

^{19}F NMR

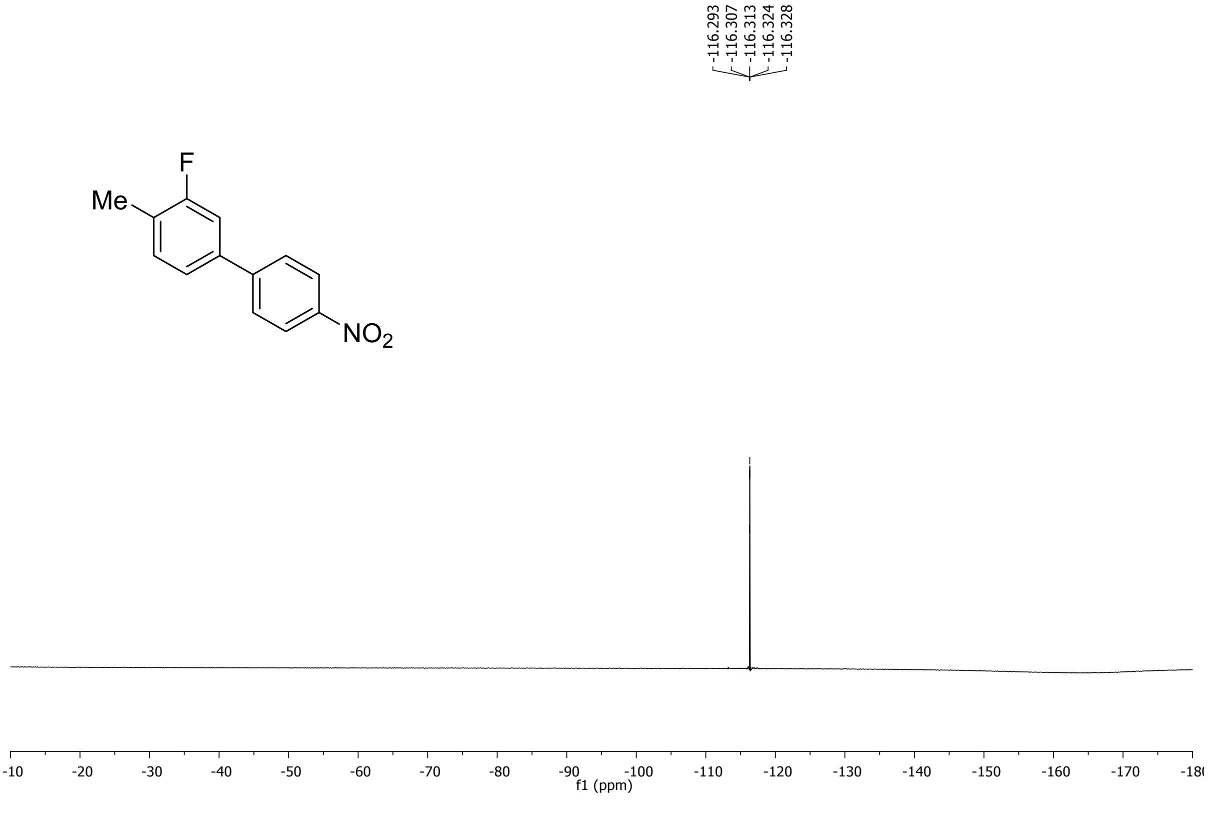

^{1}H NMR

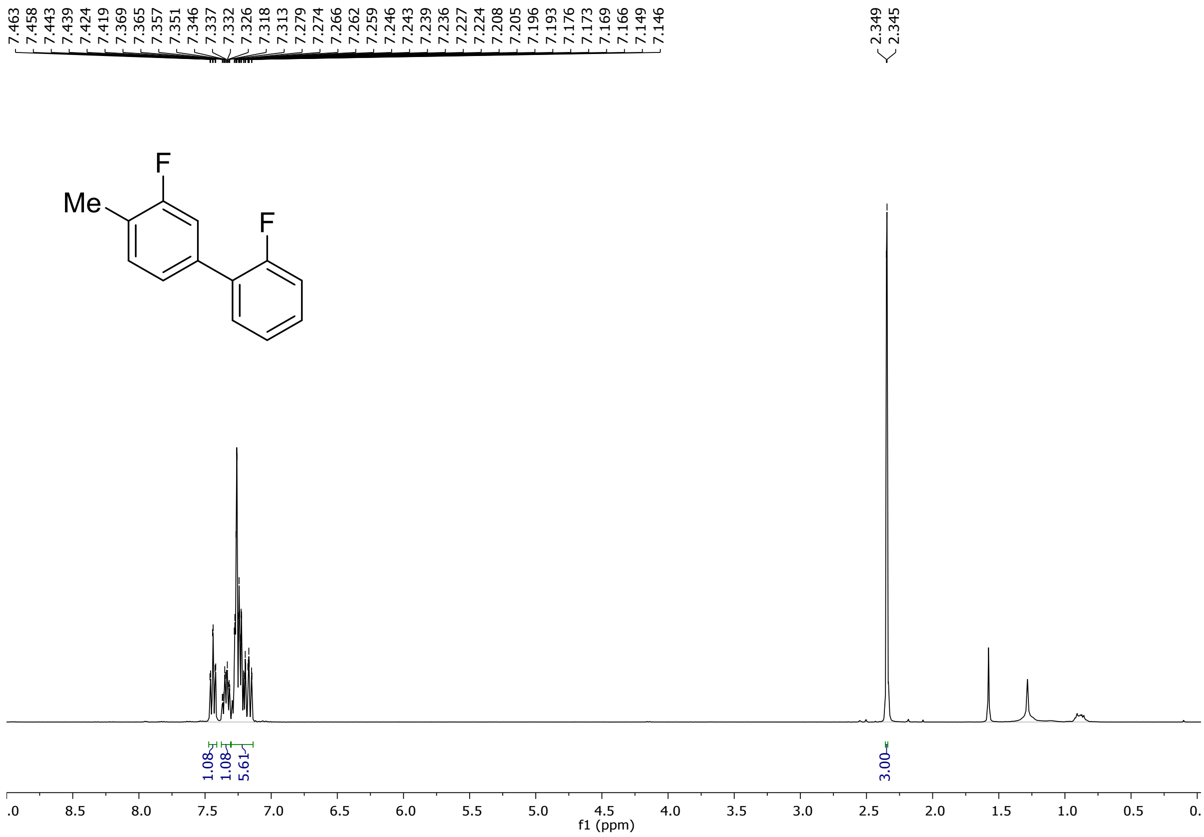

^{13}C NMR

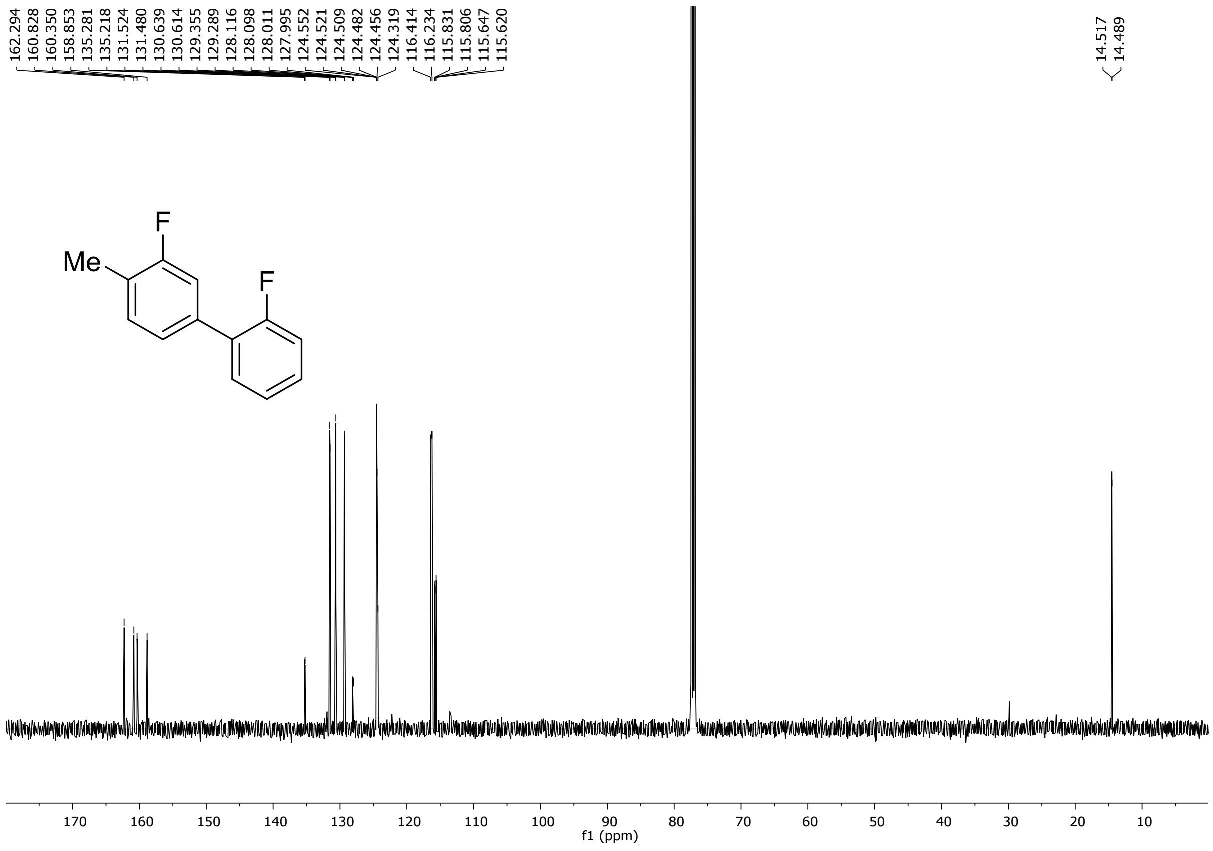

^{19}F NMR

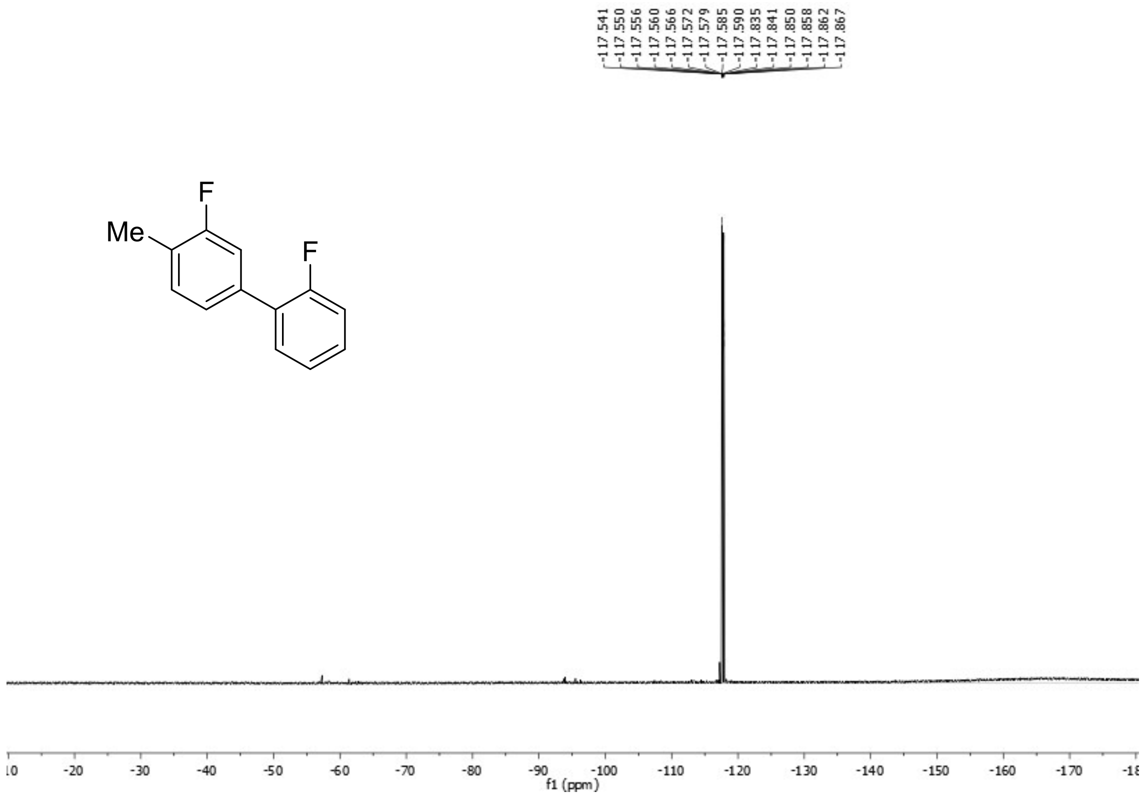

^{1}H NMR

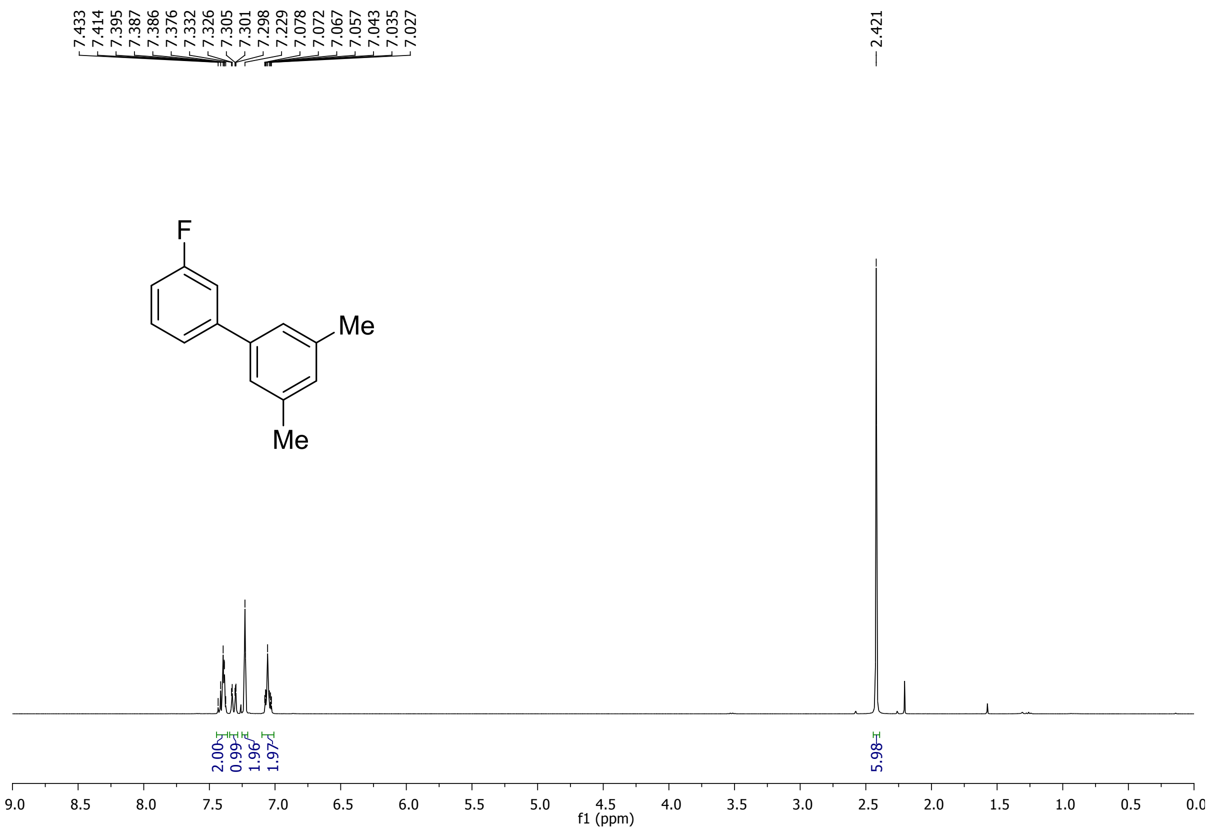

^{13}C NMR

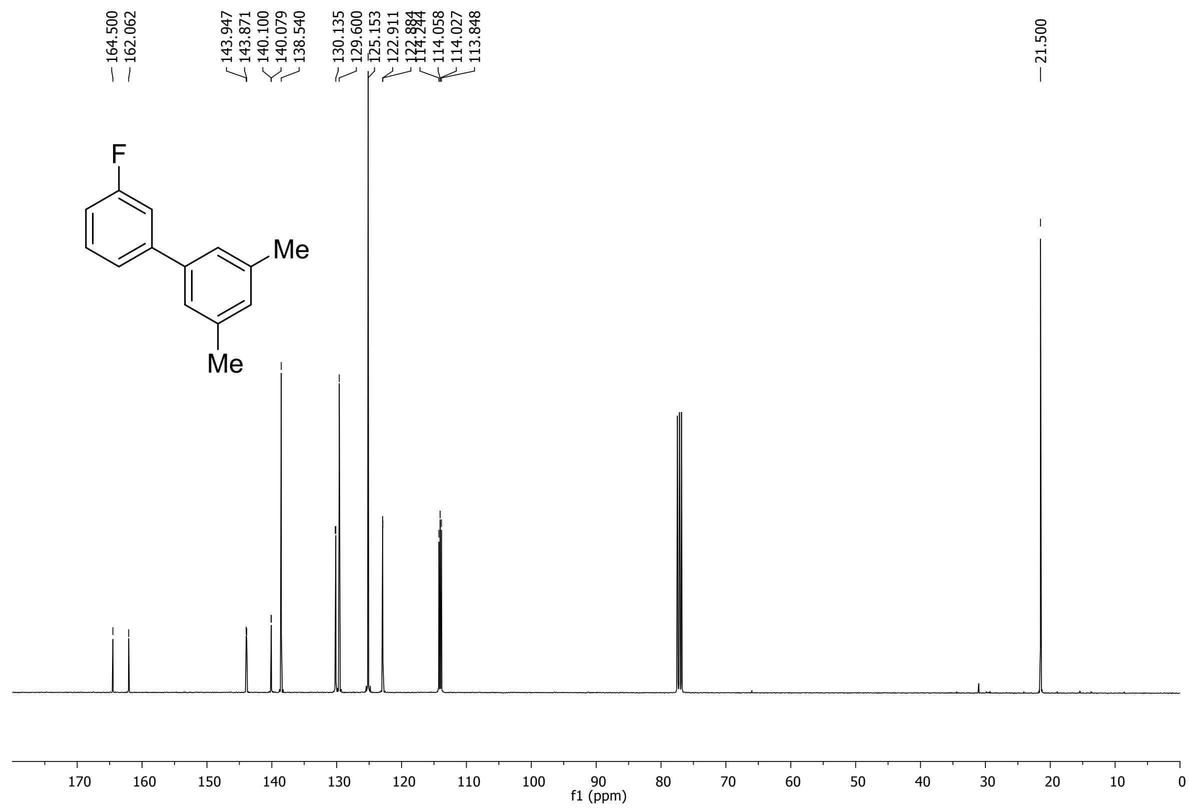

^{19}F NMR

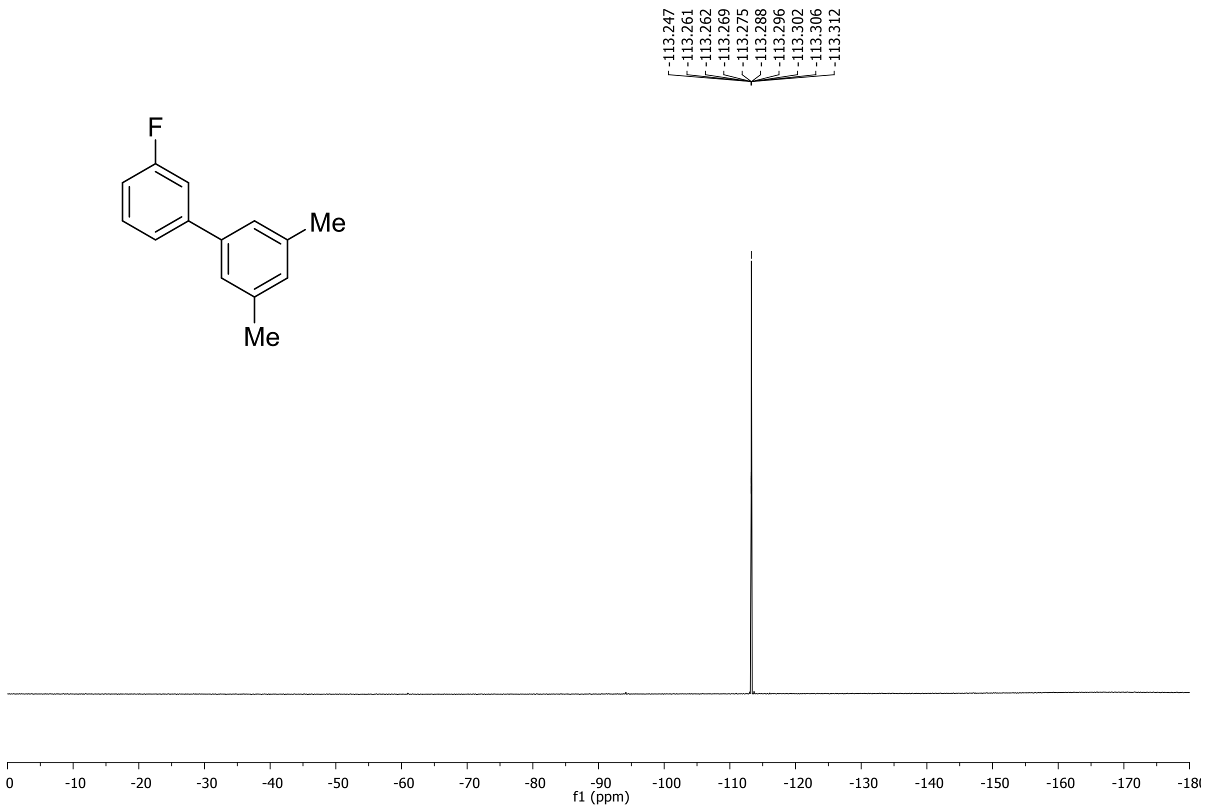

^{1}H NMR

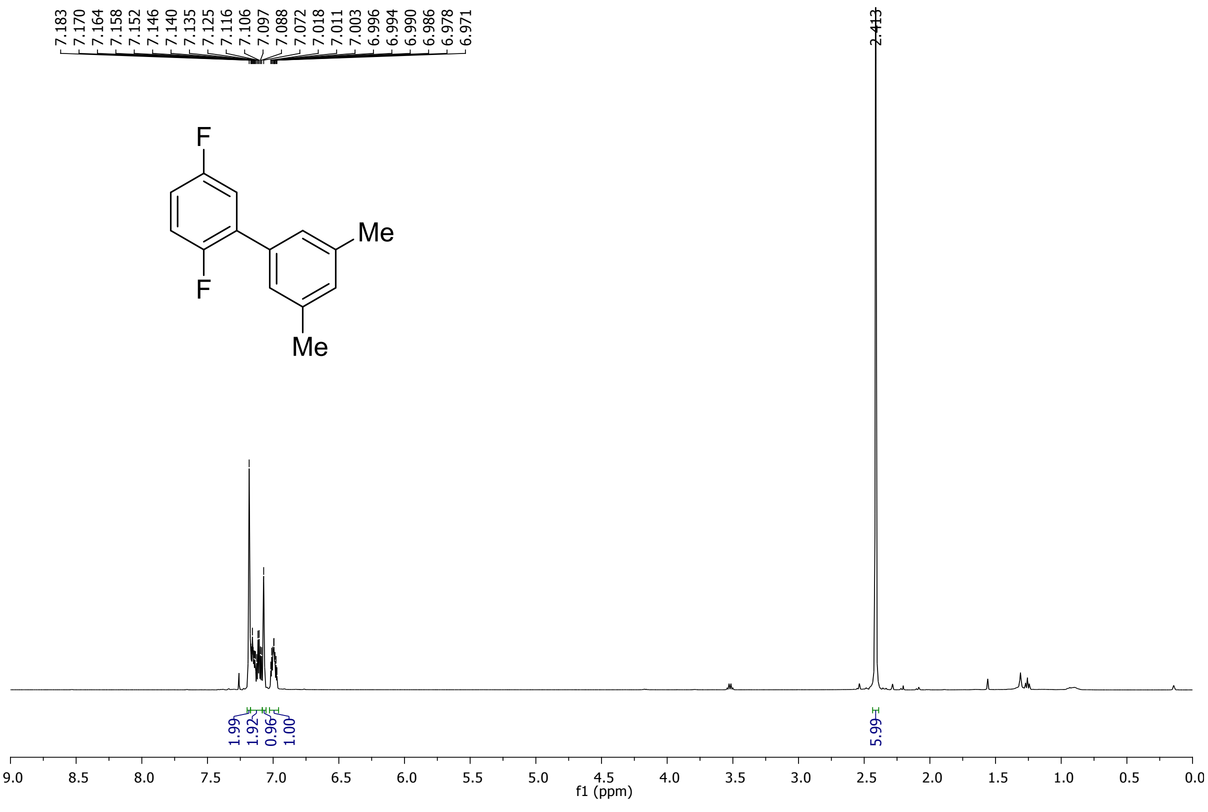

^{13}C NMR

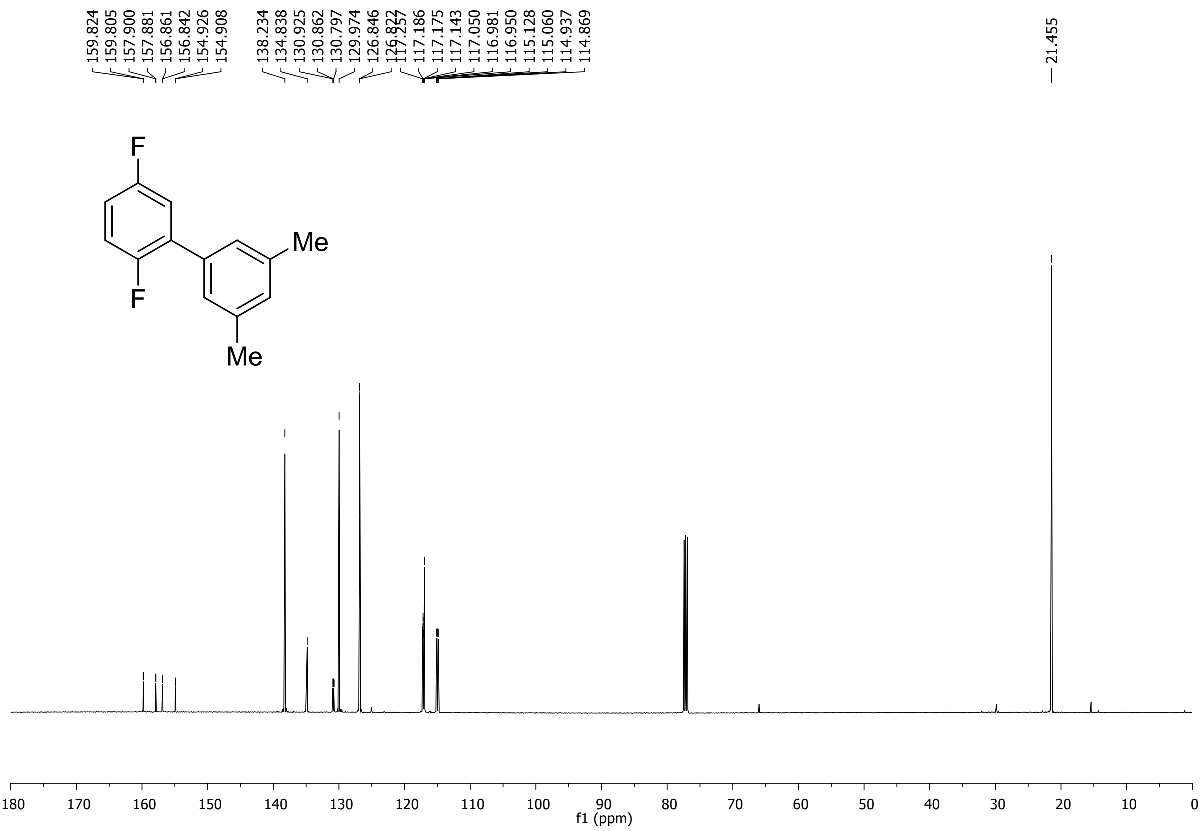

^{19}F NMR

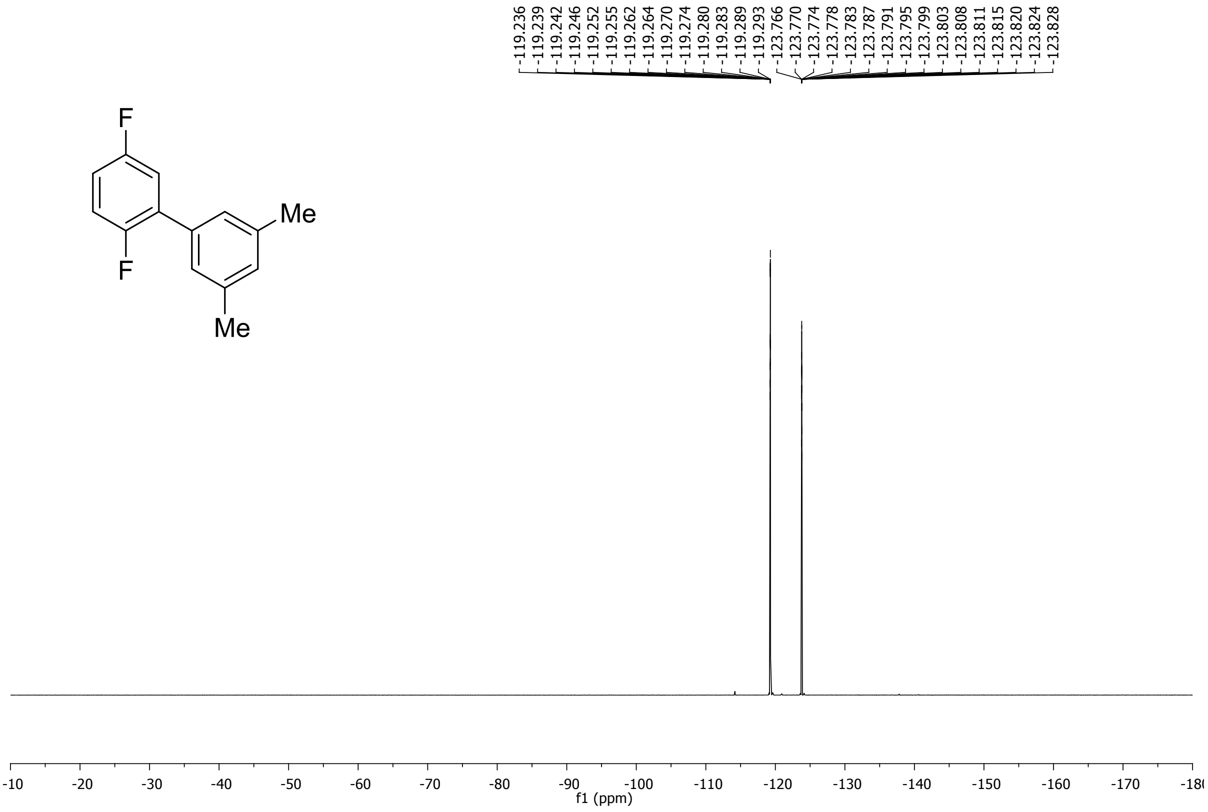

^{1}H NMR

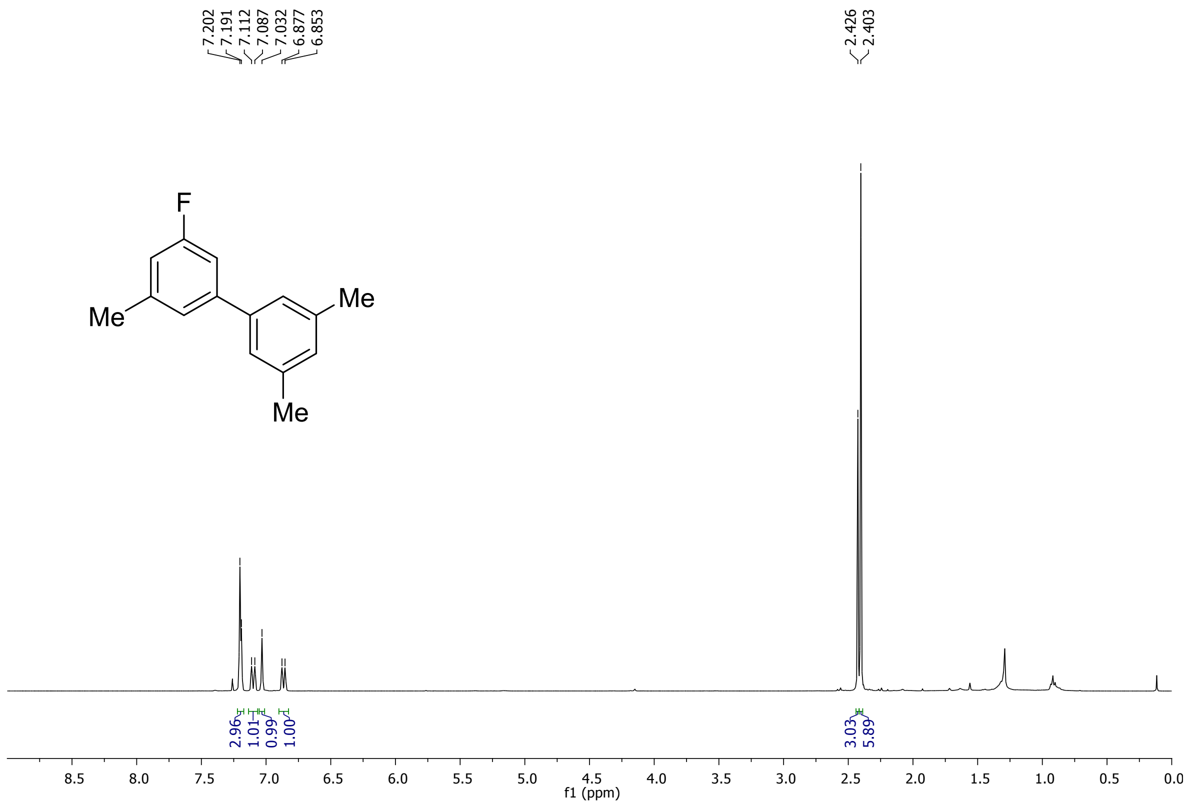

^{13}C NMR

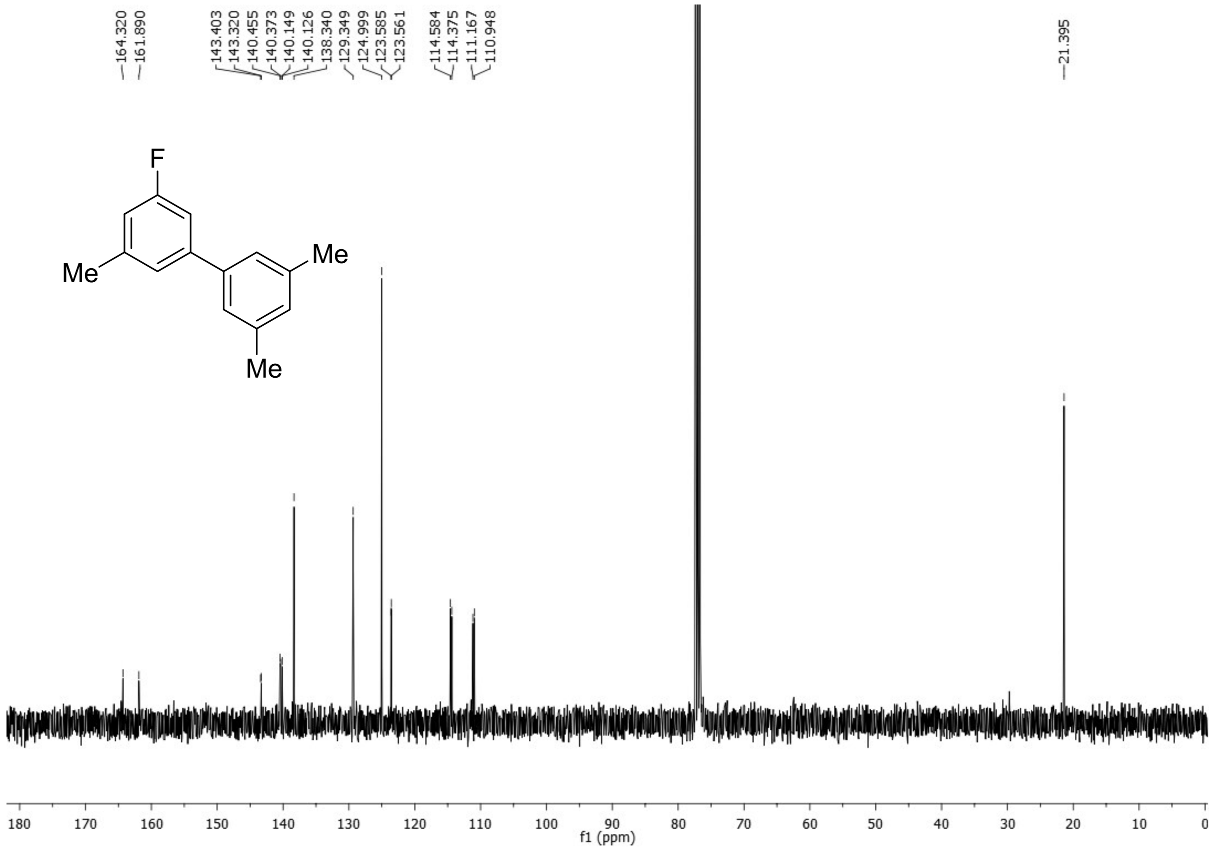

^{19}F NMR

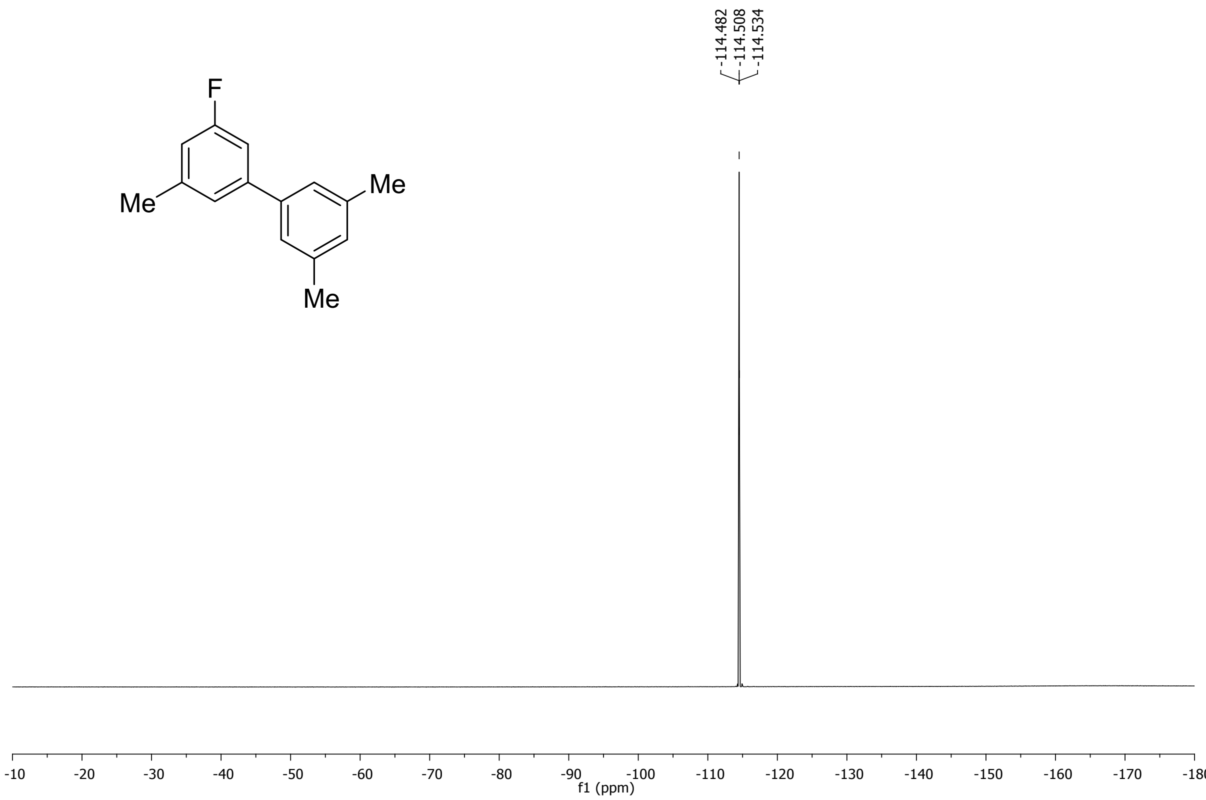

^{1}H NMR

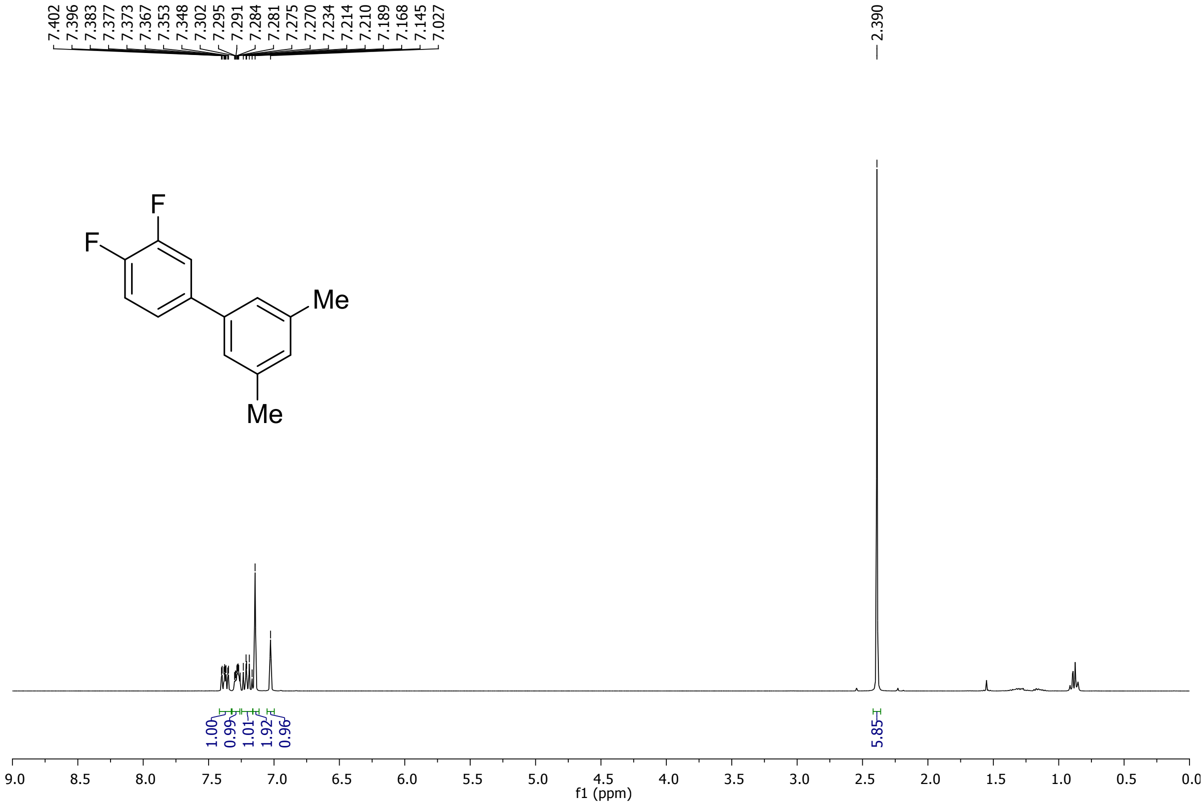

^{13}C NMR

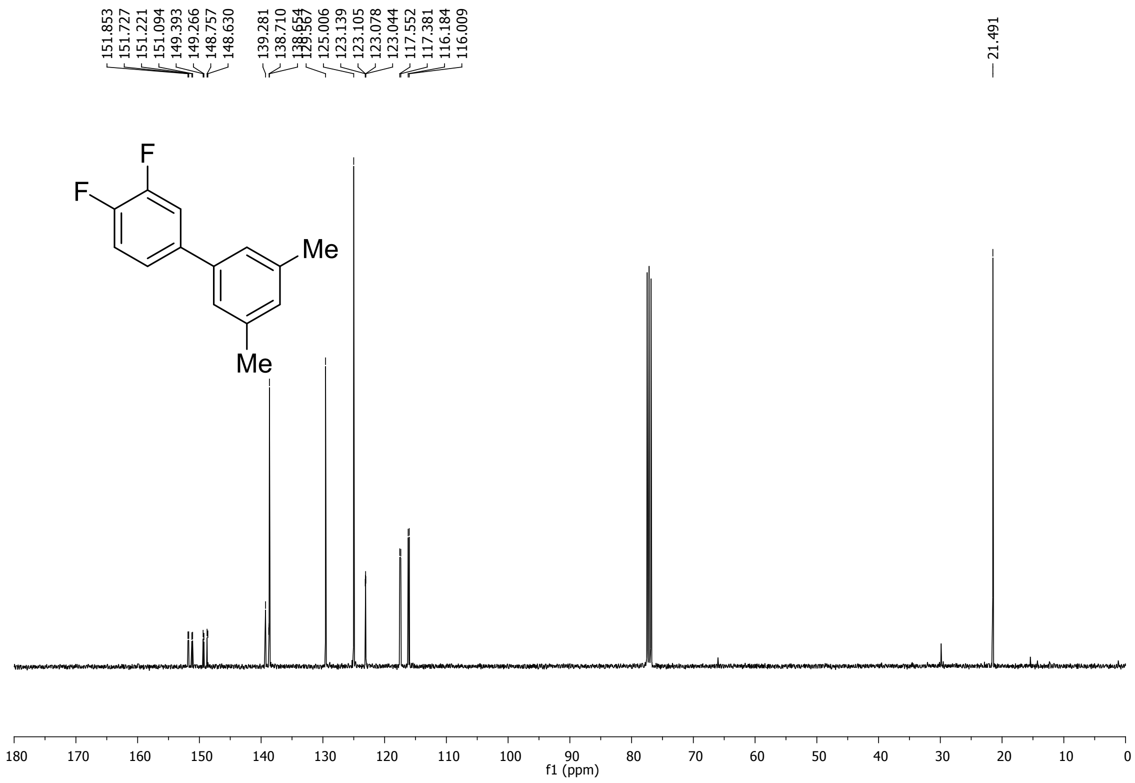

^{19}F NMR

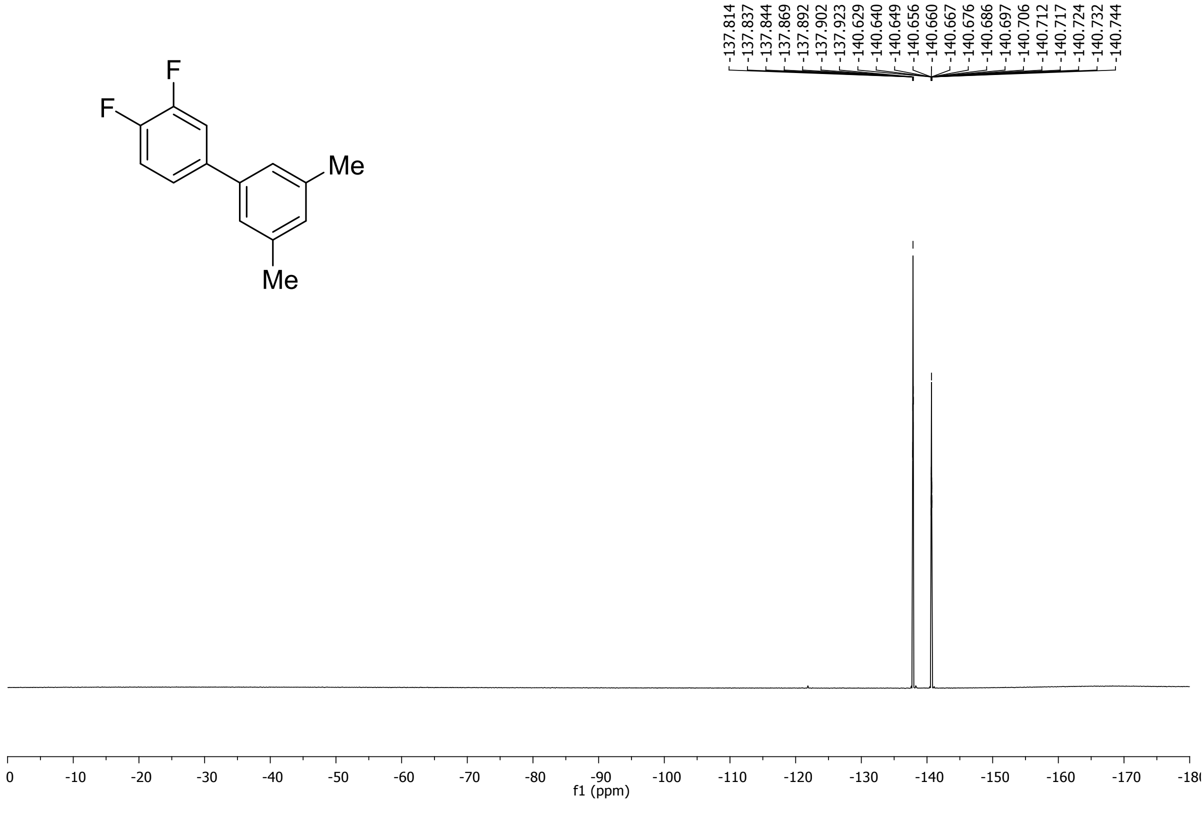

^{1}H NMR

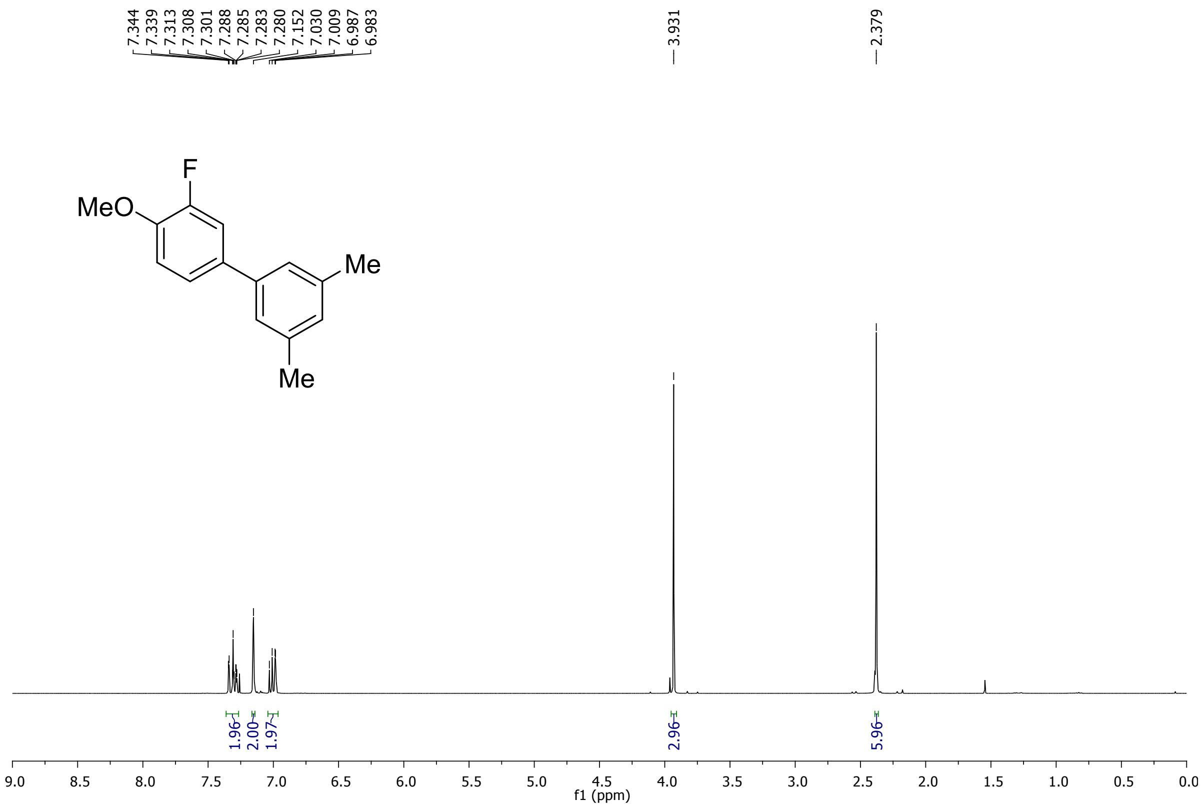

^{13}C NMR

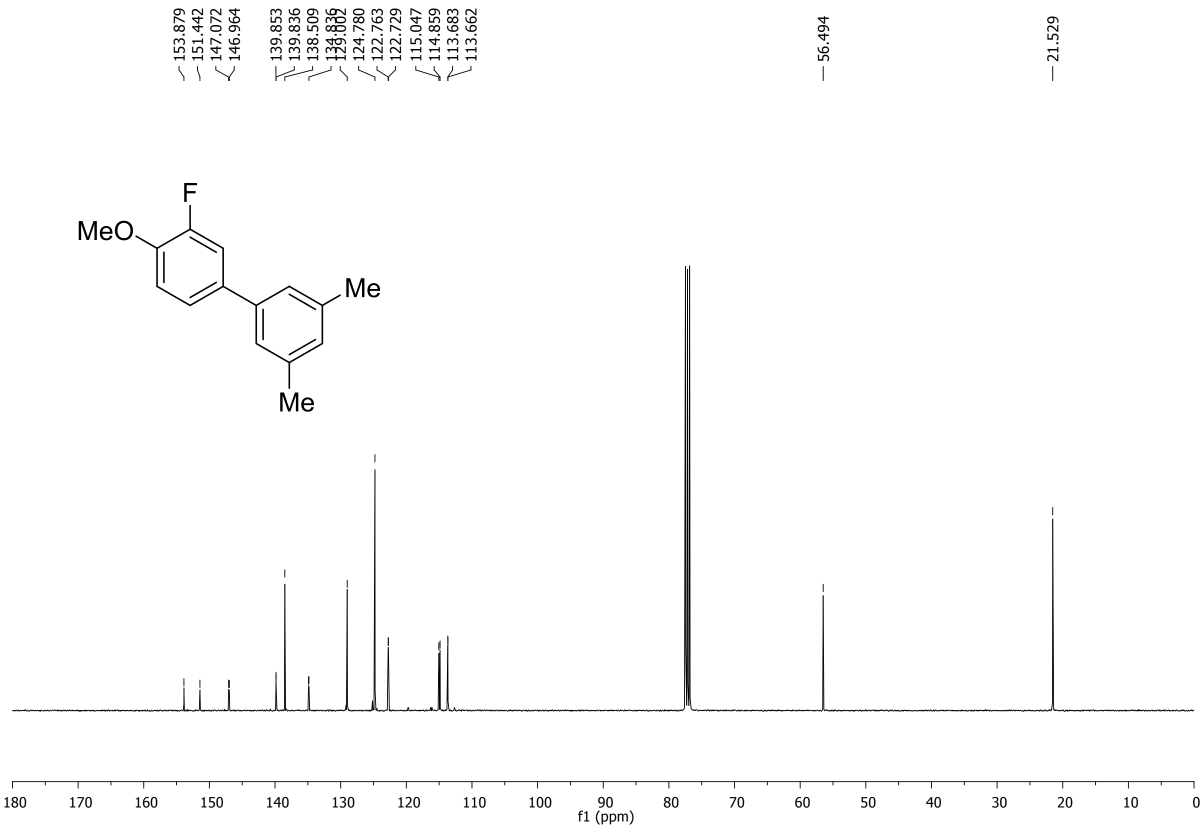

^{19}F NMR

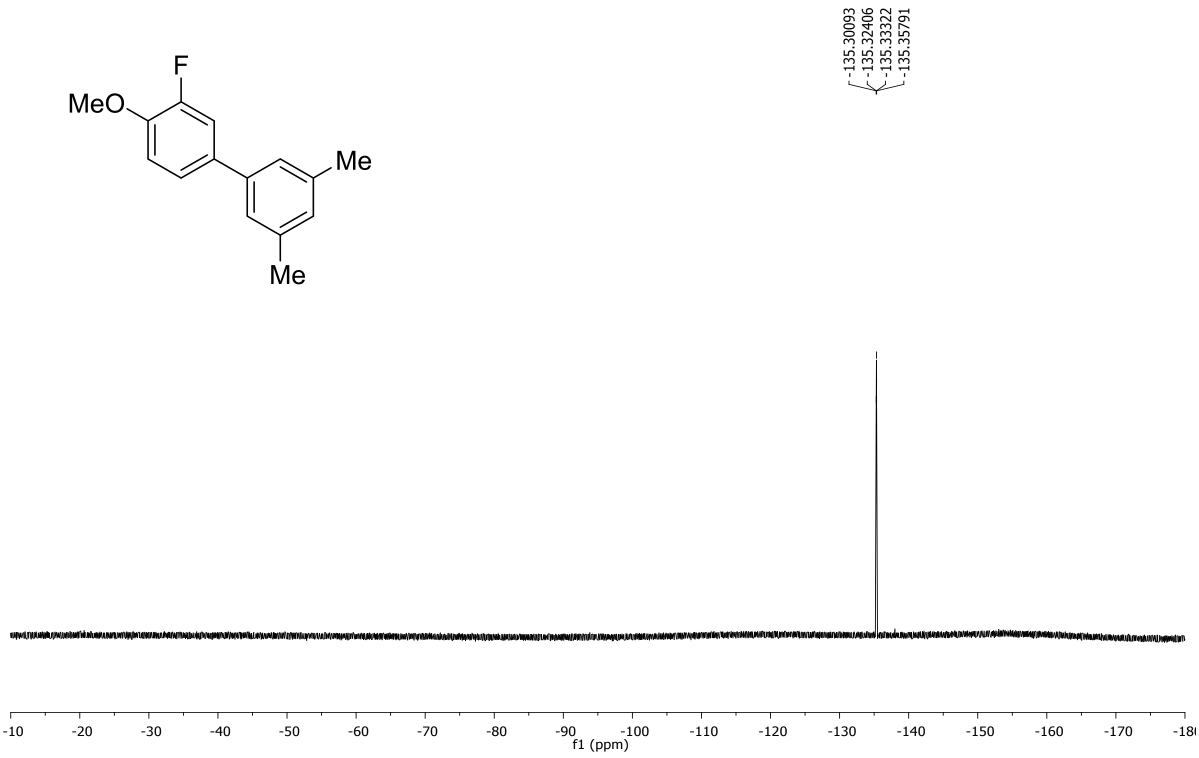

^{1}H ^{1}H COSY

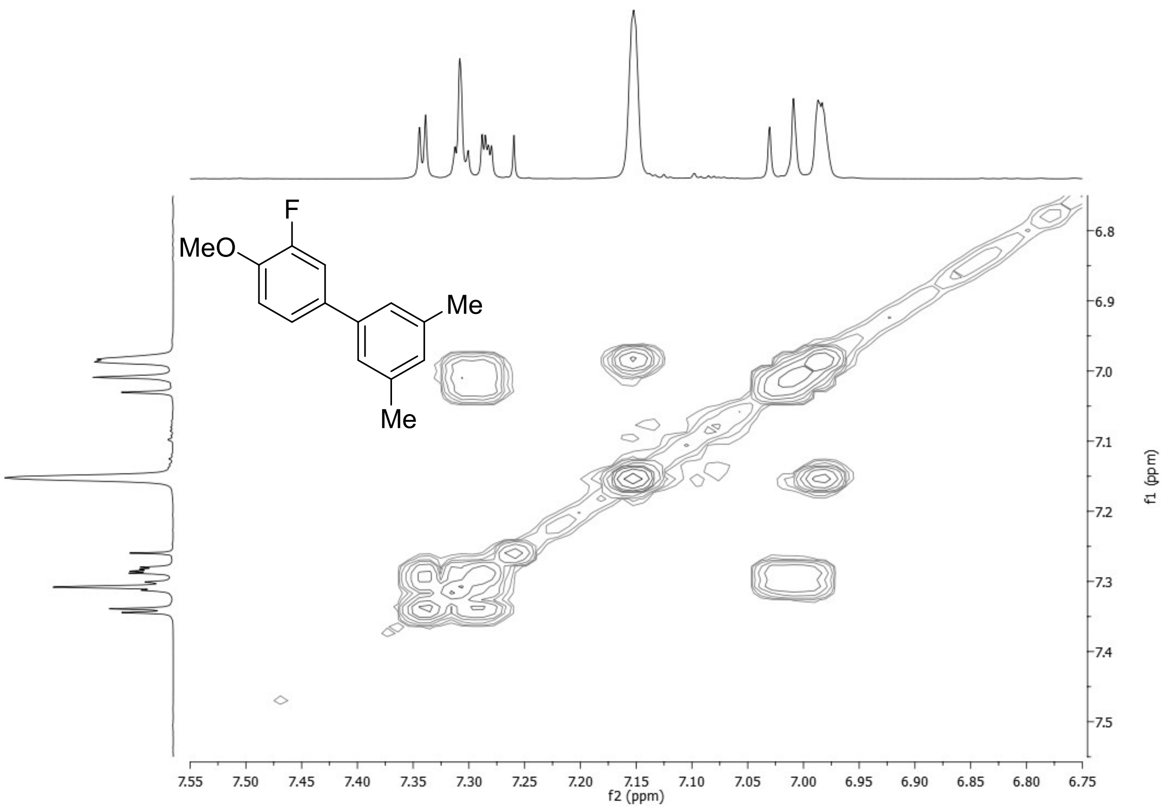

^{1}H ^{13}C HSQC

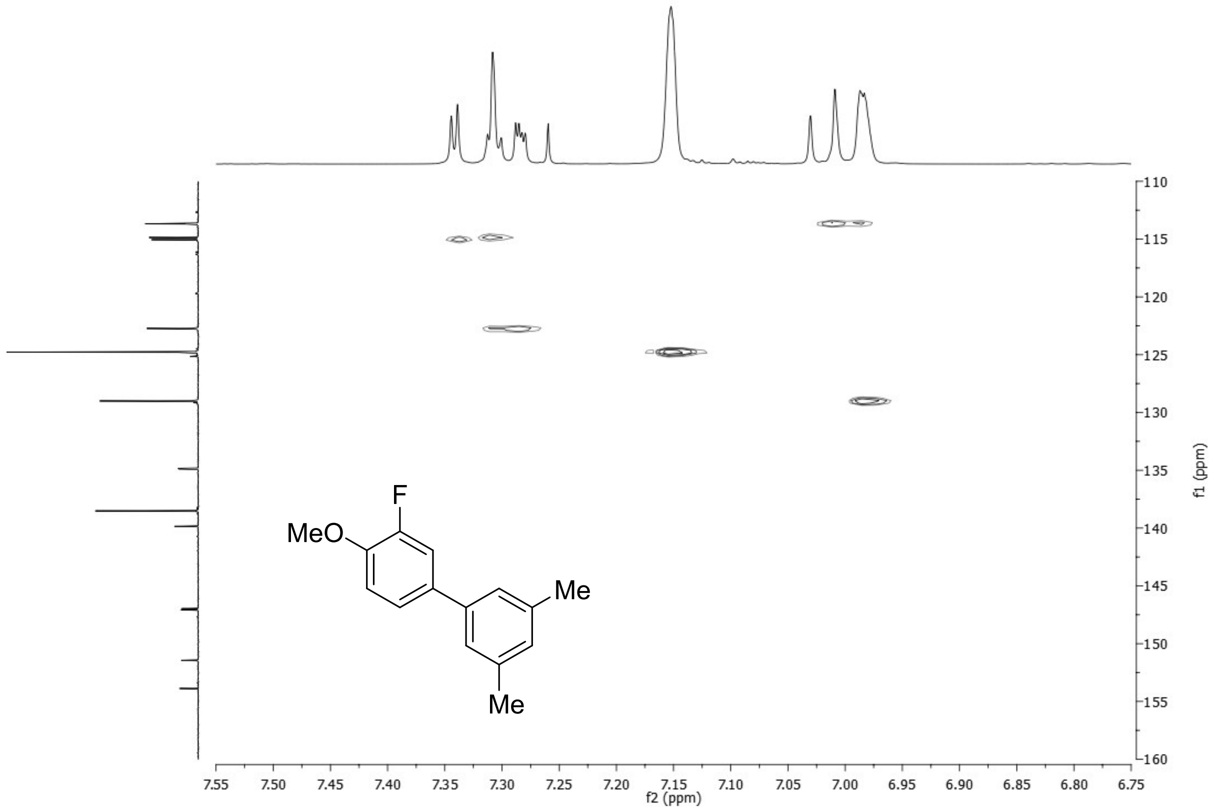
MeO
F
Me
Me
f2 (ppm)
f1 (ppm)

^{1}H NMR

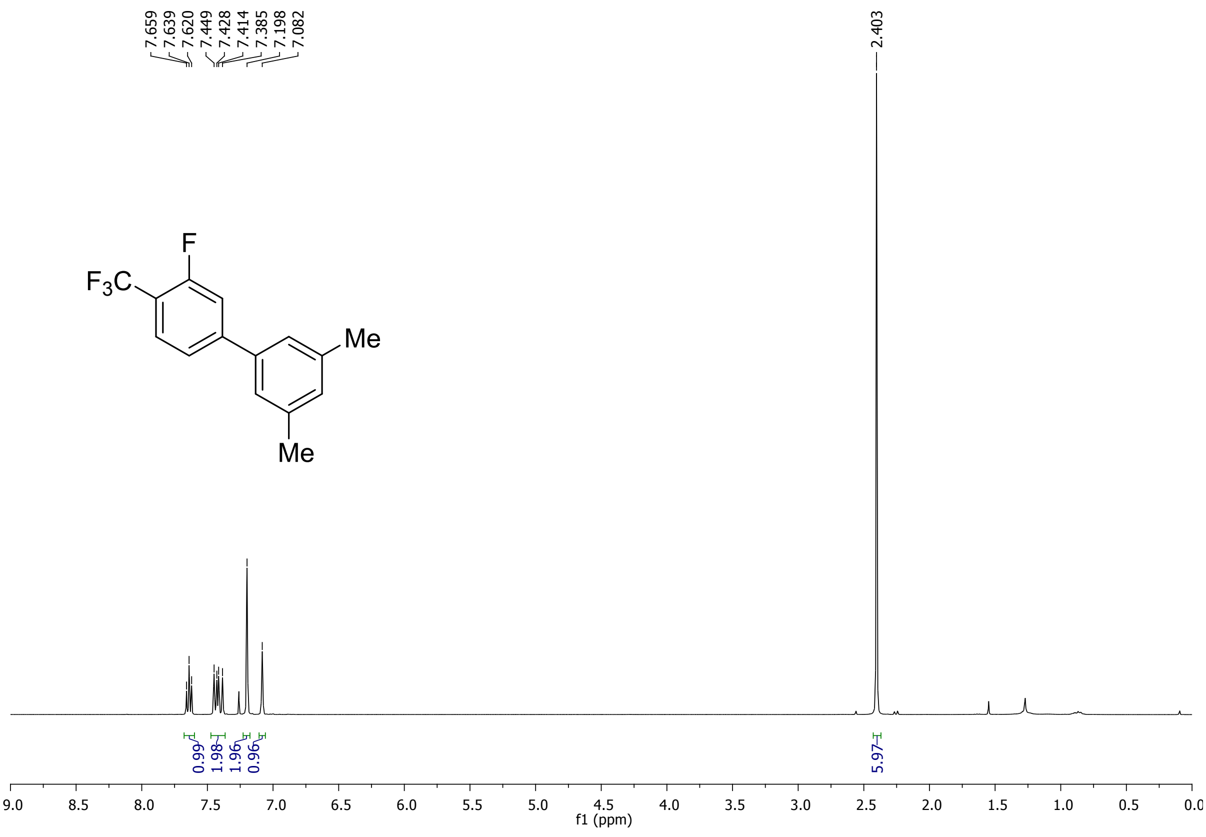
7.659
7.639
7.620
7.449
7.428
7.414
7.385
7.198
7.082
2.403
F
F3C
Me
Me
0.99
1.98
1.96
0.96
5.97
f1 (ppm)

^{13}C NMR

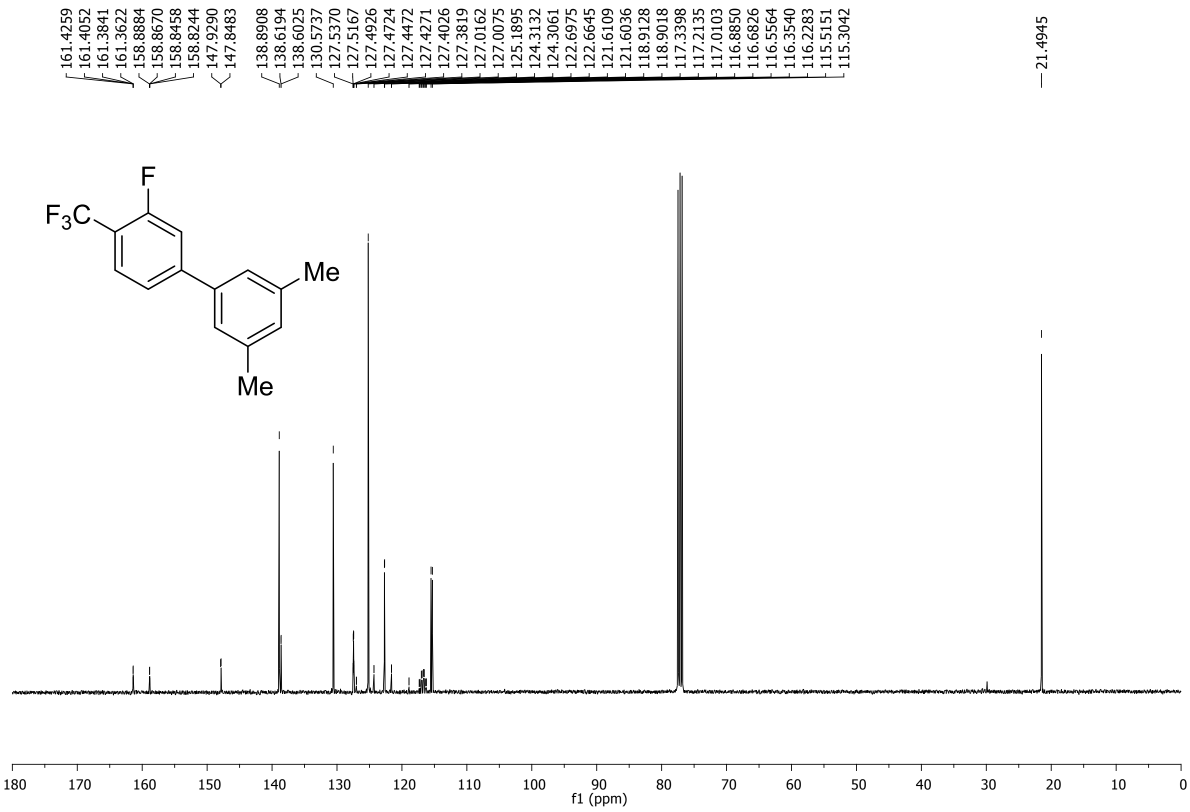

^{19}F NMR

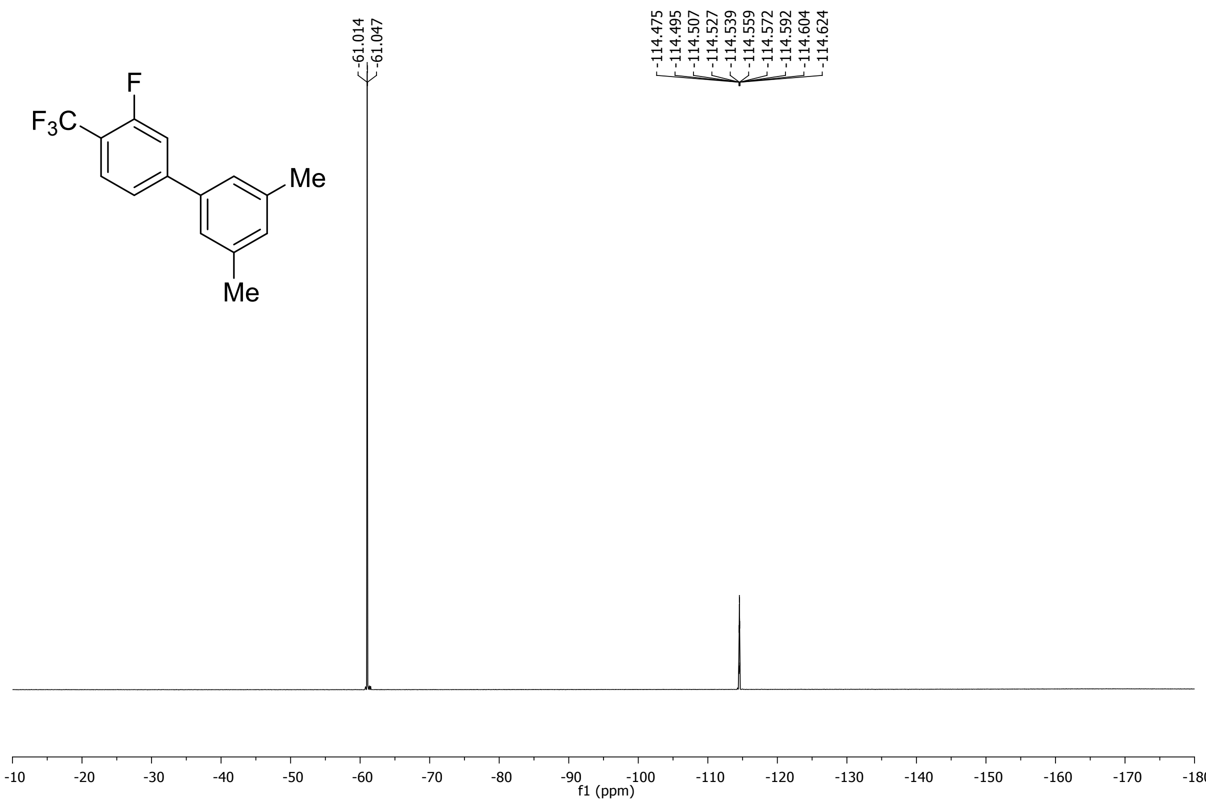

^{1}H NMR

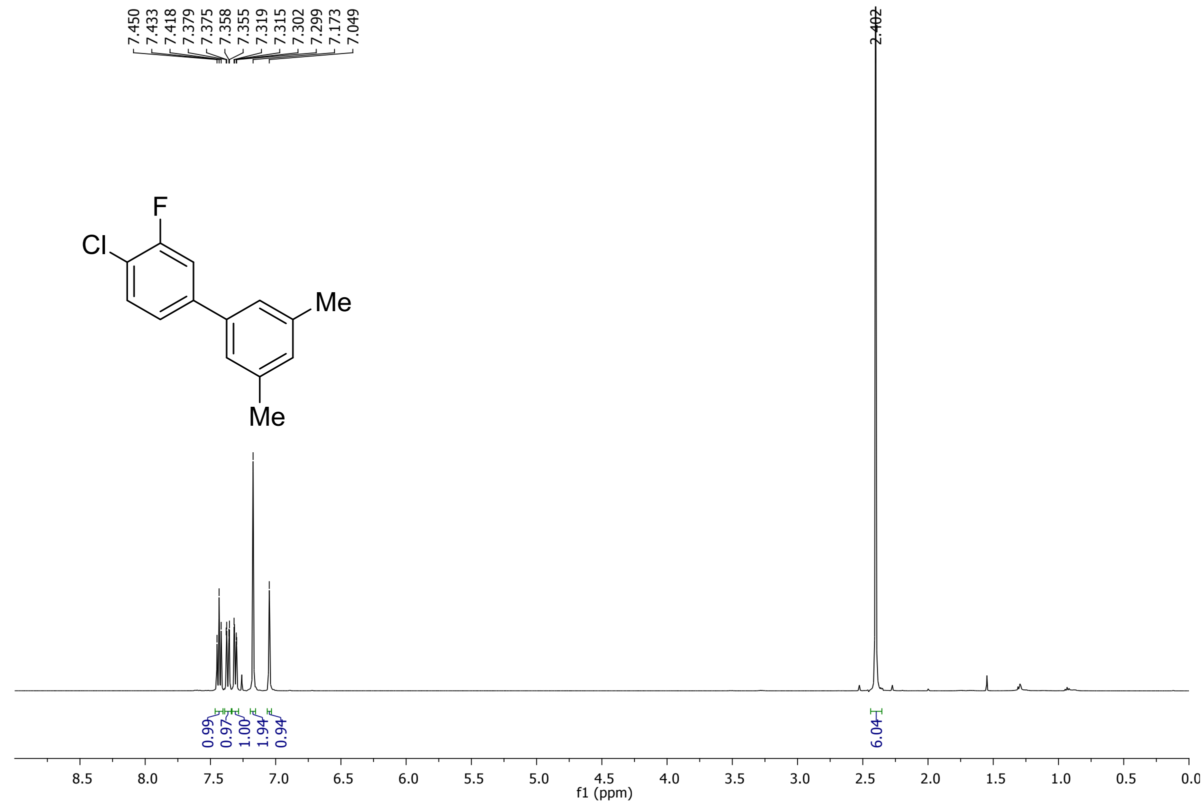

^{13}C NMR

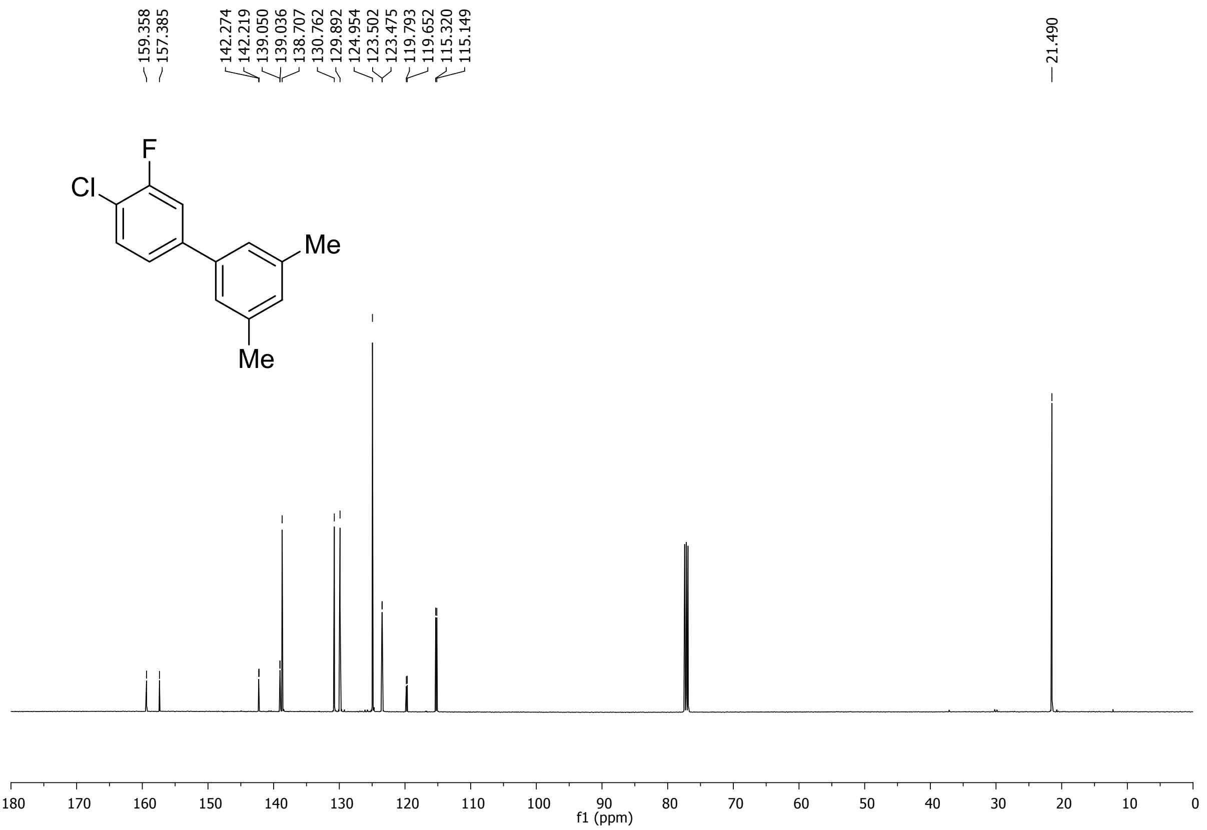

^{19}F NMR

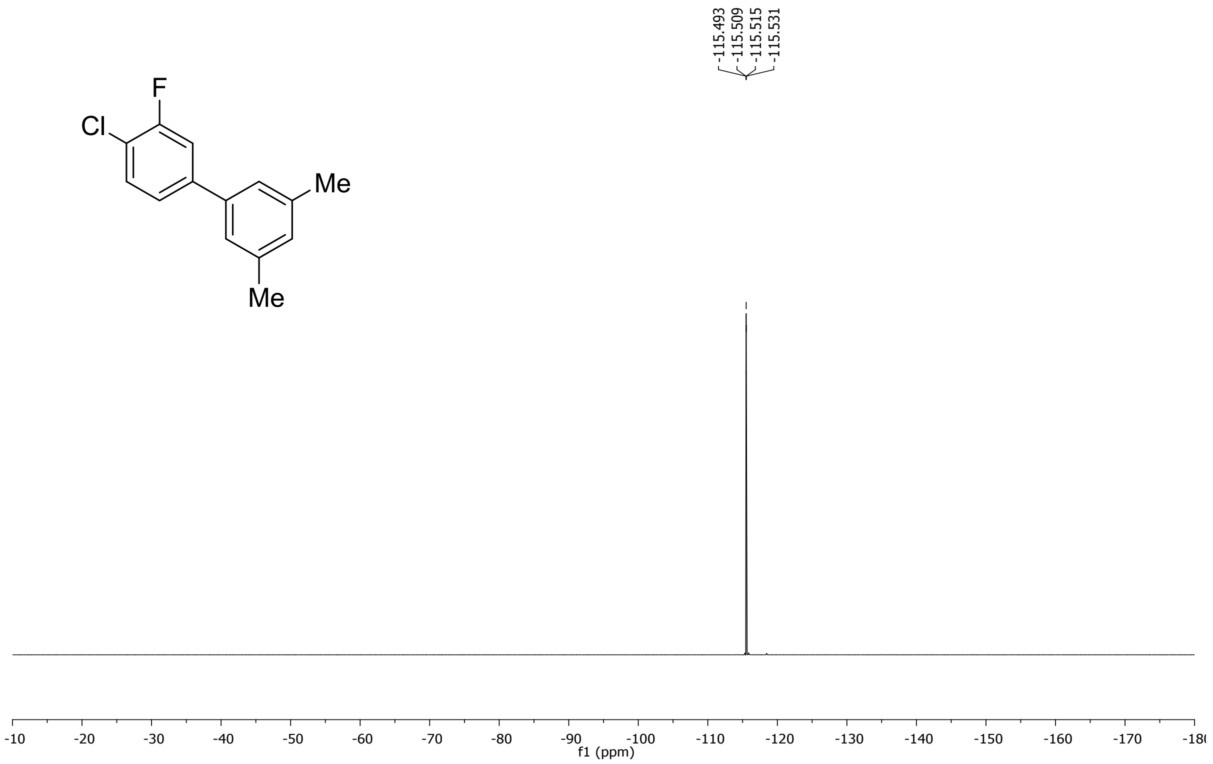

^{1}H ^{1}H COSY

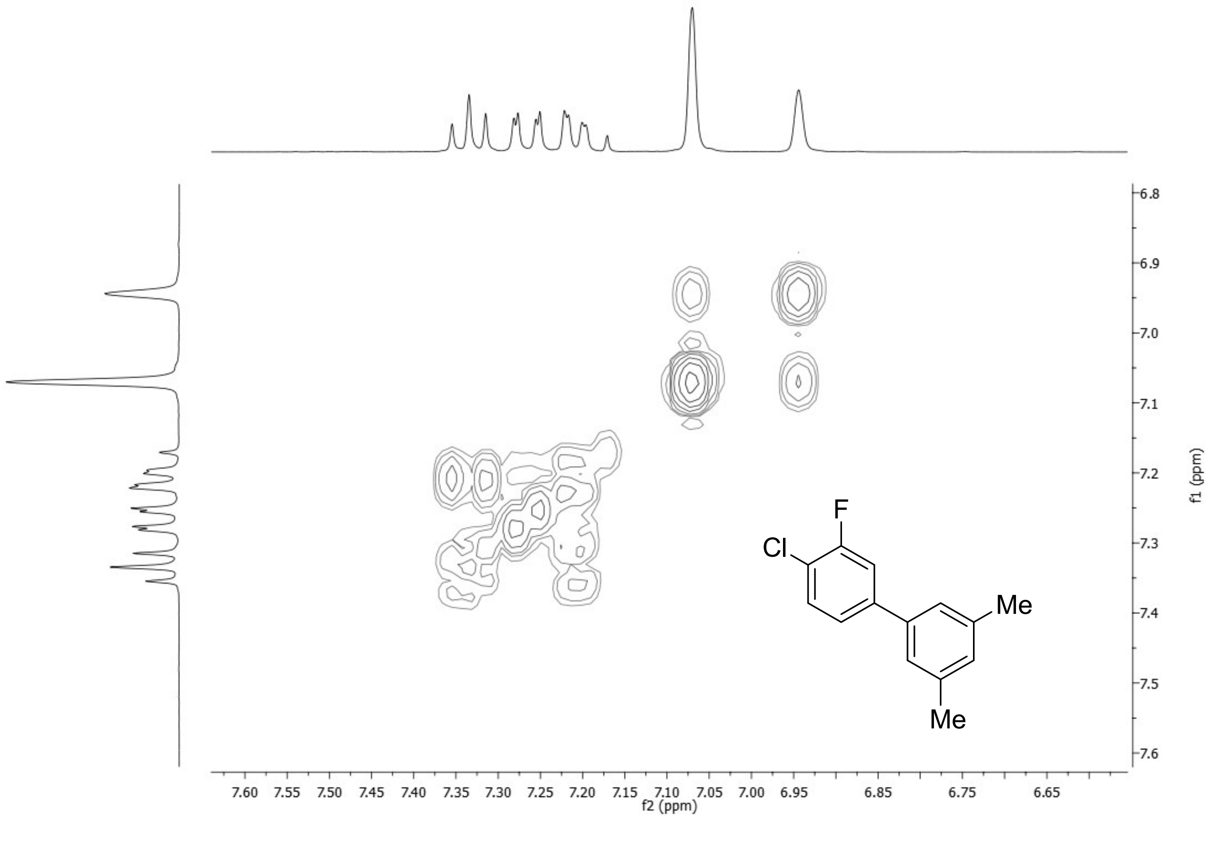

^{1}H ^{13}C HSQC

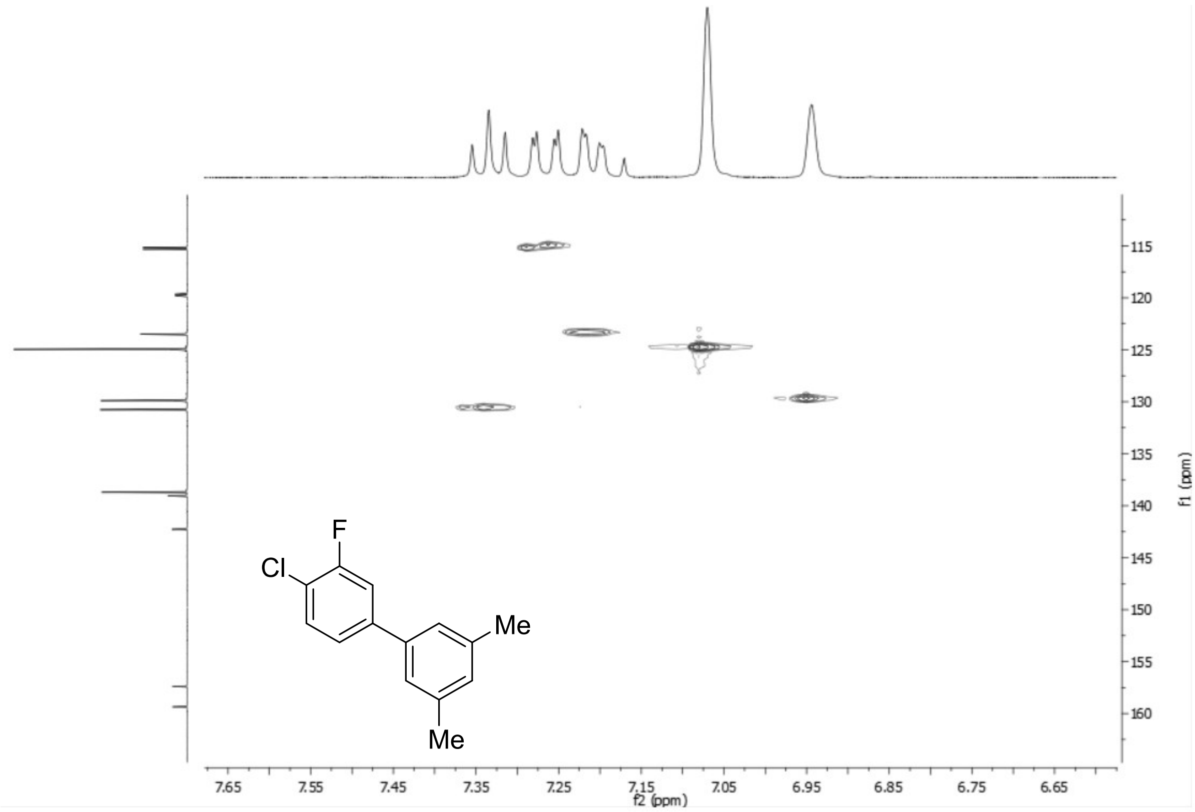

^{1}H NMR

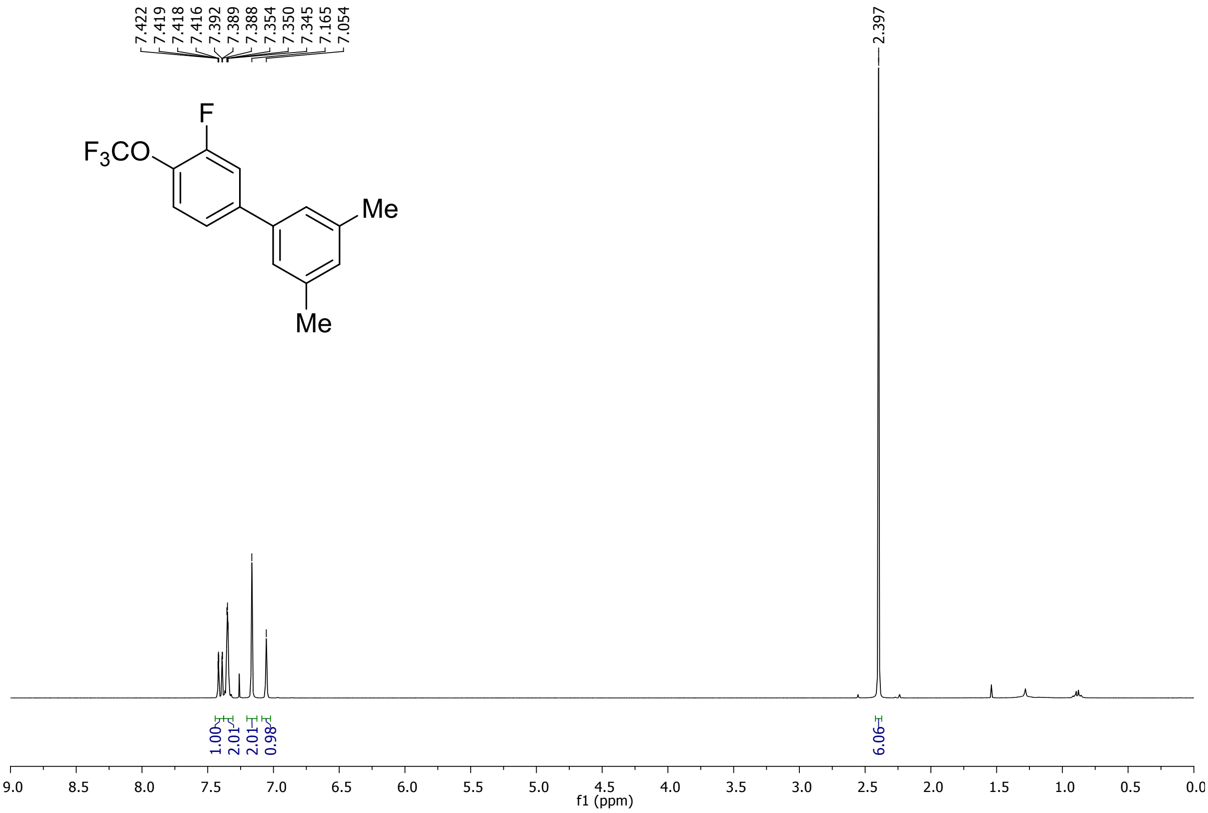

^{13}C NMR

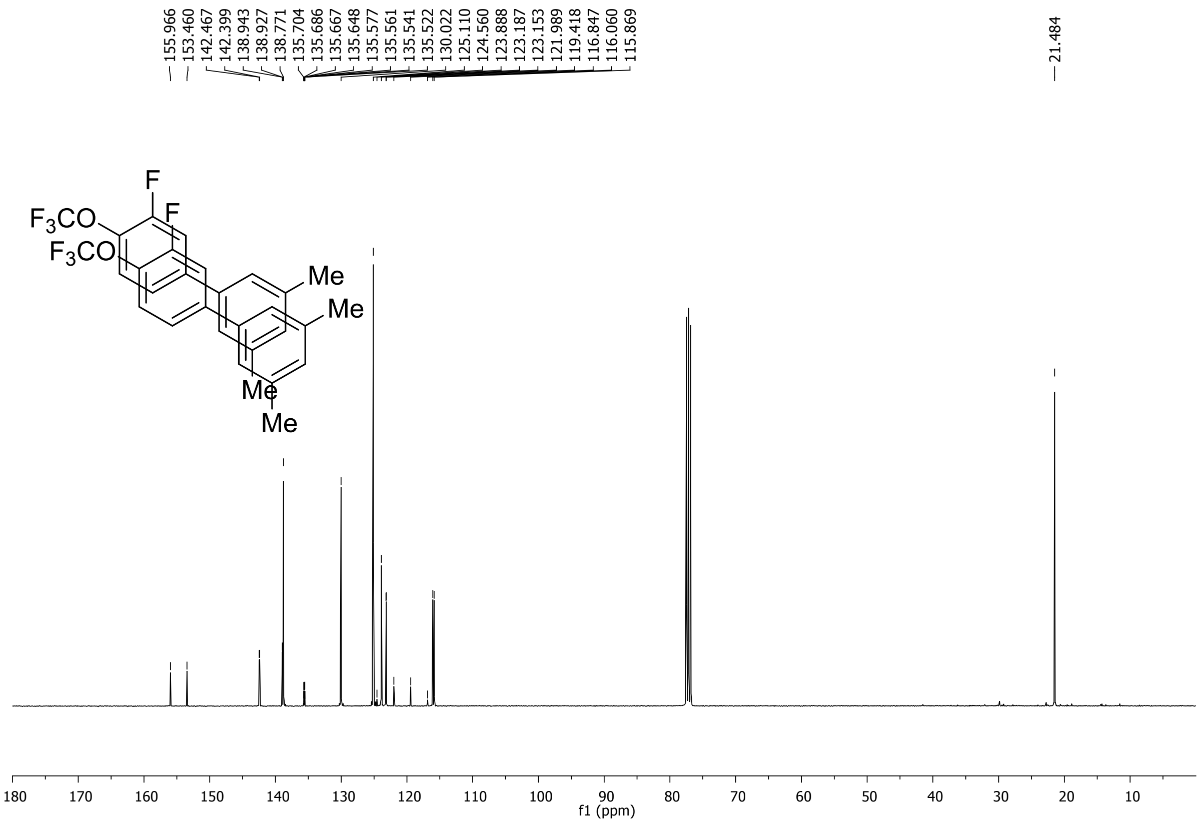

^{19}F NMR

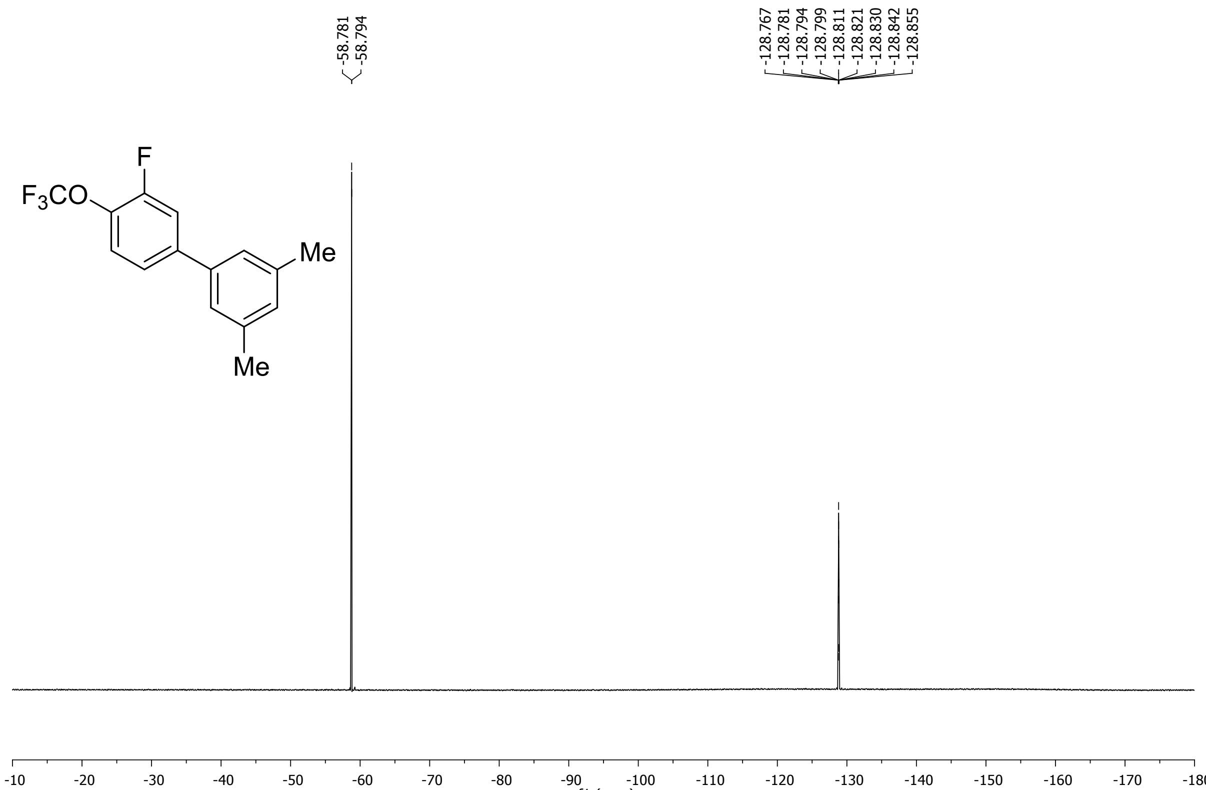

^{1}H ^{13}C HSQC

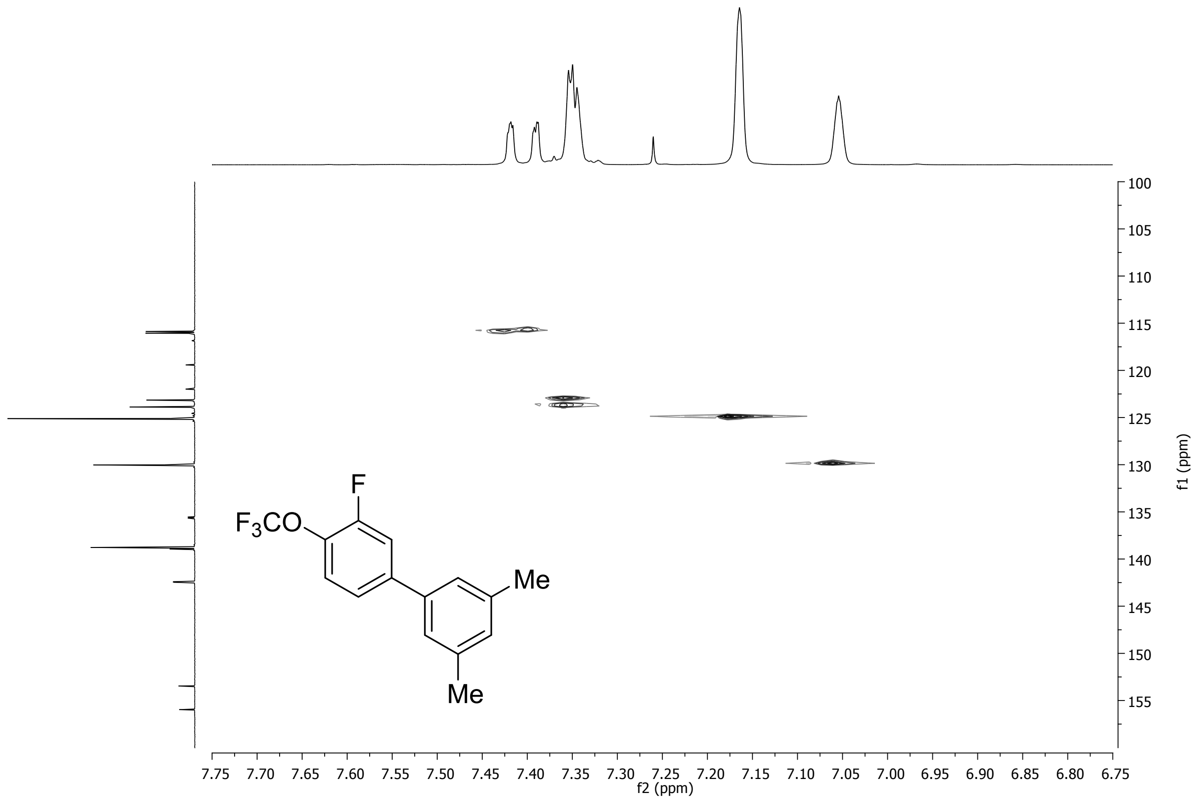

^{1}H NMR

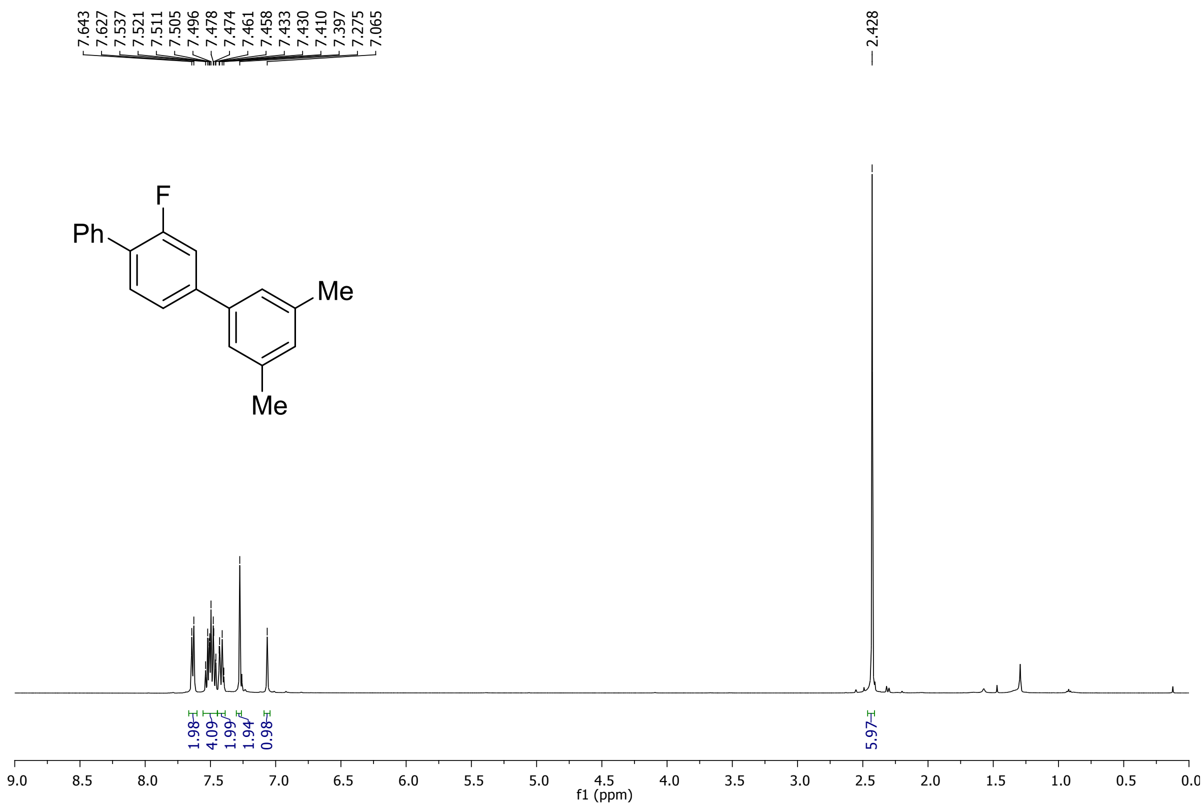

^{13}C NMR

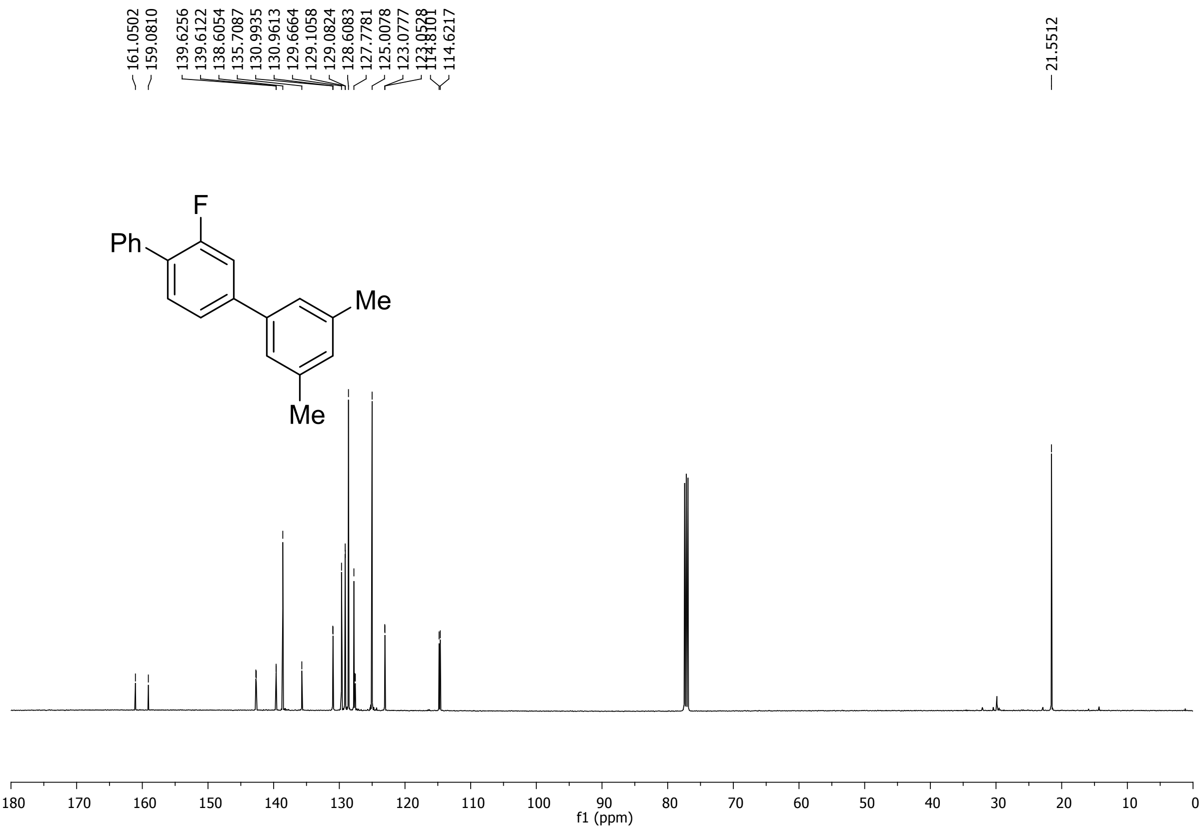

^{19}F NMR

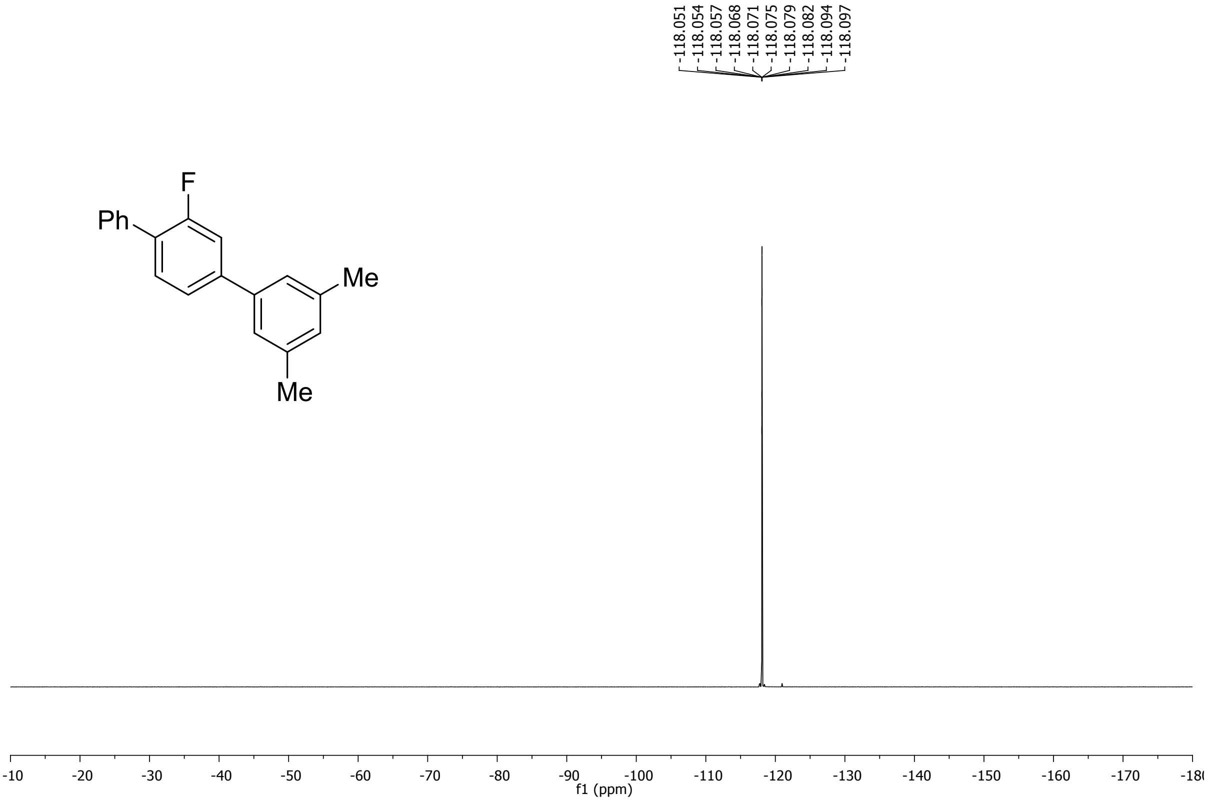

^{1}H NMR

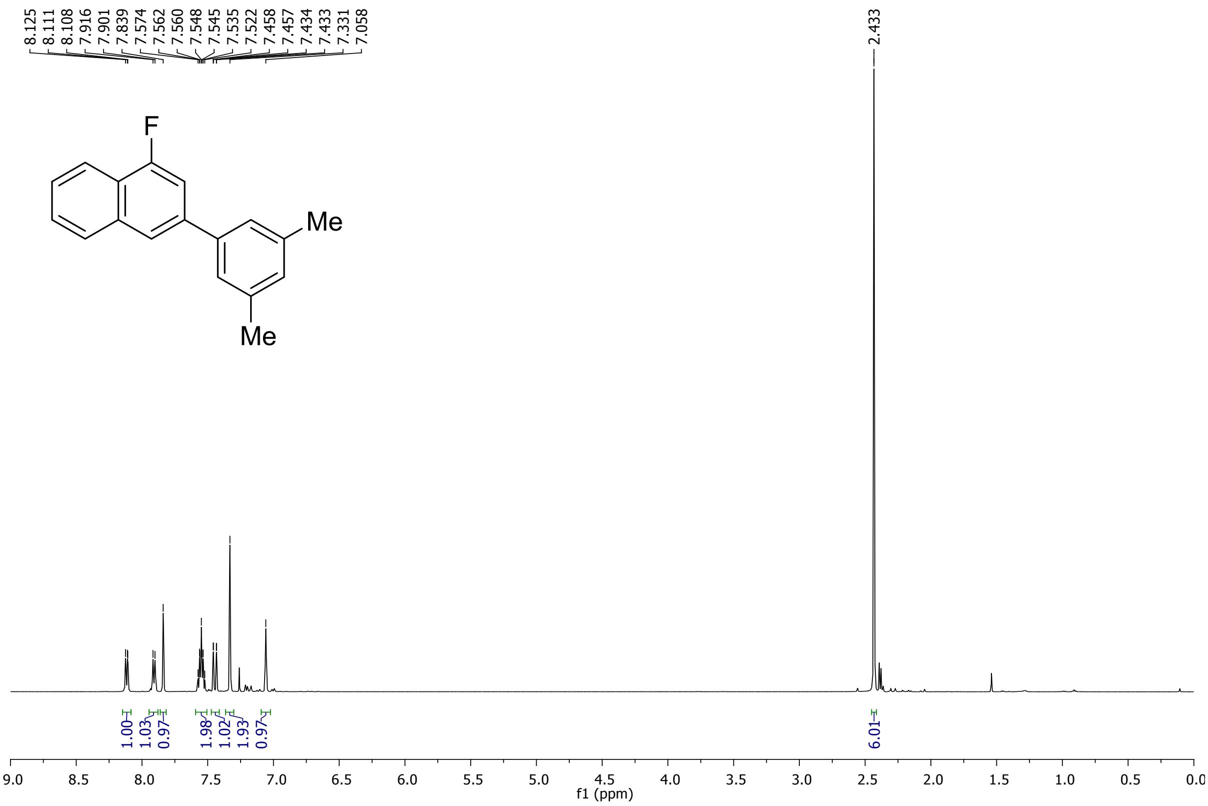

^{13}C NMR

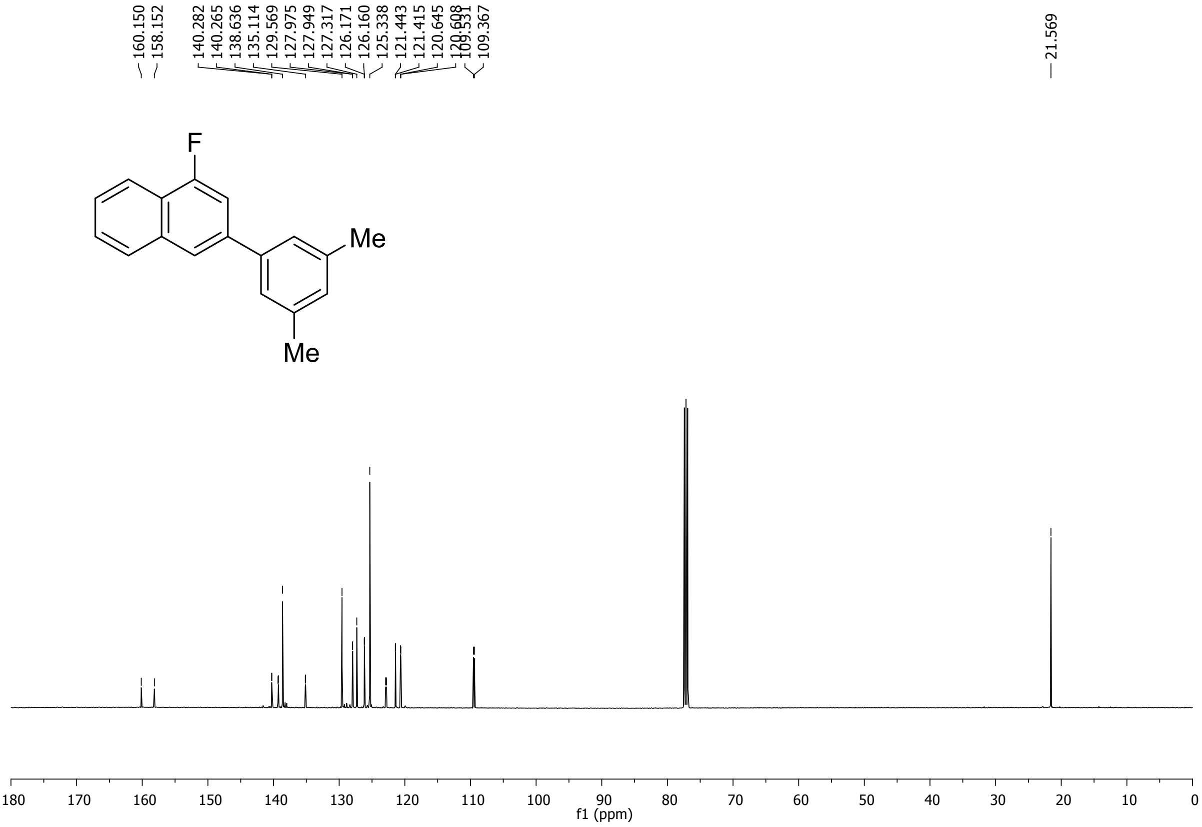

^{19}F NMR

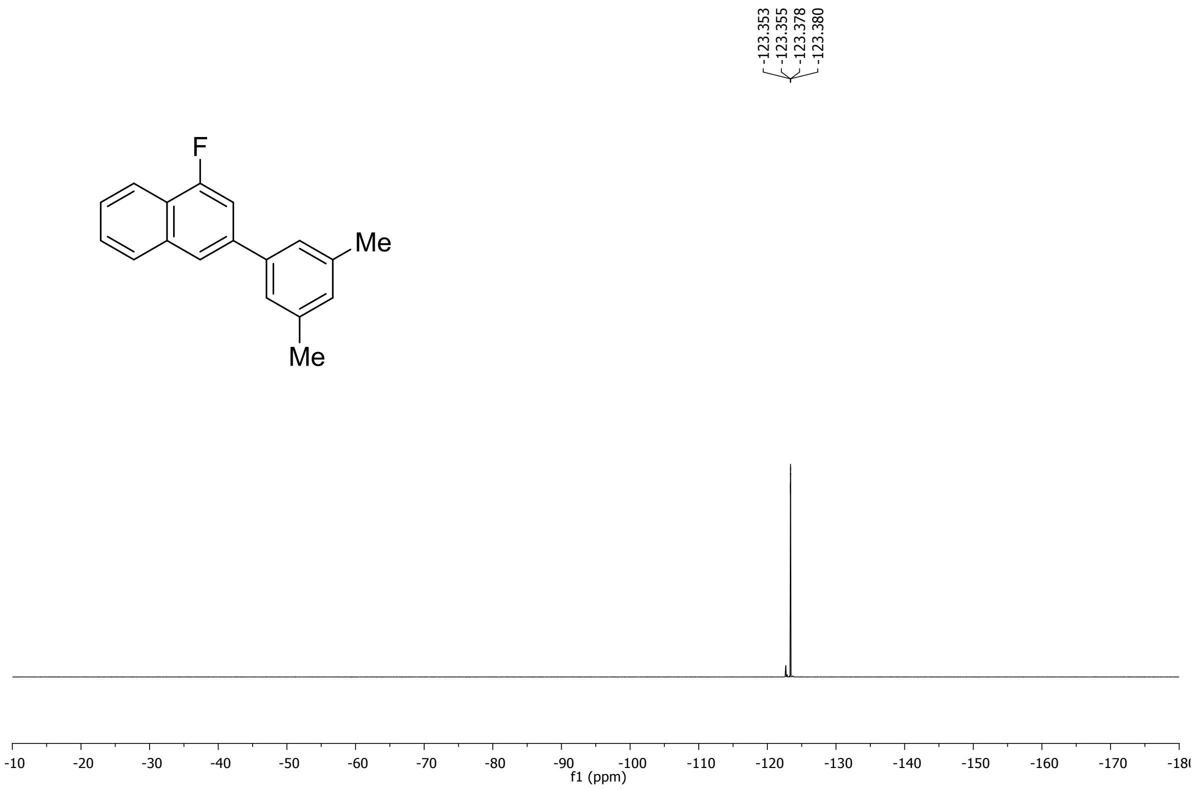

^{1}H NMR

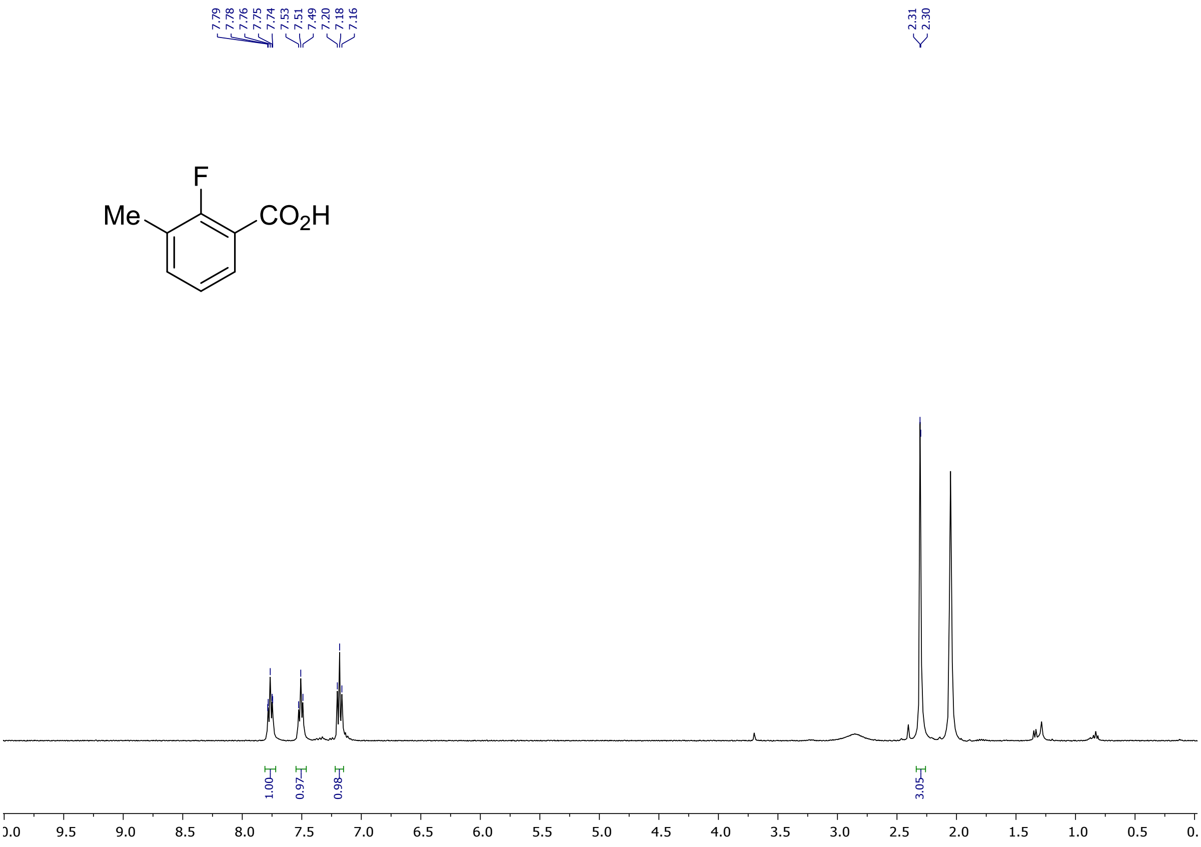

^{19}F NMR

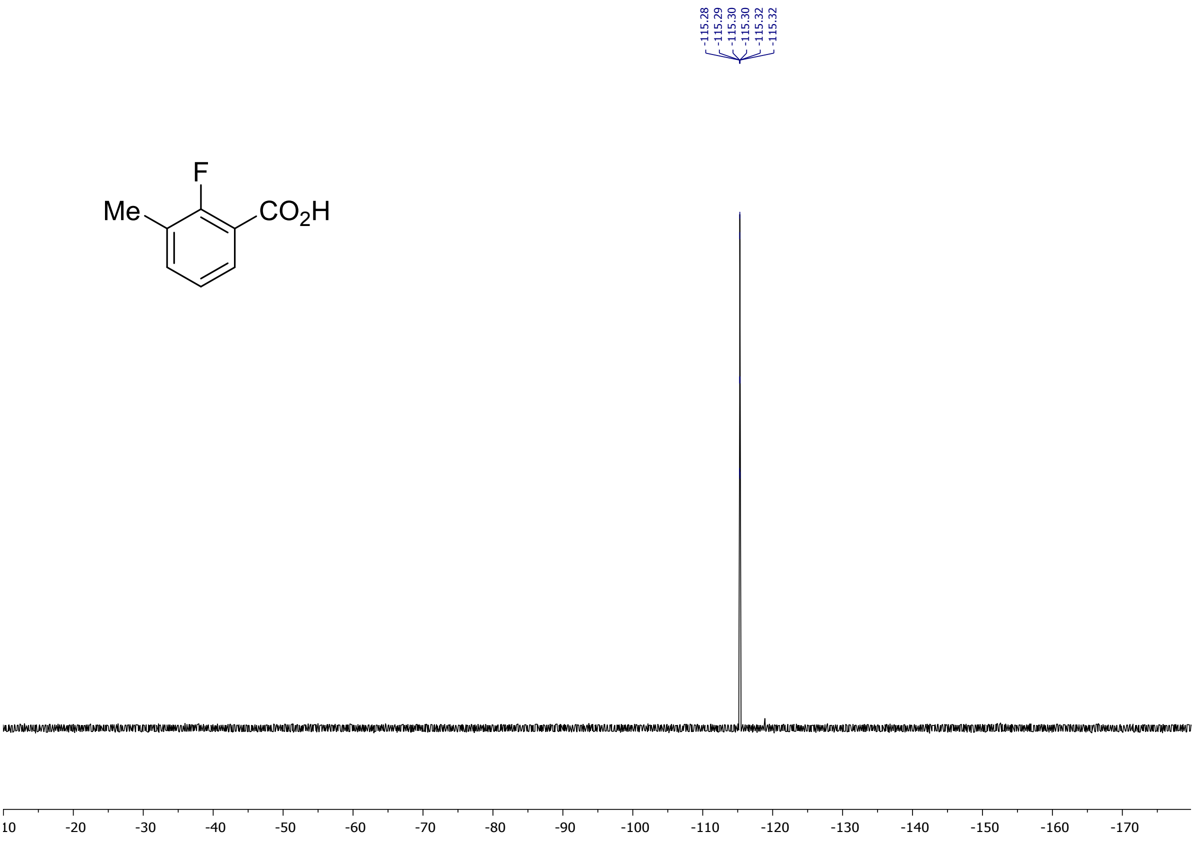

^{1}H NMR

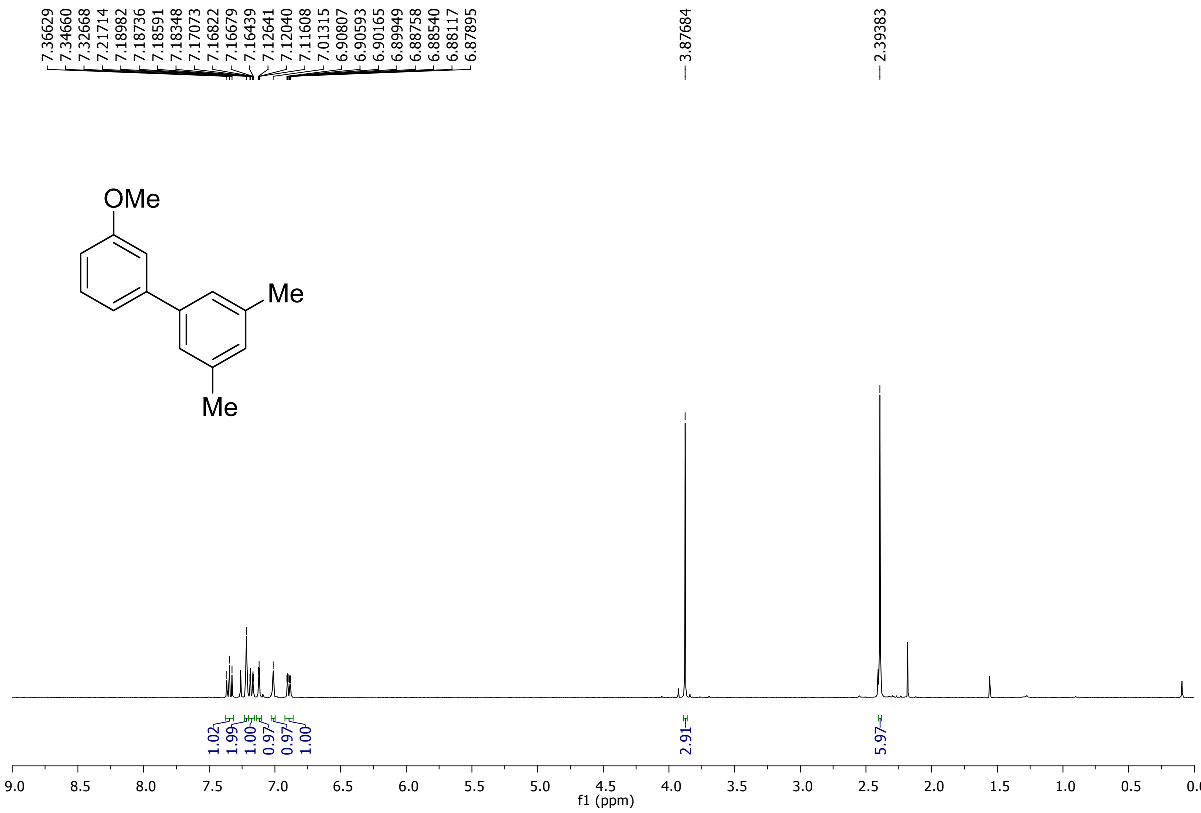

^{13}C NMR

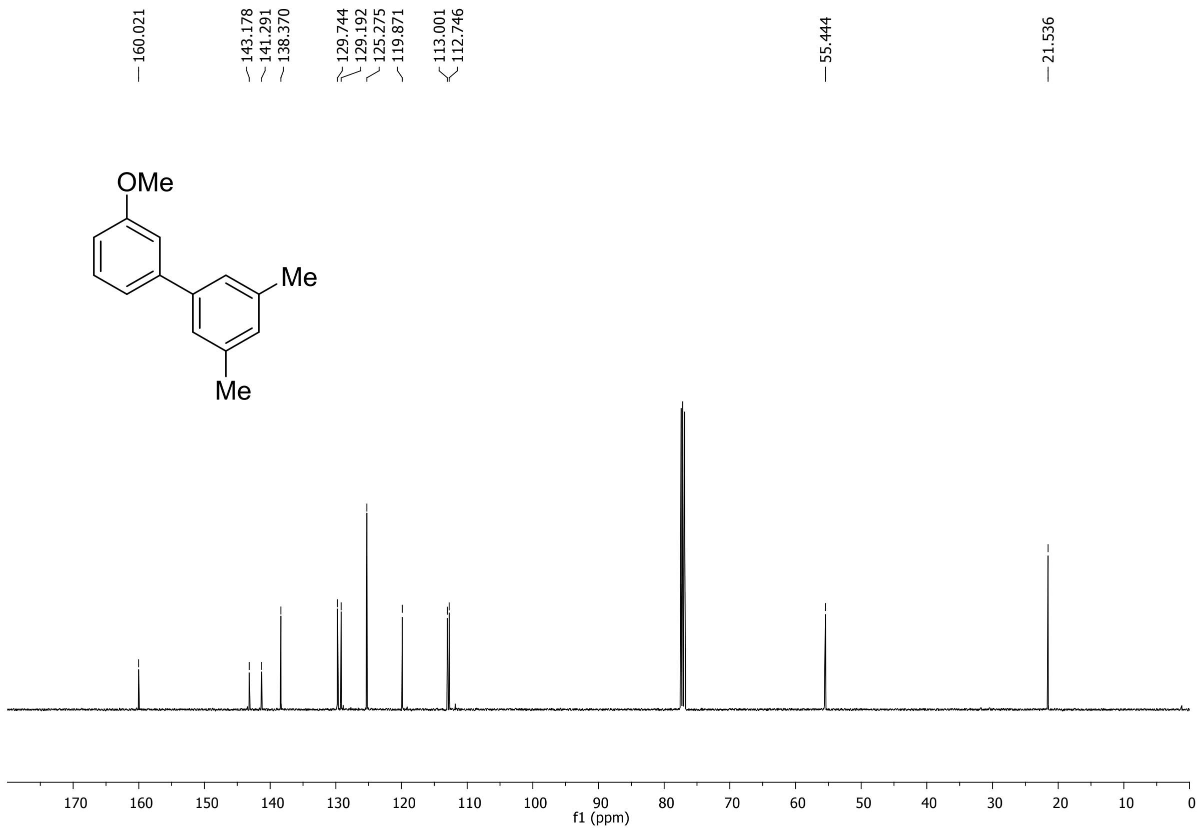

^{1}H NMR

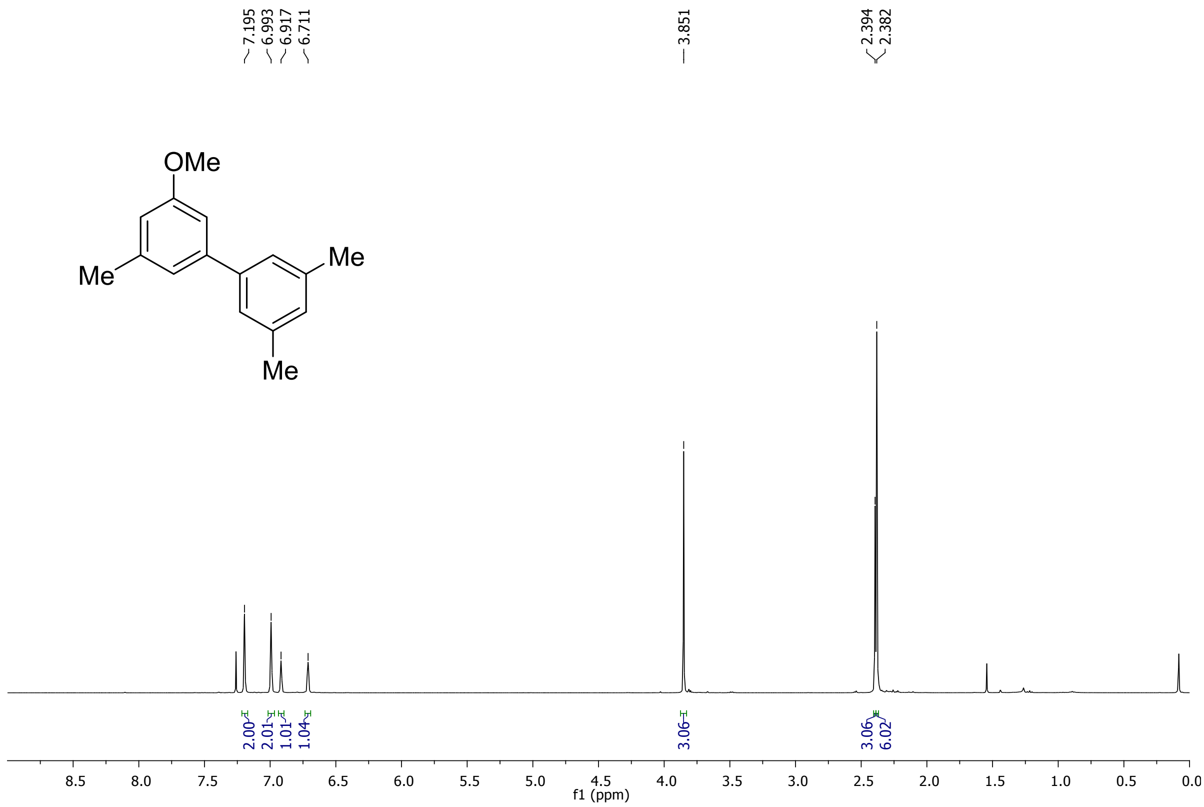

^{13}C NMR

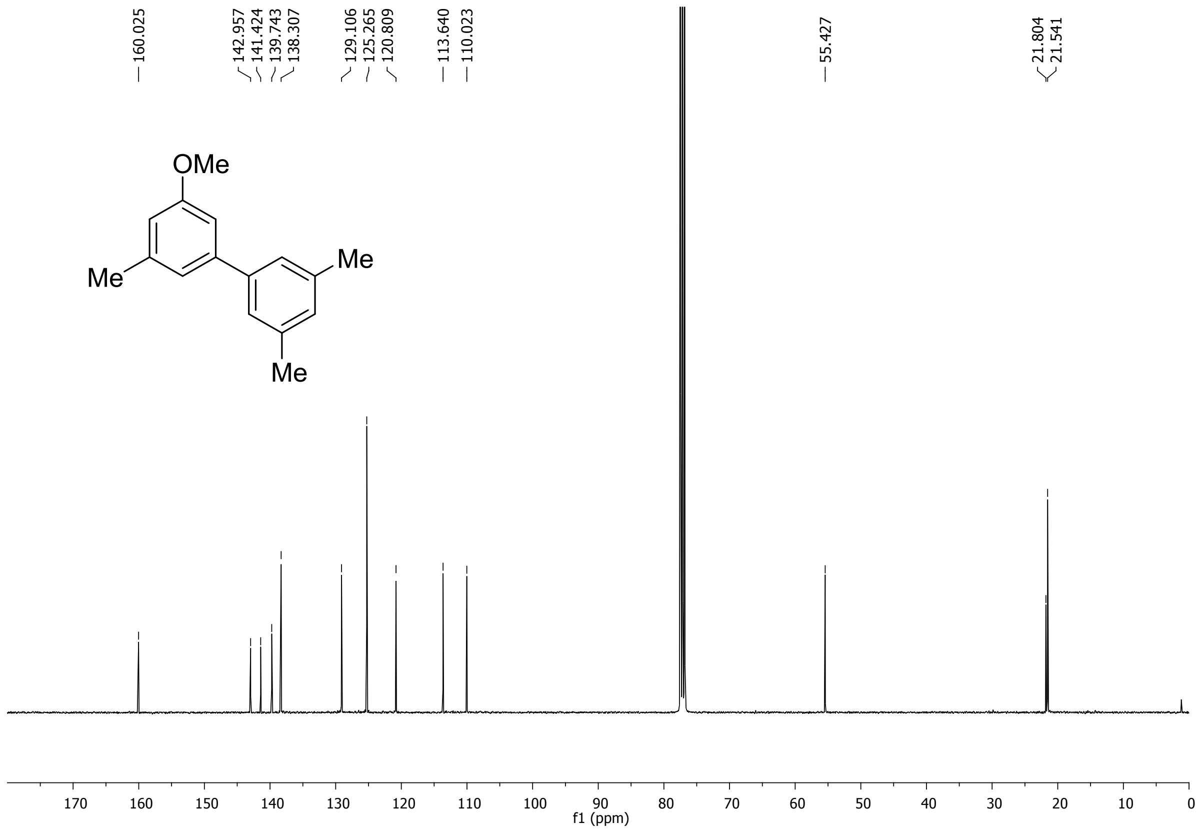

^{1}H NMR

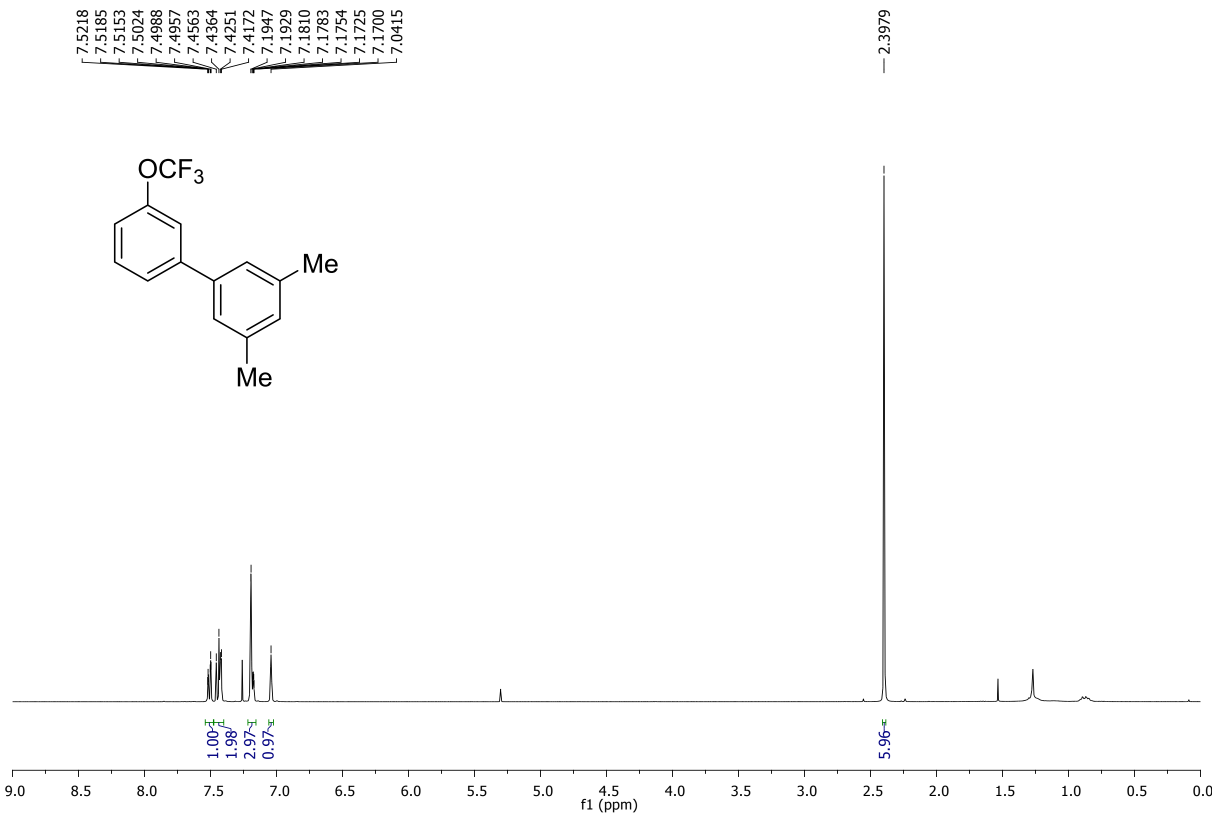

^{13}C NMR

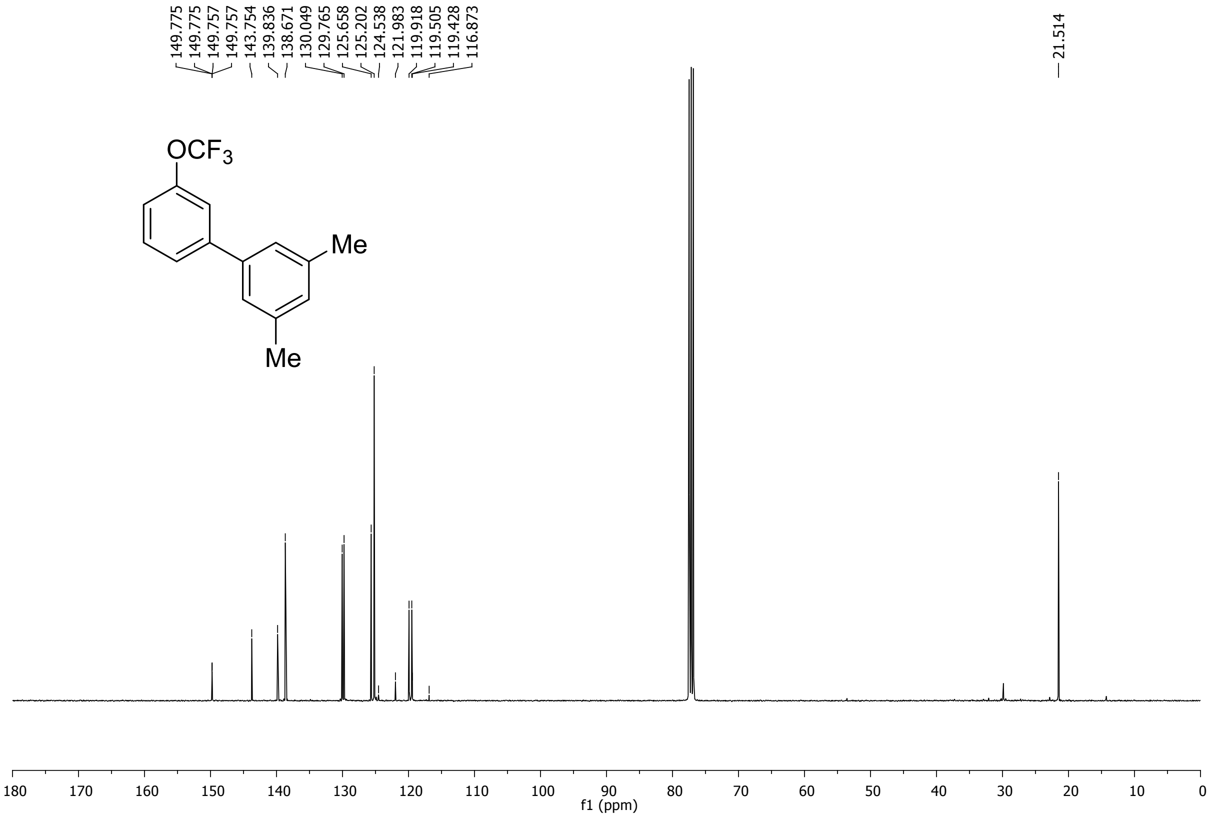

^{19}F NMR

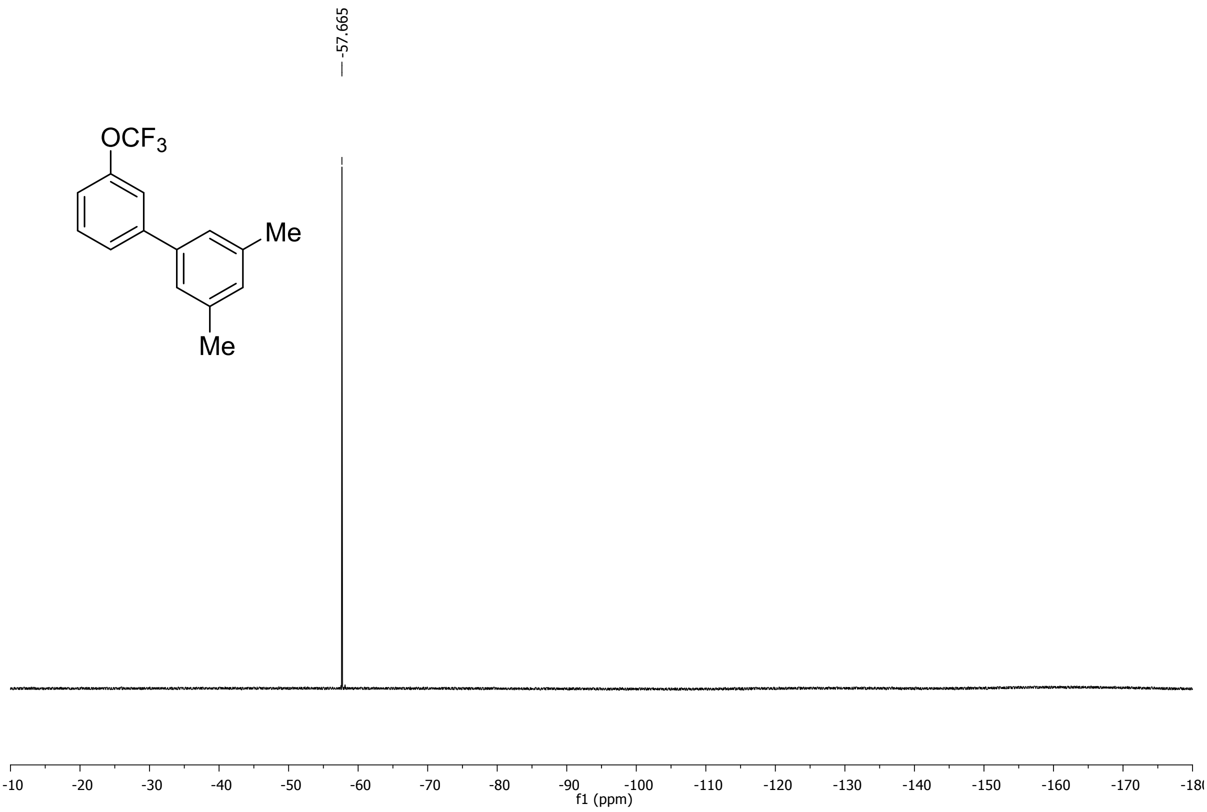

^{1}H NMR

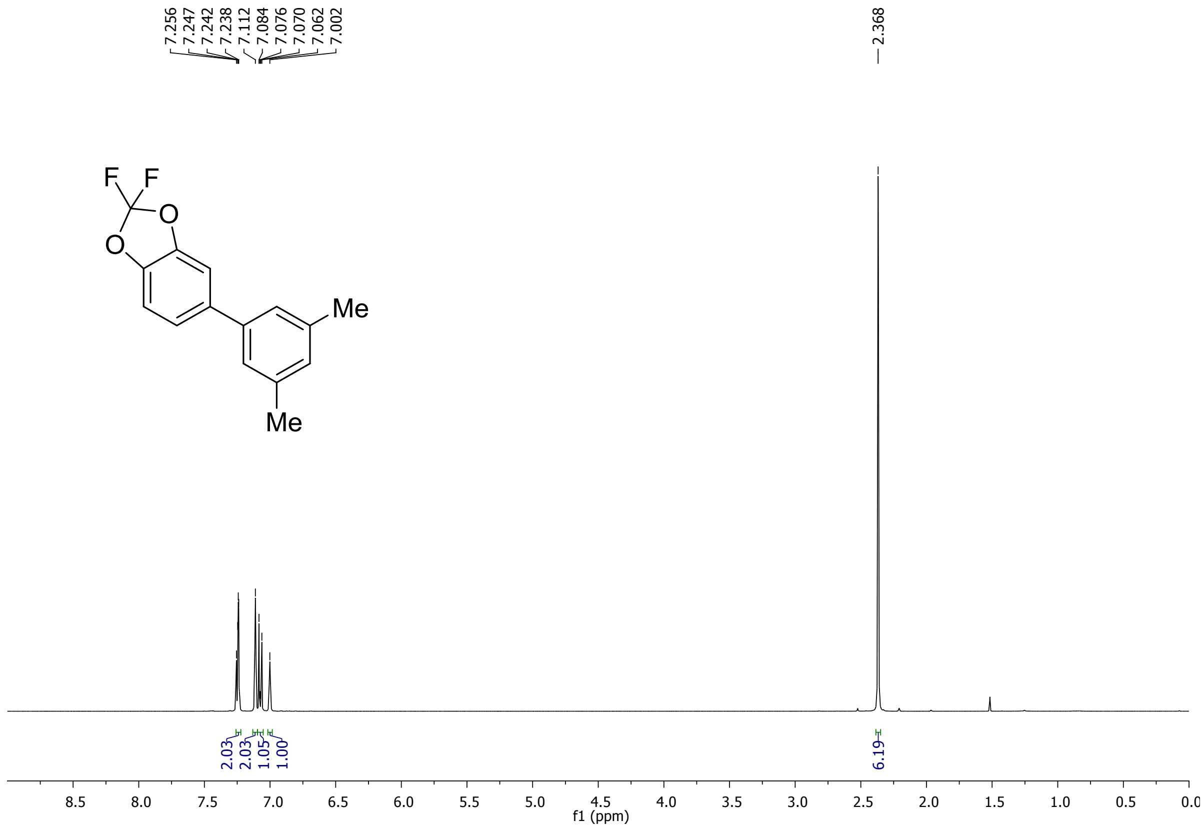

^{13}C NMR

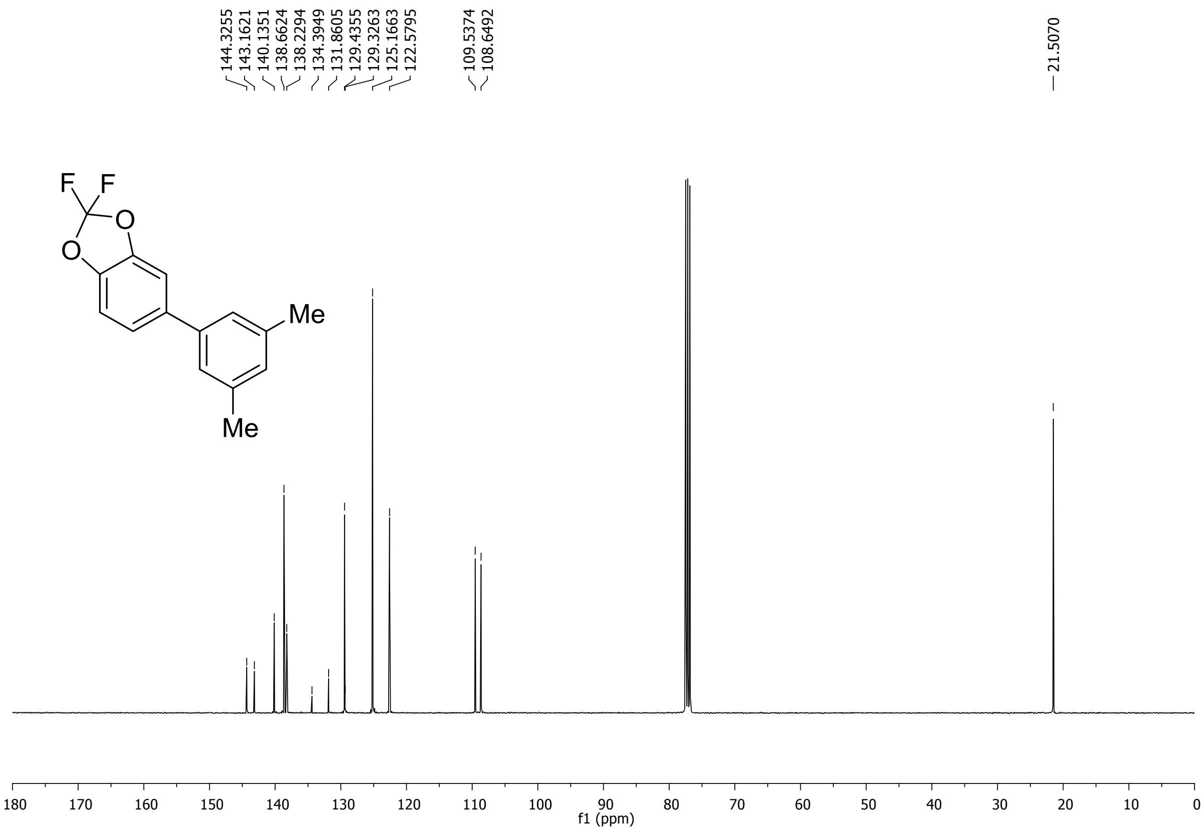

^{19}F NMR

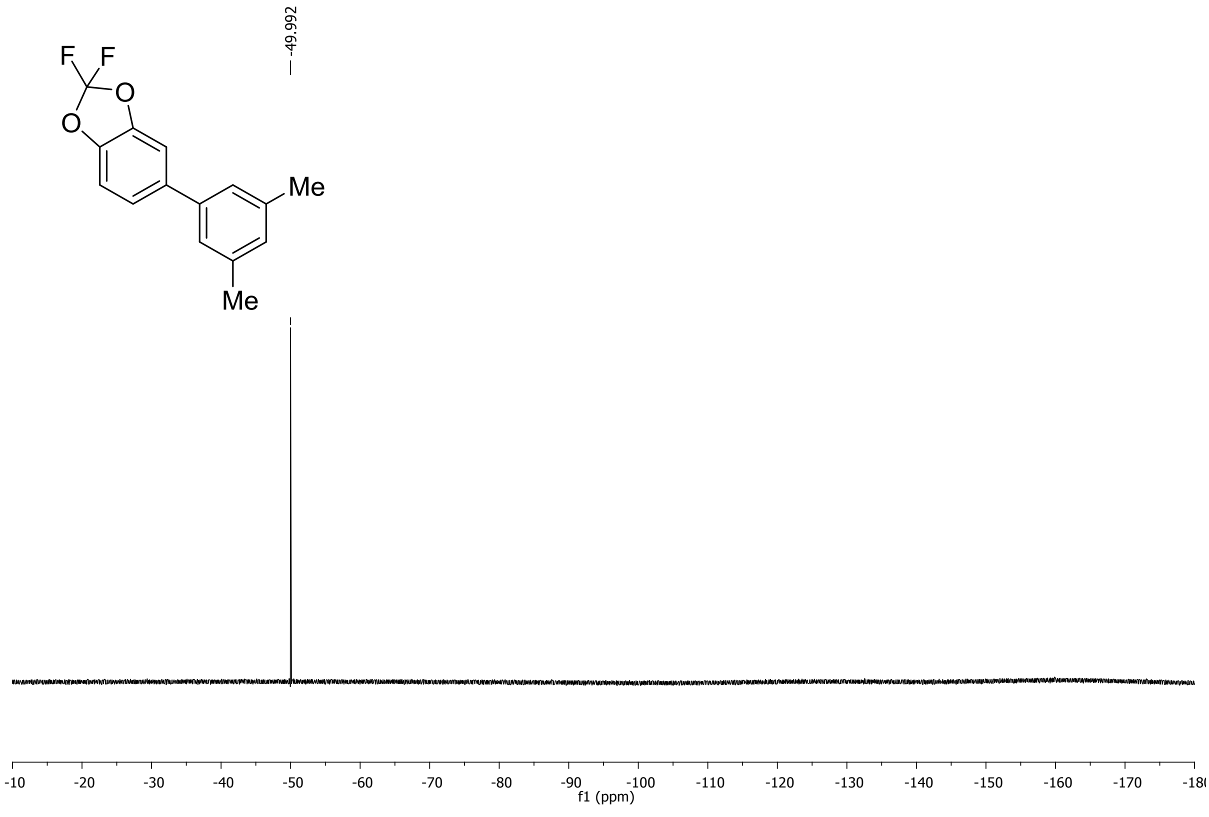

^{1}H NMR

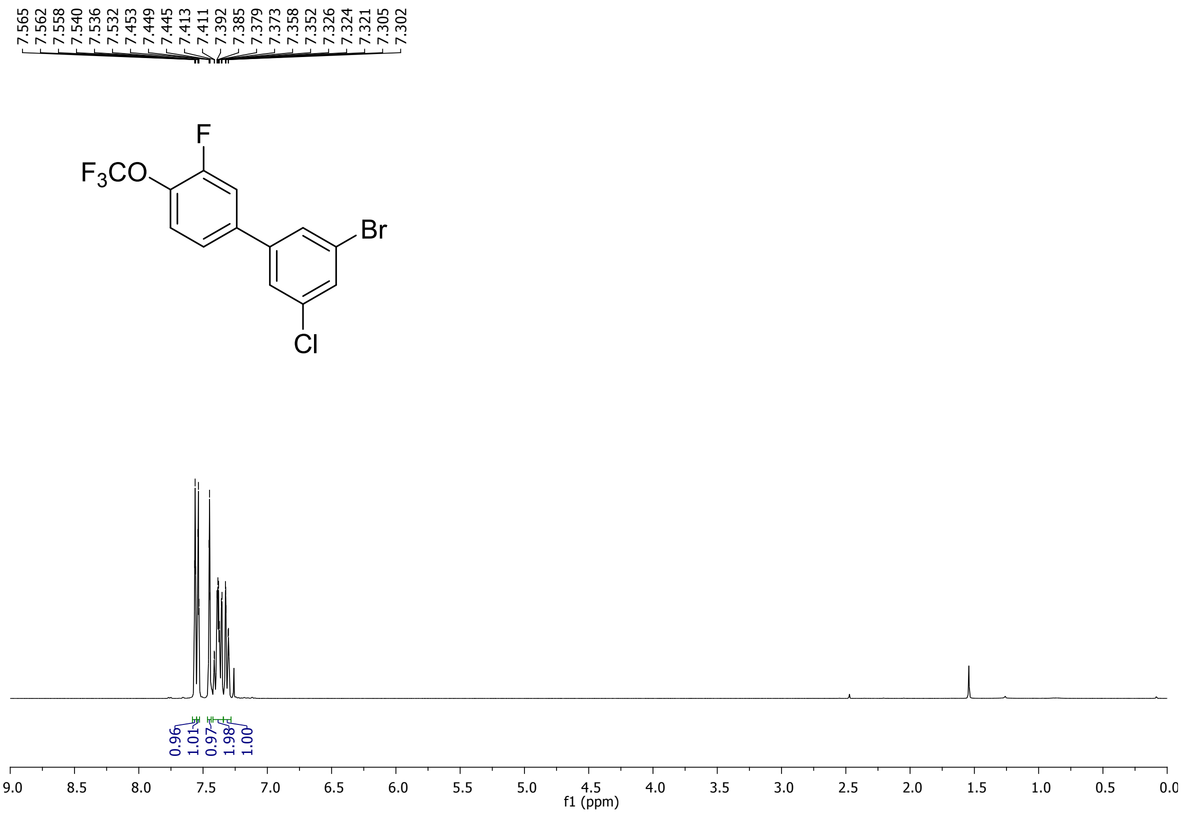

^{13}C NMR

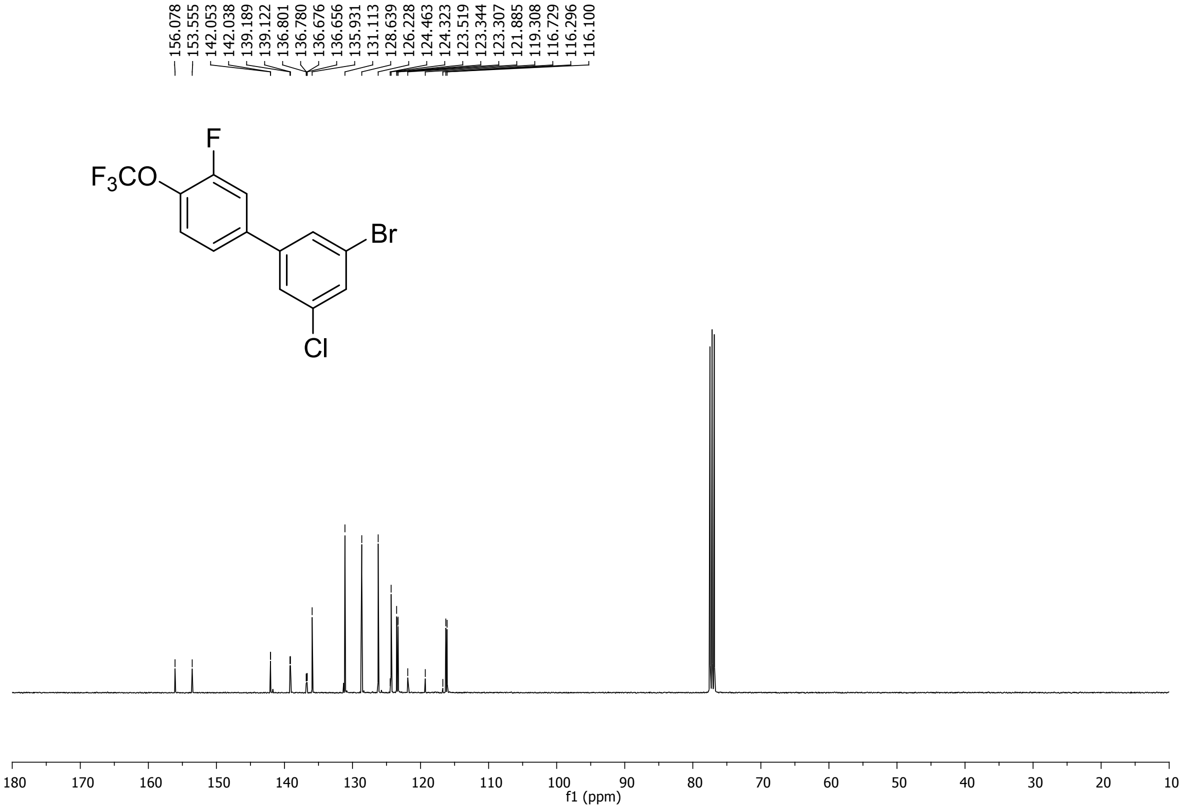

^{19}F NMR

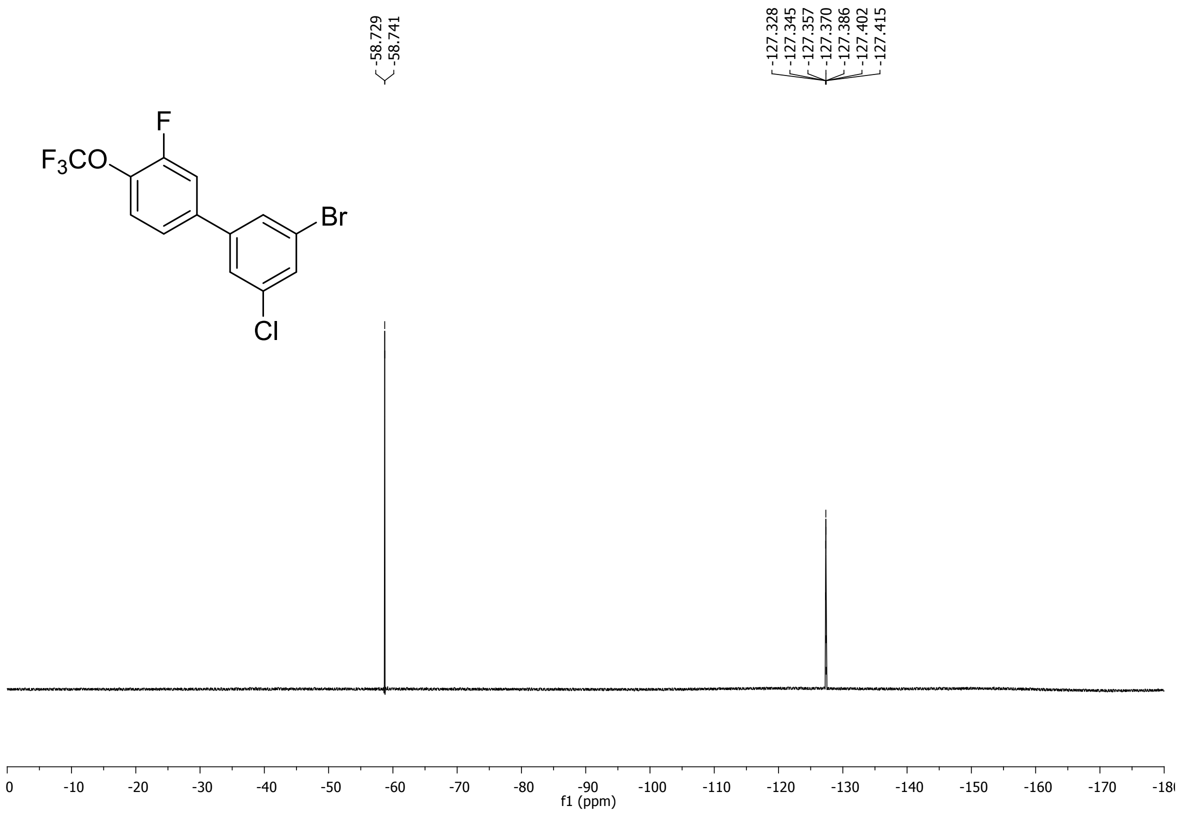

^{1}H NMR

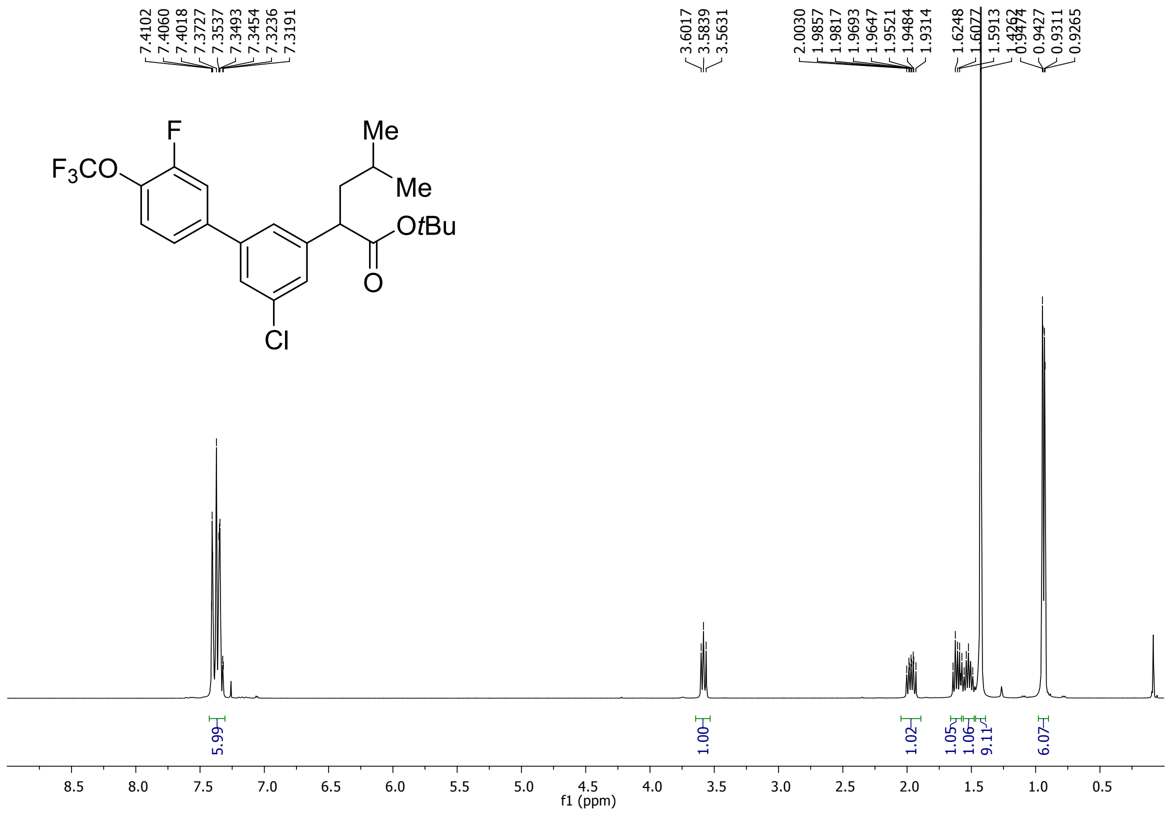

^{13}C NMR

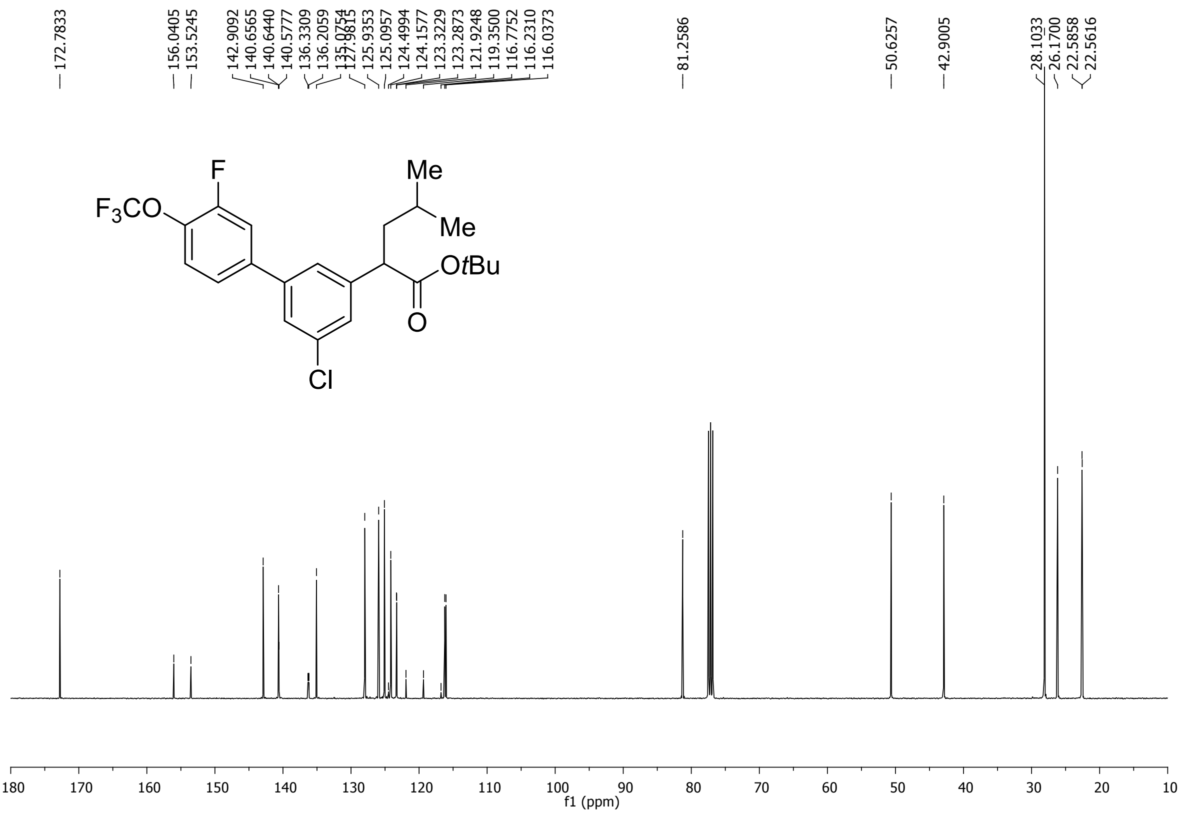

^{19}F NMR

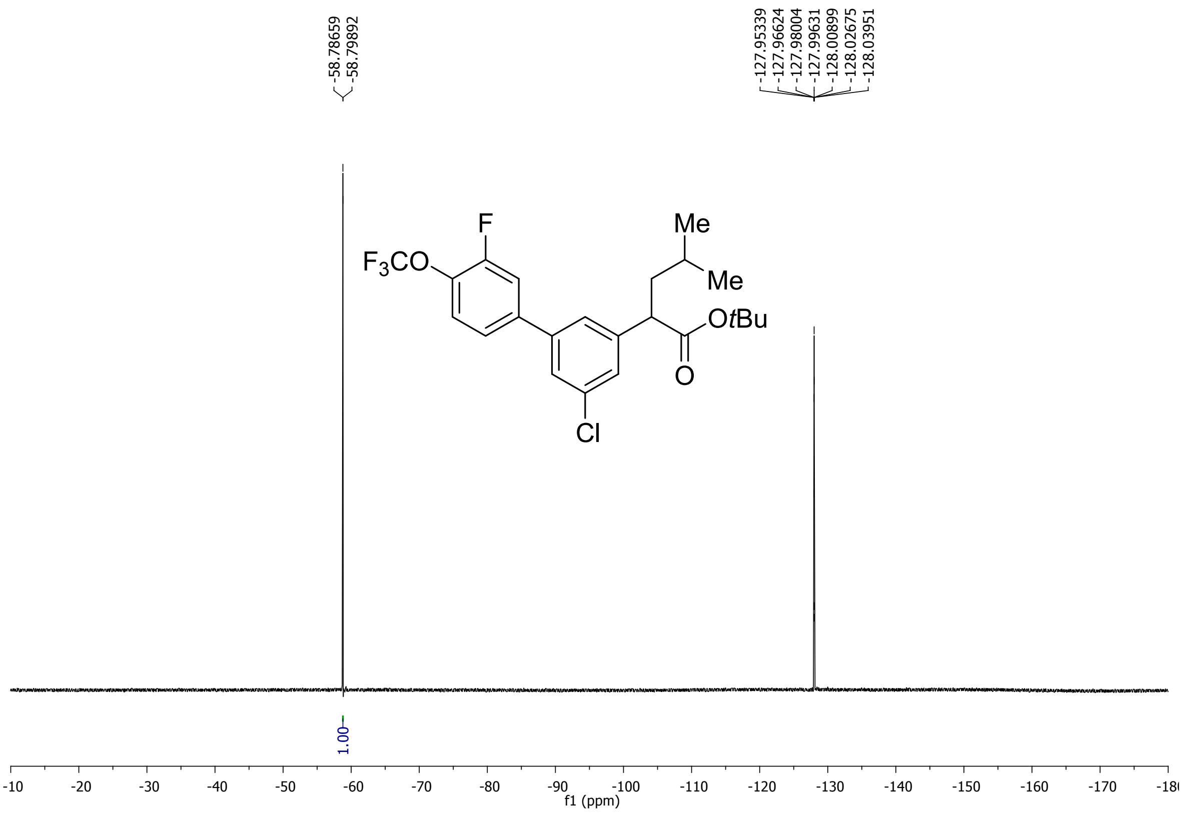

^{1}H NMR

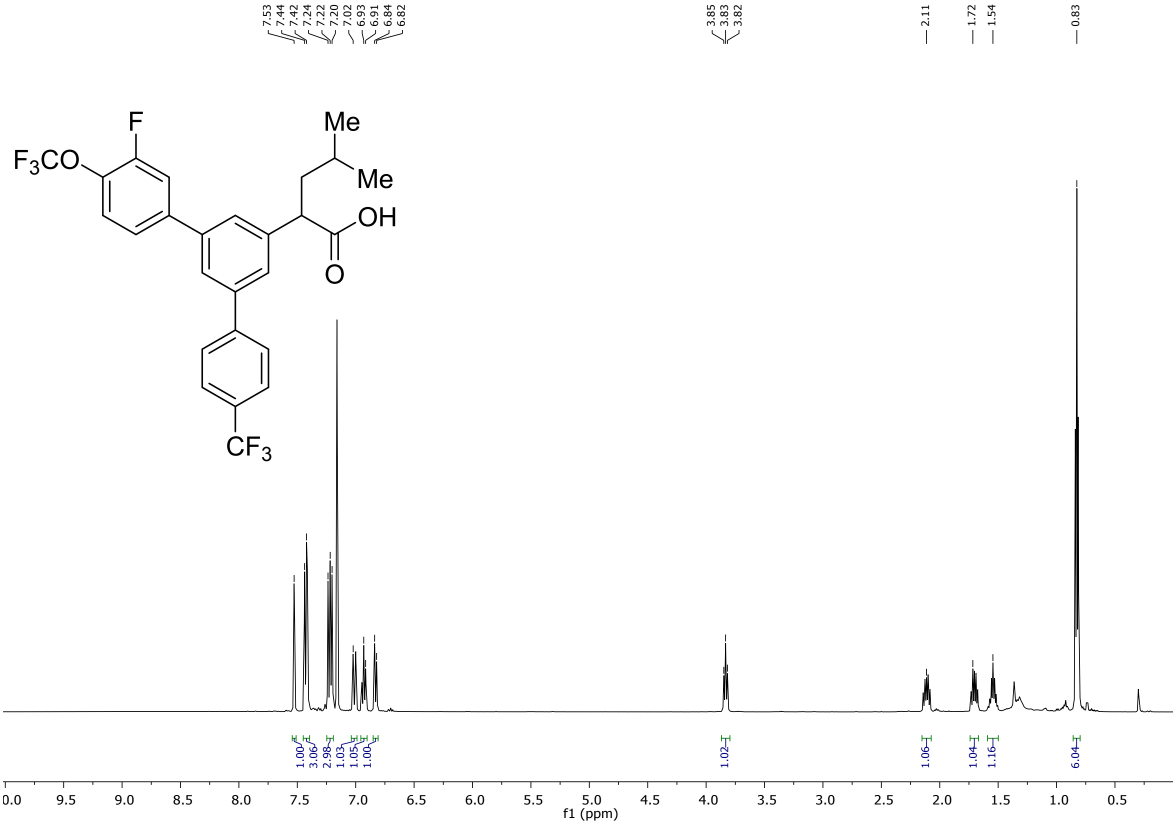

^{19}F NMR

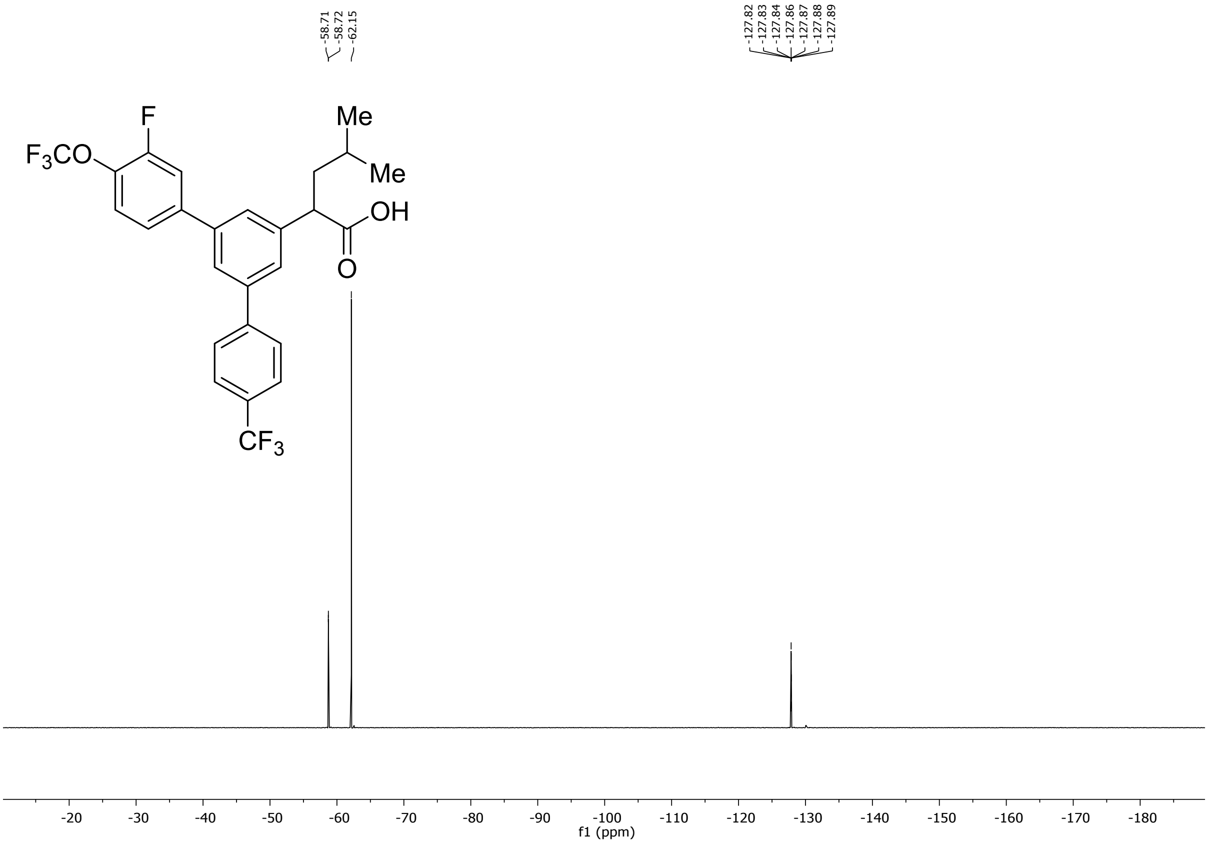

^{13}C NMR

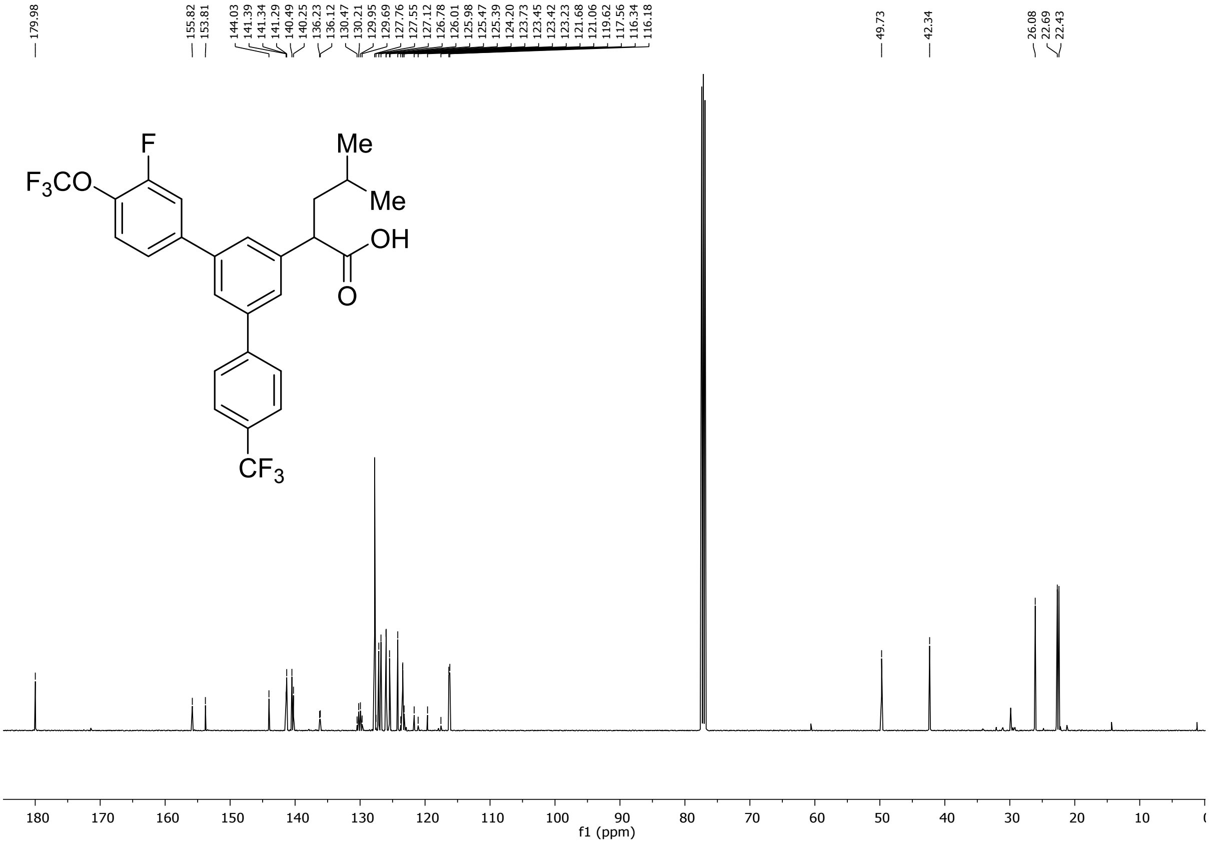

179.98
155.82
153.81
144.03
141.39
141.34
141.29
140.49
140.25
136.23
136.12
130.47
130.21
129.95
129.69
127.76
127.55
127.12
126.78
126.01
125.98
125.47
125.39
124.20
123.73
123.45
123.42
123.23
121.68
121.06
119.62
117.56
116.34
116.18
49.73
42.34
26.08
22.69
22.43
F
F_3CO
Me
Me
OH
O
CF_3
180 170 160 150 140 130 120 110 100 90 80 70 60 50 40 30 20 10
f1 (ppm)
